# Differential effects of class I isoform histone deacetylase depletion and enzymatic inhibition by belinostat or valproic acid in HeLa cells

**DOI:** 10.1186/1476-4598-7-70

**Published:** 2008-09-12

**Authors:** Marielle Dejligbjerg, Morten Grauslund, Thomas Litman, Laura Collins, Xiaozhong Qian, Michael Jeffers, Henri Lichenstein, Peter Buhl Jensen, Maxwell Sehested

**Affiliations:** 1 Experimental Pathology Unit, National University Hospital, Biocentre, Ole Maaloes Vej 5, 2200 Copenhagen, Denmark; 2 TopoTarget A/S, Symbion Science Park, Fruebjergvej 3, 2100 Copenhagen, Denmark; 3 Exiqon A/S, Bygstubben 9, 2945 Vedbaek, Denmark; 4 Topotarget UK Ltd, 87a Milton Park, Abingdon, Oxon, OX14 4RY, UK; 5 CuraGen Corporation, 322 East Main Street, Branford, CT 06405, USA; 6 Dept. Oncology, Finsen Centre, National University Hospital, 2100 Copenhagen, Denmark

## Abstract

**Background:**

Histone acetylation is an epigenetic modification involved in the regulation of gene expression, balanced by histone acetyl transferases and histone deacetylase (HDAC) enzymes. HDAC inhibitors (HDACi) induce growth arrest and cell death in transformed cells, and are currently in many clinical cancer trials. The transcriptional response to HDACi is complex, as is the response to HDAC isoform knockdown (KD). Here, we describe for the first time in a human cancer cell line, a transcriptional comparison of treatment by two structurally unrelated HDACi; belinostat and valproic acid with the KD of HDAC1, 2 and 3 isoforms.

**Results:**

HDAC KD showed anti-proliferative effects, although to a lesser extent than HDACi treatment. Moreover, we found a 2-fold increased resistance of HDAC1 knockdown cells to belinostat, suggesting this isoenzyme as a selective target. While both HDACi treatment and individual class I HDAC KD produce significant transcriptional effects, three-times higher for HDACi, the gene-expression profiles of class I HDAC KD compared with that obtained by HDACi treatment exhibited less than 4% of altered genes in common between the two modes of inhibition. Further, cell-specific effects of HDAC KD are evident by comparison with a recent study in a different cell line.

**Conclusion:**

The increased resistance to belinostat in response to HDAC1 depletion indicates the possibility of using this isoform as a predictive biomarker of response to HDACi treatment. Further, the transcriptional response to chemical inhibition of HDACs is very different from that of KD of individual class I HDAC isoforms. These data suggest that the anti-tumor effect of HDACi is indeed linked to class I inhibition, but may be more complex than simply targeting individual HDAC enzymes.

## Background

The transcription of genes is highly regulated by epigenetic chromatin modifications, including the acetylation of lysine residues protruding from nucleosomal histones. Thus, histone acetylation status is maintained by the opposing actions of histone acetyl transferase and histone deacetylase (HDAC) enzymes [1,2]. HDACs modify gene expression via multiple mechanisms. The deacetylation of histones causes general chromosome condensation, and also plays a role in transcriptional regulation by forming a combinatorial 'histone code' that regulates downstream responses [2,3]. Additionally, a variety of non-histone targets such as transcription factors, structural and chaperone proteins are targeted by HDAC enzymes [4]. The Zn^2+^-dependent mammalian HDAC isoenzymes are divided into three classes based on their homology to yeast deacetylase proteins. Class I HDAC isoforms include HDAC1, -2 and -3 that are ubiquitously expressed as well as the low-abundance HDAC8. Class II (HDAC4, 5, 6, 7, 9, 10) and IV (HDAC11) isoforms display a more restricted tissue pattern of expression [1]. A number of cofactors are required for HDAC activity; indeed, they reside in multi-protein complexes including co-regulators and other chromatin-modifying enzymes [2].

Recent advances into the biology of HDAC enzymes reveal a substantial division of labor between HDAC subtypes [2,5]. Modulating HDAC expression demonstrates that class I HDACs are essential for proliferation and survival. Hence, HDAC1 and HDAC3 are believed to be important for proliferation [6-9], whereas HDAC2 is likely involved in the regulation of apoptosis [10,11]. HDAC8 has been implicated in smooth muscle cell contractility [12], though its knockdown (KD) also affects proliferation in tumor cells [13]. Class II HDACs are mainly involved in cell differentiation and development [14], while selective HDAC6 inhibition by tubacin also induced cytotoxicity without accompanying gene-expression changes [15]. Aberrant expression of HDAC1, 2, 3 and 6 has been observed in various tumor types [16-21], and HDAC2-mutant mice display reduced tumor development [22]. Further, the transformed epigenome of neoplastic cells includes specific hypo-acetylation of histone H4 [23]. Together, these findings provide the rationale for the targeted inhibition of HDAC enzymes. HDACi treatment increases global acetylation levels, which ultimately results in cell cycle arrest, apoptosis or terminal differentiation of transformed cells. A considerable variation in the gene-expression response to HDACi depending on cell line and structural class of drug has been demonstrated, and because HDACi treatment potentially affects the entire transcriptome, it is interesting that pan-HDAC inhibition changes the expression of a relatively small percentage of genes [24,25]. There are several structurally distinct HDACi currently in clinical trials for the treatment of solid and hematological cancers, of which the hydroxamate Zolinza (vorinostat, SAHA), recently gained approval for the treatment of cutaneous T-cell lymphoma [26].

Despite several reports on the effects of HDAC KD in human and other species, a direct comparison of global gene-expression changes between individual class I HDAC KD and HDACi treatment has not previously been performed on human cancer cell lines. In this report, we examined viability parameters and transcriptional profiles of human HDAC1, 2 and 3 KD, and directly compared expression profiles with treatment of near-IC50 doses of two structurally distinct HDACi; the pan-inhibitory hydroxamate belinostat (PXD101) and the class I selective short-chain fatty acid valproic acid (VPA) [26]. Further, we compared HeLa class I HDAC KD microarray data with that obtained in a recent similar study on U2OS cells.

## Results

### Depletion of HDAC1, 2 and 3 affect viability

**Figure 1:**
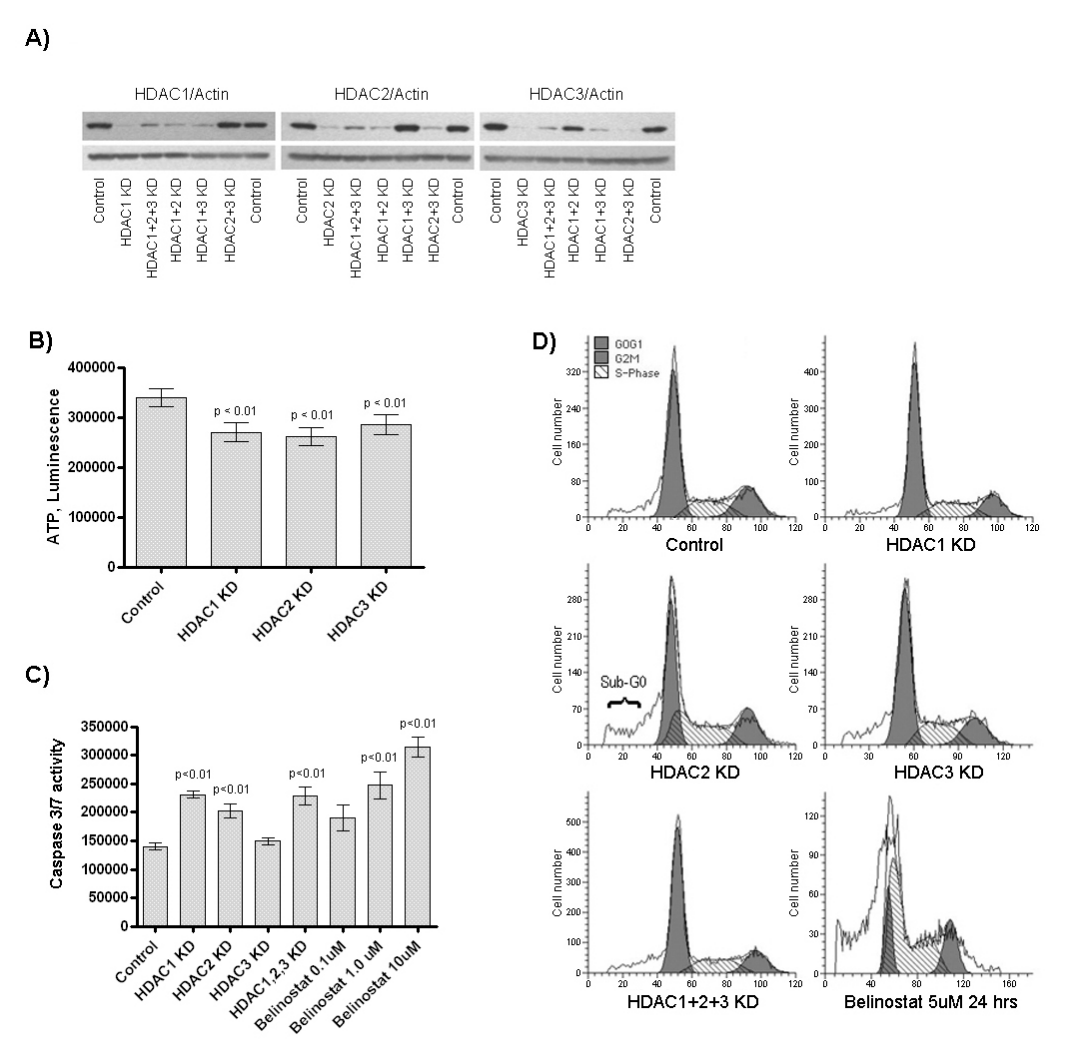
**Effects of HDAC depletion by siRNA in HeLa cells**. **A) **Down-regulation of HDAC1, 2 or 3 protein after treatment with HDAC1, 2 or 3-targeted siRNA respectively, alone or in combination with other siRNAs at 72 hours post-transfection. Top-row is the appropriate antibody (HDAC1, 2 or 3), while the bottom-row is loading control actin; **B) **viability as measured by ATP present in culture. For each HDAC knockdown condition, viability was consistently reduced as compared to scrambled siRNA control, in independent measurements (p < 0.01, n = 24); **C) **The activity of effector caspases 3 and 7 corresponding to apoptotic progression upon HDAC down-regulation or belinostat treatment at increasing concentrations for 24 hours for comparison; **D) **flow cytometric analysis of DNA content as a function of cell number in control or HDAC combination KD at 48 hours post-transfection, or belinostat treatment at 5.0 μM for 24 hours.

Efficient and specific down-regulation of HDAC1, -2 and -3 was obtained in HeLa cells at both protein (Fig. 1A) and mRNA levels (Table 1, bold), by using the siRNA technology. Viability, as measured by metabolically active cells present in culture, was consistently reduced by 20, 23 and 16% following HDAC1, -2 and -3 KD, respectively (Fig. 1B). A similar effect was seen in HCT116 and MCF-7 cells (data not shown). In HDAC1+2 double KD cells, proliferation was reduced by 35% and 25% when compared with single HDAC1 KD and HDAC2 KD cells, respectively (see additional file 4). Apoptotic effector caspase-3/7 activity was significantly increased for HDAC1, -2 and combination KD (1.65-, 1.45- and 1.63-fold respectively, p < 0.01), but not for HDAC3 KD alone. Further, a dose-response of 1.4, 1.8 and 2.3-fold increased apoptosis at 0.1, 1.0 and 10.0 μM at 24 hours is evident for belinostat treatment (Fig. 1C). No indication of cell cycle deregulation was observed for class I HDAC KD in HeLa at 48 hours post-transfection. However, an increase in the subdiploid population corresponding to fragmented cells was observed for especially HDAC2 and to some extent in HDAC3 KD cells, though not for HDAC1 KD cells. In comparison, belinostat treatment showed marked cell cycle alterations and cell debris (Fig. 1D).

**Table 1:** Examples of gene-expression changes in response to HDACi treatment or single class I HDAC enzyme depletion.

Gene symbol	Gene name	Probe nr.	Belinostat	Valproic acid	HDAC1 KD	HDAC2 KD	HDAC3 KD
**Transcription**							
***HDAC1***	**Histone deacetylase 1**	**201209_at**			**0.10**		
***HDAC2***	**Histone deacetylase 2**	**201833_at**				**0.10**	
***HDAC3***	**Histone deacetylase 3**	**216326_s_at**					**0.14**
*JUN*	Jun oncogene (AP-1)	201465_s_at			0.38	0.41	0.42
*SMAD3*	SMAD family member 3	218284_at			0.53	0.49	0.55
*RAF1*	v-raf-1 murine leukemia viral oncogene homolog	201244_s_at			2.88	2.94	2.93
**Metabolism**							
*CTPS*	CTP synthase	202613_at	0.39	0.44			
*TYMS*	Thymidylate synthase	202589_at	0.62	0.43			
*FUCA*	Fucosidase, alpha-L-1, tissue	202838_at	5.86	5.41			
*SAT1*	Spermidine/spermine N1-acetyltransferase 1	210592_s_at	3.81	3.54			
**Cell cycle**							
*CDKN1A*	Cyclin-dependent kinase inhibitor 1A (p21, Cip1)	202284_s_at	4.15	3.16			
*CDC25B*	Cell division cycle 25 homolog B	201853_s_at	0.68	0.58			
*HRASLS3*	HRAS-like suppressor 3	209581_at	5.96	4.70	4.14	2.31	2.11
*CCNB1*	Cyclin B1	214710_s_at	0.55	0.39			
*CCND1*	Cyclin D1	208712_at	0.38				
*CCND2*	Cyclin D2	200951_s_at		2.29			
*CCNE1*	Cyclin E1	213523_at	2.04	1.69			
*CCNG1*	Cyclin G1	208796_s_at			4.39	4.11	3.31
**Apoptosis**							
*FAIM*	Fas apoptotic inhibitory molecule	220643_s_at	0.32	0.17			
*BCL2L11*	BCL2-like 11 (apoptosis facilitator) (BIM)	1555372_at	1.95				
*CASP3*	Caspase 3	202763_at				0.51	0.52
*CASP7*	Caspase 7	207181_s_at			1.86	1.72	1.53
*CASP9*	Caspase 9	203984_s_at			1.57	3.24	1.87
*FAS*	FAS (TNF receptor superfamily, member 6)	216252_x_at			1.55		0.65
**Cell signaling**							
*RASGRP2*	RAS guanyl releasing protein 2	214369_s_at	3.16	6.06			
*MYC*	v-myc myelocytomatosis viral oncogene homolog	202431_s_at	0.43				
*MINA*	MYC induced nuclear antigen	213189_at	0.31	0.40			
*TGFα*	Transforming growth factor, alpha	205016_at		3.24			
*RASGRP1*	RAS guanyl releasing protein 1	205590_at			0.12	0.10	0.25
*CGA*	glycoprotein hormones, alpha polypeptide	204637_at	5.30	4.36	2.66	16.53	6.27
**Miscellaneous**							
*KPNB1*	Importin β	208975_s_at	0.67	0.57			
*SERPINE1*	Serpin peptidase inhibitor, clade E	202627_s_at			0.21	0.23	0.22
*EDN1*	Endothelin 1	218995_s_at			0.45	0.45	0.45
*THBS1*	Thrombospondin 1	201108_s_at			0.30	0.30	0.51
*ADAM19*	ADAM metallopeptidase domain 19	209765_at			0.33		0.37
*PDK4*	Pyruvate dehydrogenase kinase 4	205960_at	4.13	7.34	2.74	3.31	4.38
*ATP10D*	ATPase, Class V, type 10D	213238_at	3.85	3.46	2.40	2.04	2.88

Absolute fold-changes related to untreated or scrambled control for HDACi (VPA or belinostat) or HDAC 1, 2, or 3 KD samples respectively are listed. Blank fields correspond to no significant changes above 1.5-fold. The complete gene list of all genes regulated by 2-fold or more is accessible as supplementary material (Table A).

### HDAC1 knockdown reduces sensitivity to the HDACi belinostat

**Figure 2:**
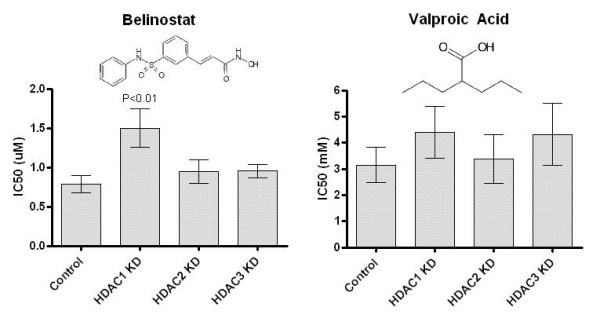
**Mean HDACi IC50 values of mock treated or HDAC deprived HeLa cells**. HDAC1 KD significantly increases the resistance to belinostat on average 1.9-fold (p < 0.01, n = 10), which is not observed for HDAC2 or -3 KD cells. For VPA, no significance was obtained for the 1.4-fold-changes seen in response to both HDAC1 and -3 KD (n = 5). For all figures, standard errors of the mean IC50 value for each condition are shown. The chemical structures for belinostat and VPA are shown over the graphs.

Next, we examined how HeLa cells respond to HDACi treatment following individual class I HDAC enzyme down-regulation (Fig. 2). Interestingly, HDAC1 KD significantly increased IC50 values almost 2-fold towards the hydroxamate belinostat (p < 0.01), which was not seen in response to either HDAC2 or 3 depletion. When examining VPA, no significance was observed for either HDAC KD condition.

### Gene-expression profiles of belinostat and VPA treatment

**Figure 3:**
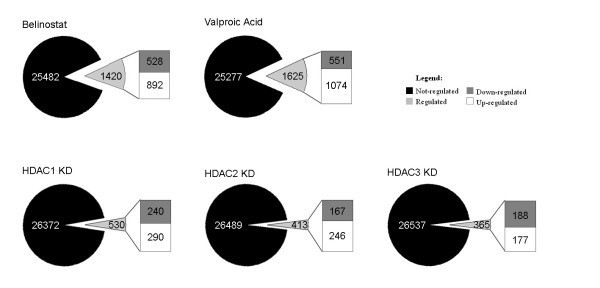
**Significant differentially expressed transcripts**. Found by statistical Limma analysis for each treatment condition vs. their respective control, with an arbitrary 2.0-fold cut-off value. Gene redundancy was taken into account, which left 26902 probes from the chip as total number of genes. The total number of regulated genes, and the proportion of genes up- or down-regulated are shown.

**Figure 4:**
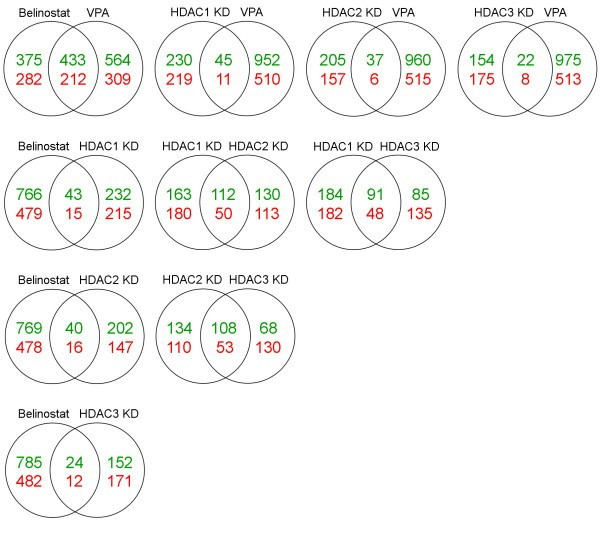
**Overlapping genes between conditions**. Venn diagrams illustrating the distribution of significantly regulated transcripts between each of the 5 conditions, including non-redundant genes at ≥ 2.0-fold expression changes. Up-regulated genes are shown in green, down-regulated in red. The numbers between two conditions illustrate the number of genes regulated in the same direction in both conditions.

Global gene-expression analysis has previously been performed following HDACi treatment regimens primarily in human cell lines [24,25], but only once recently for individual human class I HDAC KD [9]. However, a direct comparison of gene-expression profiles for each has not been reported. To determine first the transcriptional responses to the HDACi used, DNA chip analyses were performed in independent triplicates for each condition; mock treated control, belinostat and VPA treatment in HeLa cells (0.5 μM and 3.0 mM respectively for 24 hours). The doses chosen were close to the IC50 values in HeLa: 0.76 μM and 3.3 mM for belinostat and VPA respectively, and induced histone H3 and H4 hyper-acetylation (data not shown). Differential gene-expression patterns were detected between each experimental condition versus control. Fig. 3 summarizes the number of non-redundant genes significantly deregulated in response to drug treatment, at an arbitrary 2.0 fold-change cut-off value. The number of genes deregulated by belinostat or VPA is 5.3 and 6.0% respectively. Further, a greater proportion of genes are induced by both drugs (63 and 66% respectively). The relationship of differentially expressed genes between belinostat and VPA was illustrated by Venn diagrams (Fig. 4), and demonstrated that approximately 30% of altered genes responded identically between drug treatments.

In the literature, certain genes have been identified whose expression is affected by HDACi treatment [24,25]. For instance, Glaser et al. recognized a common set of 13 core genes whose expression were universally altered in response to various HDACi in multiple cell types [24]. Some of the commonly affected genes are listed in Table 1 for belinostat and VPA treatment, and these include up-regulation of CDKN1A (p21) and FUCA and down-regulation of TYMS, CTPS and KPNB1 (importin β) by both drugs. Hence, this study confirms a subset of 5 of the 13 core genes besides other known HDACi target genes. Comprehensive gene-lists of all conditions at above 2-fold changes are accessible (see additional file 2). Data were validated by qRT-PCR analysis on 9 selected genes on RNA samples used in microarray analysis plus independent ones, and an overall good correlation to the microarray data was observed (see additional file 1).

### Gene-expression profiles of class I HDAC depletion

Further, we conducted a genome-wide analysis of the transcriptional response to siRNA-mediated depletion of three class I HDACs in HeLa cells. As for HDACi samples, DNA chip analyses were carried out in independent triplicates for each condition; scrambled siRNA control, HDAC1, -2 and -3 KD at 48 hours post-transfection. Differential gene-expression patterns between each knockdown condition and scrambled control were identified by statistical analysis. Efficient KD was confirmed by the microarray data, as each HDAC isoform was specifically down-regulated 7–10 fold (Table 1, bold). The proportion of non-redundant significant transcripts affected by the down-regulation of each HDAC enzyme at 2.0-fold change or more is in the range 1.4–2.0% (shown in Fig. 3), and highest for HDAC1 KD. As for HDACi drugs, a slight overweight of transcripts was induced for HDAC1 and -2 KD samples. In contrast, HDAC3 KD was the only condition not showing this pattern. The proportion of genes with identical expression between KD conditions was in the order of 19–27% (Fig. 4), with HDAC1 KD displaying the least overlap with the other two KD conditions. Further, the individual HDAC isoenzyme targeted by its specific siRNA was the most down-regulated gene in each respective sample, and siRNA targeting one HDAC did not affect expression levels of other class I HDACs. The complete gene lists for all conditions ≥ 2.0-fold changes are accessible (see additional file 2). Also, data were validated by qRT-PCR analysis on 6 selected genes on RNA samples used in microarray analysis plus independent ones, which overall had good correlations to the microarray data (see additional file 1). We further addressed the effect of a combined HDAC1+2 KD by analyzing mRNA expression of three genes (HRASLS3, CCND1 and THBS) found to be affected by each individual HDAC KD. For the CCND1 and THBS genes, KD of either HDAC1 or -2 reduced expression by approximately 50–75% compared to control; an effect not observed in double HDAC1+2 KD (Supplemental figure 2B). For the expression of the HRASLS3 gene, an increase of approximately 50% is seen with single HDAC1 or -2 KD, which increases to approximately 200% in HDAC1+2 double KD cells. Together, these data indicate a degree of redundancy of the HDAC1 and -2 proteins.

### Cell-specific effects of individual class I HDAC depletion

A previous report by Senese et al. [9] studied the transcriptional effect of HDAC1, 2 and 3 KD in the human U2OS osteosarcoma cell line, by microarray analysis. In a direct comparison study, we find very little overlap between the results obtained in the present study and the data recently published by Senese et al. [9]. As discussed below, this apparent discrepancy can be attributed to both methodological and biological differences between the two studies. First, while the experimental design in the Senese study relies on 2 technical replicates of a biological pool on each array, which is then scanned twice (before and after amplification), in our study, we have chosen the more traditional approach with 3 independent biological replicates for each experimental condition and array. Because the biological variation between individual samples most often is much larger than the assay variation [41], it appears more efficient to perform single arrays on independent biological samples, rather than replicate arrays on a limited number of samples.

Second, we had the opportunity to reanalyze the data (CEL files kindly provided by Dr. Susanna Chiocca, European Institute of Oncology, Milan, Italy) from the Senese study. In our hands, the number of significantly regulated genes following HDAC siRNA inhibition is much lower than reported in the original study (Additional file 5, slide 19). This disparity is probably due to the stringent filtering criteria applied in our analysis, where we require the absolute difference between differentially expressed genes to be 50 or more, as otherwise, the risk of obtaining false positive results due to genes that are close to the background level, is high. Finally, we have made a direct comparison between our knockdown experiments and those performed by Senese et al. From this analysis, it is evident that the overriding differences observed between the two studies are due to cell type specific effects. Both the PCA plot based on 1965 probes that vary between experimental conditions (Additional file5 , slide 29) and the unsupervised hierarchical clustering (Additional file 5, slide 30) imply that the two data sets are clearly separated, and only have a few genes in common when looking at each specific HDAC. That it is indeed the biology that is the main variable between the two studies, and not a technical artifact, is supported by looking at the expression levels of the three HDACs, which are all among the most down-regulated genes, when their corresponding siRNA is transfected into the cells. Thus, we do see HDAC specific effects common for the two studies, but the predominant effects are due to the diverse biology of the two systems. Interestingly, HDAC3 is only number 122 among the most down-regulated genes in the HDAC3 siRNA experiment in the Senese et al. paper. This can be explained by the already low HDAC3 expression in the control cells, so going from a low basal expression to a lower siRNA inhibited expression level will not give as large a fold-change as when looking at a gene with a high basal expression. In summary, the low degree of concordance between the two studies can be attributed mainly to cell specific differences, which further emphasizes the importance and impact of the model system chosen for a particular study (see additonal file 6).

### Dissimilar transcriptional profiles between individual HDAC depletion and HDACi treatment

We thus wanted to directly compare individual class I HDAC enzyme knockdown with the treatment of two structurally distinct classes of HDACi in the same cell line, as this has not been done previously in human cells. Approximately three times more genes are deregulated by enzymatic HDACi treatment than by individual class I HDAC depletion (Fig. 3). Between treatment types, a slightly greater proportion of genes are in common for the two structurally distinct HDACi, than are in common for KD of individual HDACs from the same class. However, the biggest difference occurs when comparing single class I HDAC KD with HDACi treatment, as merely 1.6–3.4% of altered genes overlap between these conditions, depending on enzyme and drug assayed (Fig. 4). Thus, only a small proportion of genes respond identically when either knocking down single class I HDAC enzymes or inhibiting multiple HDACs enzymatically. A complete list of genes whose expression is altered in the same direction between one or more HDAC KDs and one or both HDACi treatments are listed (see additional file 3), including genes with ≥ 2.0-fold changes only. This comprises merely 109 genes altered in response to both types of HDAC inhibition, and includes genes involved in processes such as transcriptional regulation, gametogenesis and development, metabolism and intracellular trafficking. Of the 109 genes, 7 are up-regulated simultaneously in all 5 conditions; 3 with unknown function besides HRASLS3 involved in negative cell cycle regulation, CGA involved in cell-cell signaling, PDK4 involved in carbohydrate metabolism and the ion transporter ATP10D.

## Discussion

Targeting cancer through epigenetic control mechanisms is an area of growing interest. While HDACi show promise in clinical trials, the contribution of each HDAC isoenzyme in the anti-proliferative response of HDACi is unknown. In the present study, we directly compared gene-expression profiles between the two modes of HDAC inhibition; single class I HDAC protein depletion by siRNA and enzymatic HDACi treatment in a human cancer cell line. It is recognized, that HDACs function in multi-protein complexes and their depletion therefore might have a dissimilar outcome to HDACi treatment [27], however this has not been directly addressed previously.

The reduced viability that we observe upon individual HDAC1, -2 and -3 knockdown has been published on class I HDAC KD in cancer cells, especially via proliferation for HDAC1 and -3, and via apoptosis for HDAC2 [6,8,11,19]. We also detected an increased subdiploid population of HDAC2 and less for -3 KD cells, whereas caspase activity was increased for HDAC1 and -2 KD cells. Thus, mediators of apoptosis following HDAC KD might be dissimilar between the isoforms examined. Caspase 3 as a mediator of apoptosis in HDAC1 KD cells was recently reported [9], as was an increased subdiploid population for HDAC2 KD [10,19] and HDAC3 KD [6,19,28], but not in HDAC1 KD [19], thus supporting our results. Further, we found no major alterations in cell cycle distribution in response to class I HDAC KD, which is in agreement with other reports [10,11,28]. To conclude, class I HDAC KD causes a reduction in viability and an increase in apoptosis, however at much lower levels than detected for HDACi treatment, as this is not transferred to alterations in cell cycle distributions.

Published data suggest a wide range in the proportion of genes deregulated in response to HDACi treatment; between 1–22%. This depends on factors such as class of compound, dosage, incubation time and choice of cell line [24,25,29-31]. Hence, our data on belinostat and VPA in HeLa cells are within this broad range. Between belinostat and VPA, the shared proportion of genes of 30% probably correspond to the overlapping functions as HDAC inhibitors as both drugs affect some typical HDACi-induced genes, whereas differences are attributed to structural dissimilarities, HDAC class specificity, and non-HDACi functions of VPA. Other reports comparing the transcriptional response of different HDACi compounds find approximately 45% similarities between trichostatin A (TSA) and either tributyrate or vorinostat and 77% identical genes between tributyrate and vorinostat treatment, when examining three cancer cell lines [25], while vorinostat and depsipeptide had very similar responses in one cell line, especially in the first hours of treatment [31]. Further, of the limited 'core' set of 13 genes universally affected by HDACi treatment [24], 5 were reproduced by both drugs in this study. In response to single class I HDAC down-regulation, none of these 13 genes were altered, however the expression of a considerable amount of genes were altered that included genes involved in proliferation, apoptosis or adhesion. For HDAC1, this corresponds to data on *C. elegans *in which 2.2% were altered by ≥ 1.8-fold [32], albeit lower than the 7% observed in HDAC1 knockout of untransformed murine embryonic stem cells at 2-fold or more [33], probably due to the complete abrogation of HDAC1 in this system. HDACi treatment and individual HDAC KD have been shown to cause both up- and down-regulation of multiple gene targets [5,32,33]. The knockdown of class I HDAC enzymes in this report showed that near equal proportions of genes were induced as were repressed by HDAC KD, with a slight overweight of induced genes for HDAC1 and -2 KD and a slight overweight of down-regulated genes for HDAC3, possibly separating this isoform as mainly a transcriptional activator. As HDAC1 and -2 reside in the same co-repressor complexes, the disruption of these might have more similar outcomes. Moreover, we found that HDAC1 KD altered the greatest number of genes, and hence might affect gene transcription to a larger extent than HDAC2 and -3. Between the three KD conditions, we found most genes (73–80%) to be uniquely deregulated upon individual HDAC KD, with HDAC1 having the least degree of overlap. This suggests distinctive transcriptional targets for HDAC enzymes from the same class, and could thus provide the basis for discrete functions between class I HDACs [11]. In comparison with genes affected by HDAC1, -2 or -3 KD by siRNA in human U2OS cells in a recent study [9], the majority were not reproduced herein, and generally point to cell-line specific responses to HDAC depletion. This emphasizes the importance of comparing HDAC KD with HDACi treatment in the same cell line.

Finally, we compared individual KD of class I HDAC members with two dissimilar HDACi compounds at near-IC50 doses. At the treatment regimens chosen, three times more genes were deregulated by HDACi treatment than by individual class I HDAC KD. As these drugs target multiple HDACs, this is not unexpected. The overlap of genes between HDACi treatments and between individual HDAC KD was in a similar range; 20–30%. When looking into the genes whose expression overlapped between HDACi treatment and individual KD of the target HDACs of these compounds, a surprisingly low degree of similarity was observed, namely less than 4% of regulated genes. The reason for the low degree of overlap could have several explanations. First, some degree of redundancy might occur after individual HDAC KD. A prior study in Drosophila showed an overlapping proportion of 20% (469 of 2347 genes regulated in total) between DHDAC1 KD and TSA treatment, each for 5 days post-treatment. However, reducing TSA treatment to 6 hours also reduced the overlap to 4.5% (52/1151), thus differences in experimental set-up probably account for a large variation in these numbers. For DHDAC3 KD, the overlap with TSA treatment was 2%, and the authors conclude that especially DHDAC1 affected gene expression in a similar manner to TSA [34]. The closer resemblance between DHDAC1 and TSA profiles might be because Drosophila has fewer HDAC enzymes and DHDAC1 is orthologous to both human HDAC1 and -2. Second, depleting HDAC levels most likely interferes with the multi-protein complexes in which they reside in a different manner than by enzymatic drug inhibition of HDAC, causing differential cellular responses. It has previously been shown in Drosophila, that DHDAC1 deficiency and point mutations had dissimilar phenotypic outcomes, the latter presumably by altering HDAC complexes rather than disrupting them [35]. Third, we showed that the transcriptional profile obtained by individual HDAC KD is not simply elaborated by inhibiting multiple HDAC enzymes but altered altogether, and thus other mechanisms might contribute to the HDACi effects other than targeting individual class I HDAC enzymes. These differences might explain why single class I HDAC KD is not as toxic as pan-inhibitory HDACi treatment and fails to produce identical phenotypic effects, despite the probable effects of HDACi mainly via class I HDAC enzymes.

## Methods

### Cell culture and drugs

Human cervix cancer cells HeLa, CCL-2 (American Type Culture Collection, Manassas, VA) and mammary cancer cells MCF-7 (HTB-22) were propagated in DMEM + glutamax media supplemented with penicillin and streptomycin and 10% FBS; the colon cancer cell line HCT116 (CCL-247) was maintained in RPMI-1640 media supplemented with glutamine, penicillin, streptomycin and 10% FBS (Invitrogen, Carlsbad, CA). All were grown in a humidified atmosphere of 5% CO_2 _at 37°C and passaged twice a week. Belinostat was synthesized as described in recent patent applications (International publication number US 6,888,027), and valproic acid was purchased from Sigma-Aldrich (St. Louis, MO). Drugs were dissolved in sterile water, aliquoted and stored at -20°C until use.

### Transfection of siRNA

Pre-designed targeting siRNA SmartPOOL was purchased from Dharmacon (Lafayette, CO) (non-targeting siRNA D-001206-13, HDAC1 M-003493-02, HDAC2 M-003495-01, HDAC3 M-003496-00). Cells were plated in 6-well plates, 250,000/well in complete media and incubated overnight prior to aspiration of media and replacement with OPTI-MEM (Invitrogen) with a final concentration of 50 nM siRNA complexed with oligofectamine (Invitrogen). Cells were incubated 4–6 hours before addition of 1 ml growth medium with 20% FCS.

### RNA extraction

Cells were plated in 6-well plates at 250,000/well and left overnight before the transfection procedure, or replaced with fresh media with drug at 0.5 μM for belinostat or 3.0 mM for VPA. 48 hours post-transfection and 24 hours after drug treatment, total RNA was extracted with Trizol according to the manufacturers protocol (Invitrogen). For microarray samples, 5 of the 6 wells pr condition were pooled to minimize well-to-well variation. The 6^th ^well was lysed directly in SDS sample buffer (Invitrogen), and used for protein analysis.

### DNA microarray analysis

RNA integrity was quality checked on the Agilent 2100 Bioanalyser (Agilent Technologies, Santa Clara, CA), then processed and hybridized onto Affymetrix arrays according to the manufacturer's protocol. Briefly, 5 μg RNA pr sample was used to to generate biotin-labeled antisense cRNA. After fragmentation, the labeled cRNA samples were hybridized to Affymetrix HG-U133 Plus 2.0 arrays (Affymetrix, Santa Clara, CA), washed and stained with phycoerytrin conjugated streptavidin, and finally scanned in the Affymetrix GeneArray^® ^scanner to generate fluorescent images, as described in the Affymetrix GeneChip^® ^protocol. All statistical analyses, including pre-processing of data was carried out in R, version 2.3 (R development core team. R: A language and Environment for Statistical Computing; ). DNA chips were checked for quality assurance parameters such as visual image inspection, replicate scatter plots and RNA degradation plots, before normalization for mean overall expression using the gcrma package (GC robust multiarray algorithm) [36]. Agglomerative hierachical clustering showed that the biological replicates clustered together as expected (data not shown). The statistical linear model based method of Limma (linear models for microarray data package) [37] was found to be most sensitive at identifying genes with differential expression between control and each condition. Raw p-values were adjusted for multiple testing using the Benjamini & Hochberg method [38] to reduce the number of false positives, and a 5% significance threshold applied. Comparisons of gene lists between conditions was performed using VennMapper [39].

### Quantitative reverse transcription polymerase chain reaction

RNA was reverse transcribed by RT-PCR using 1 volume diluted RNA (100 ng/μl) and 1 volume 2× RT-master mix (High Capacity cDNA archive kit, Applied Biosystems, Foster City, CA) exposed to 25°C 10 minutes and 37°C 2 hours. Samples were analyzed for gene-expression levels by qRT-PCR, performed on ABI PRISM™ 7500 Sequence Detection System (Applied Biosystems). Experiments were done in triplicate by mixing 1 μL probe, 10 μL 2× Taqman master mix and 9 μL cDNA diluted 1:50, and subjecting samples to 40 cycles of amplification (15 seconds denaturation at 95°C, 1 minute annealing and elongation at 60°C), using GAPDH (HDAC KD) or β-Actin (drug treatments) as endogenous controls. All pre-validated FAM-labeled probes were purchased from Applied Biosystems. Subsequent data analysis was performed using DART-PCR version 1.0 [40].

### Western Blotting

Cells were lysed directly in SDS sample buffer (Invitrogen) and electrophoretically separated and transferred to nitrocellulose paper, following the manufacturer's instructions (Invitrogen pre-cast Novex gels). Blots were blocked in 10% non-fat skimmed milk, incubated overnight with primary antibody, washed in Tris-buffered saline with Tween-20 and visualized by HRP-conjugated secondary antibodies (1:2,000) and ECL plus reagent (GE Healthcare, Chalfont St. Giles, Buckinghamshire, UK). Antibodies uses were: rabbit anti-HDAC1 and -3 (1:1,000, Cell Signaling Technology, Danvers, MA), mouse anti-HDAC2 (1:10,000, Abcam, Cambridge, UK), rabbit anti-Actin (1:5,000 Sigma-Aldrich).

### CellTiter-Glo Assay

Scrambled control and HDAC KD cells were plated in triplicate at 10,000/well in 96-well format 24 hours post-transfection. Cells were incubated 48 hours, without drug for viability measurements, and within expected belinostat toxicity limits for determination of IC50 values. Cells were lysed directly with CellTiter-Glo luminescent viability assay (Promega, Madison, WI), and luminescence proportional to ATP present hence metabolically active cells was measured (HTS 7000 plus Bioassay reader, PerkinElmer Life and Analytical Sciences, Waltham, MA). Data were normalized to the scrambled control, and IC50 values determined in Prism 4 by generation of a sigmoidal dose-response curve with variable slope (GraphPad Software, San Diego, CA). Significant changes in mean viability or IC50 values in the four groups (control, HDAC1, -2 or -3 KD) were calculated by ANOVA one-way analysis of variance repeated measures test and Dunnett's multiple comparisons test in Prism (GraphPad Software).

### Caspase-Glo 3/7 assay

Cells were plated at 10^4^/well, in quadroplicates for each HDAC KD condition and control 48 hours post-transfection, or in triplicates for drug treatments. Plates were incubated for 24 hours prior to direct lysis by Caspase-Glo 3/7 reagent (Promega), and luminescence reading according to caspase 3/7 activity.

### Cell cycle analysis

Transfected cells were incubated for 48 hours before analysis. For drug treatments, cells were plated in 6-well format 2.5 × 10^5^/well, incubated overnight, treated with drug for 24 hours and processed as follows. Cells were permeabilized by incubation in ice-cold 70% ethanol, rehydrated in PBS supplemented with Tween-20 and FBS, RNAse treated and DNA stained with propidium iodide. Cells were analyzed using a FACSCalibur instrument and the CellQuest software (BD Biosciences, Mountain View, CA).

## List of abbreviations

HDAC: histone deacetylase; HDACi: histone deacetylase inhibitor; IC50: half maximal inhibitory concentration; KD: knockdown; qRT-PCR: quantitative reverse transcriptase polymerase chain reaction; siRNA: small interfering ribonucleic acid; TSA: trichostatin A; VPA: valproic acid.

## Declaration of competing interests

Marielle Dejligbjerg was partly sponsored by a grant from the Danish Ministry of Science, Technology and Innovation, and the biotechnology company TopoTarget A/S. Morten Grauslund is a shareholder and full-time employee at TopoTarget A/S. Thomas Litman is an employee of Exiqon A/S. Laura Collins is a previous employee of TopoTarget A/S. Xiaozhong Qian and Michael Jeffers are previous employees of CuraGen Corp. Henri Lichenstein is a shareholder and full-time employee at CuraGen Corp. Peter B Jensen is CEO of and shareholder in TopoTarget A/S. Maxwell Sehested is CSO of and shareholder in TopoTarget A/S.

## Authors' contributions

MD designed and performed siRNA transfections, qRT-PCR, western blotting, viability assays and flow cytometry analyses, helped with secondary microarray analysis and drafted the manuscript. MG participated in the design of the study and drafting of the manuscript. TL and LC performed microarray data analysis. XQ assisted in siRNA transfections, western blotting and flow cytometry analyses. MJ, HL, PBJ and MS conceived of the study, participated in its design and coordination, and provided intellectual discussions and ideas regarding the content of manuscript. All participating authors read and approved the final manuscript.

## Supplementary Material

Additional file 1 *Additional file A*: Supplemental Figure 1. Validation of gene-expression changes from microarray analysis, by qRT-PCR. For each gene, data from microarray analysis and corresponding values from qRT-PCR analysis are included. Validation was shown for both one of the same RNA purifications as were used for microarray analysis ("A"), and independent ones in HeLa ("B"). A) Genes affected by HDACi treatment, B) Genes affected by HDAC KD Click here for file

Additional file 2 *Additional file B*: Supplemental Table A: Complete gene lists at ≥ 2-fold changes. Complete gene lists of significantly altered genes (5%) in all five conditions: HDAC 1, 2 or 3 KD, VPA or belinostat treatments, at 2-fold changes or more. Click here for file

Additional file 3 *Additional file C*: Supplemental Table B: Transcriptional overlap between conditions. The overlapping proportion of genes regulated by one or more HDACi treatments and by one or more class I HDAC depletion, by at least 2-fold. Thus, these genes respond identically to both types of HDAC inhibition. Click here for file

Additional file 4 *Additional file D*: Supplemental Figure 2. Effect of HDAC1, 2 and 1+2 depletion using the siRNA technology in HeLa cells. A). Effect on cell proliferation by measuring intracellular ATP levels. B) Effect on gene expression by measuring mRNA levels of selected genes by qRT-PCR. Click here for file

Additional file 5 *Additional file E*: Detailed comparison of microarray data from this study to the Senese et al. study [9]. The Power Point file contains 40 slides showing how the analysis was carried out and the results obtained herein Click here for file

Additional file 6 *Additional file F*: Supplemental Table C: Combined analysis of the Senese and Dejligbjerg data. The Excel table allows direct comparison and sorting of the genes that were more than 1.5-fold differentially expressed following HDAC knockdown in the two studies. Click here for file
