# Supplementary material for: Differential effects of class I isoform histone deacetylase depletion and enzymatic inhibition by belinostat or valproic acid in HeLa cells
Source: Mol Cancer. 2008 Sep 12;7:70. doi: 10.1186/1476-4598-7-70 (PMC2553797; doi:10.1186/1476-4598-7-70)

# Supplemental figure 1

Validation of gene expression changes from microarray analysis, by qRT-PCR. For each gene, data from microarray analysis and corresponding values from qRT-PCR analysis are included. Validation was shown for both one of the same RNA purifications as were used for microarray analysis (“A”), and independent ones in HeLa (“B”). **A)** Genes affected by HDACi treatment, **B)** Genes affected by HDAC KD.


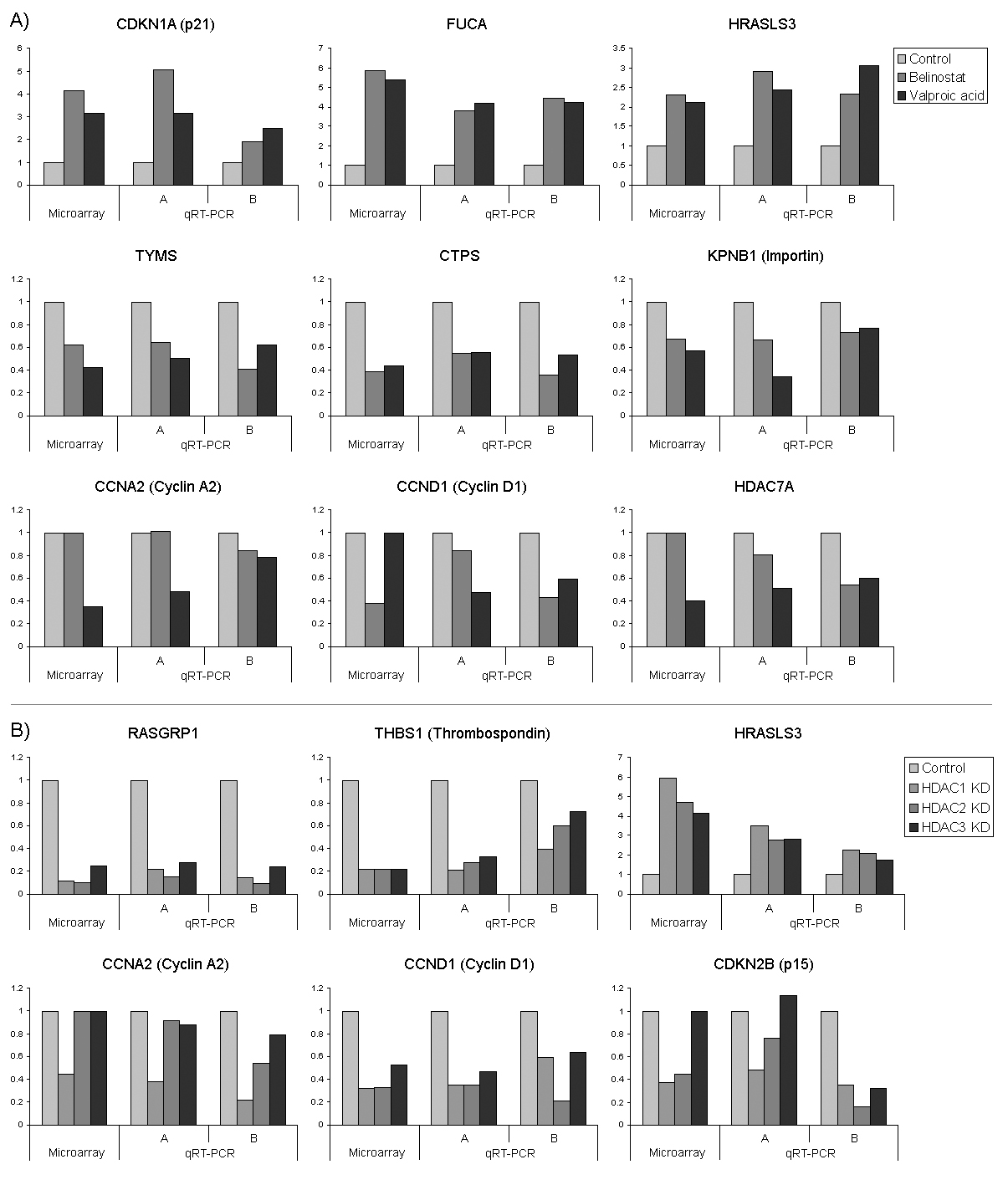

Supplement: Additional file 1 — Additional file A: Supplemental Figure 1. Validation of gene-expression changes from microarray analysis, by qRT-PCR. For each gene, data from microarray analysis and corresponding values from qRT-PCR analysis are included. Validation was shown for both one of the same RNA purifications as were used for microarray analysis ("A"), and independent ones in HeLa ("B"). A) Genes affected by HDACi treatment, B) Genes affected by HDAC KD [file 1476-4598-7-70-S1.doc]
