# Supplementary material for: Differential effects of class I isoform histone deacetylase depletion and enzymatic inhibition by belinostat or valproic acid in HeLa cells
Source: Mol Cancer. 2008 Sep 12;7:70. doi: 10.1186/1476-4598-7-70 (PMC2553797; doi:10.1186/1476-4598-7-70)

**Additional file D**

Supplemental Figure 2. Downregulation of HDAC1, 2 and 1+2 combined in HeLa cells after treatment with HDAC1 and -2 targeted siRNA alone or in combination at 72 hours post-transfection. A) Cell viability as measured by ATP presence in the culture, B) validation of the expression measured by qRT-PCR analysis.


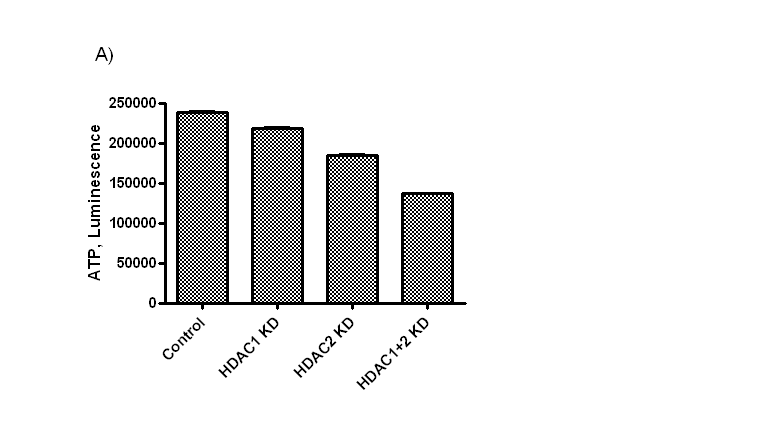


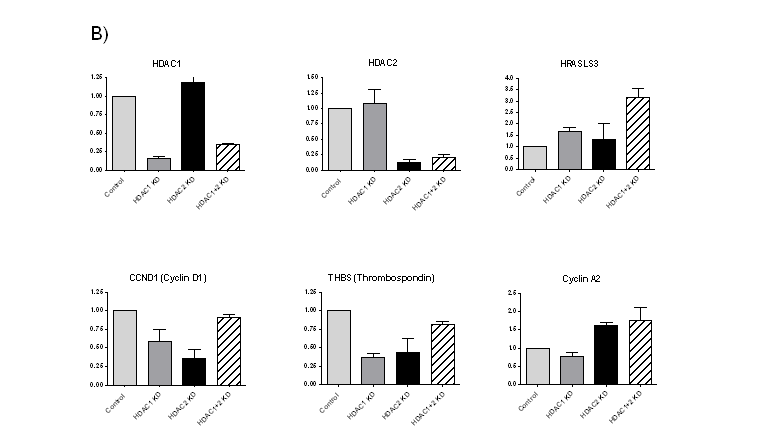

Supplement: Additional file 4 — Additional file D: Supplemental Figure 2. Effect of HDAC1, 2 and 1+2 depletion using the siRNA technology in HeLa cells. A). Effect on cell proliferation by measuring intracellular ATP levels. B) Effect on gene expression by measuring mRNA levels of selected genes by qRT-PCR. [file 1476-4598-7-70-S4.doc]
