# Supplementary material for: Differential effects of class I isoform histone deacetylase depletion and enzymatic inhibition by belinostat or valproic acid in HeLa cells
Source: Mol Cancer. 2008 Sep 12;7:70. doi: 10.1186/1476-4598-7-70 (PMC2553797; doi:10.1186/1476-4598-7-70)
Supplement: Additional file 5 — Additional file E: Detailed comparison of microarray data from this study to the Senese et al. study [9]. The Power Point file contains 40 slides showing how the analysis was carried out and the results obtained herein [file 1476-4598-7-70-S5.ppt]

## Slide 1
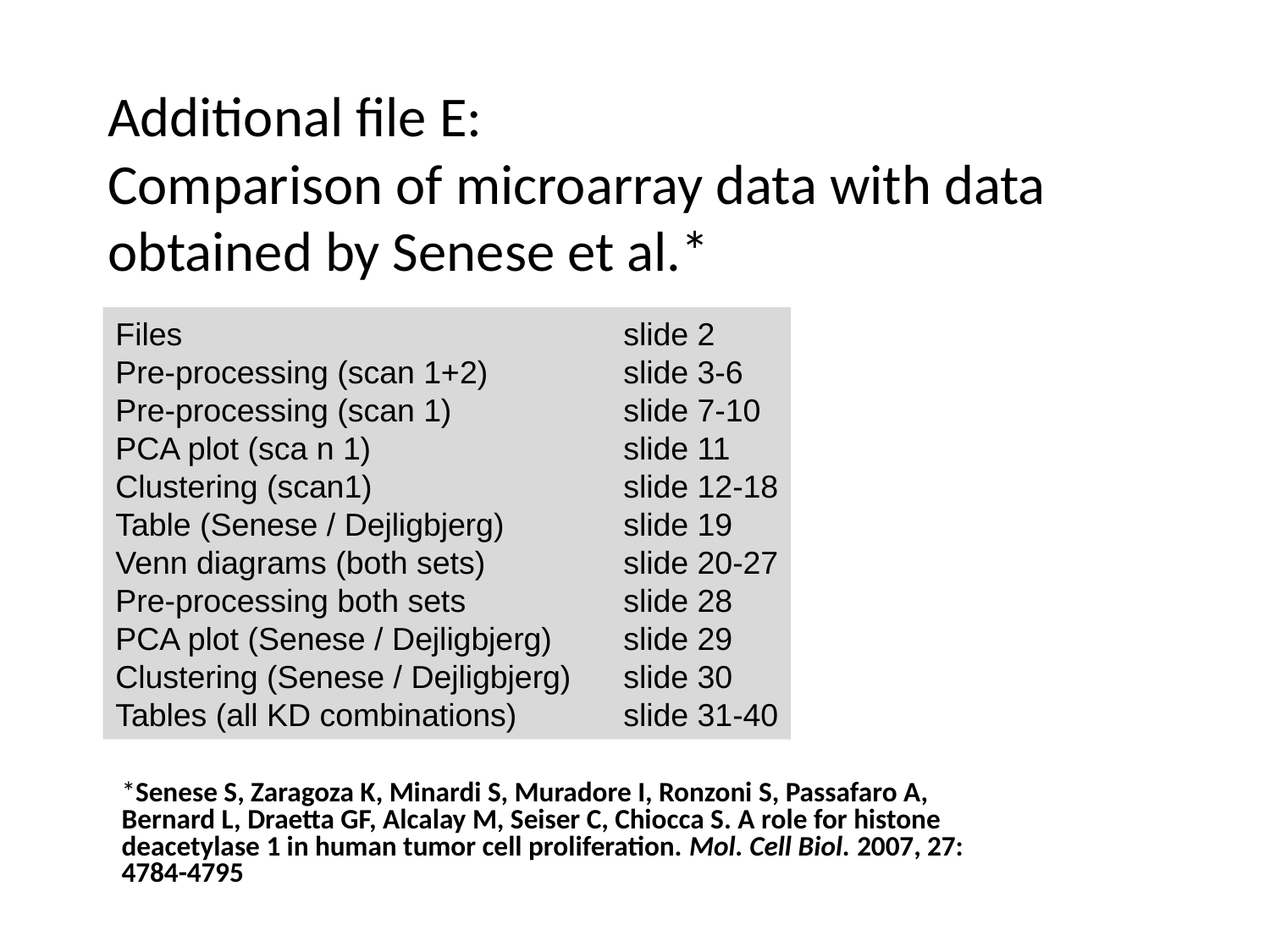

# Additional file E: Comparison of microarray data with data obtained by Senese et al.*
Files				slide 2
Pre-processing (scan 1+2)		slide 3-6
Pre-processing (scan 1)		slide 7-10
PCA plot (sca n 1)		slide 11
Clustering (scan1)		slide 12-18
Table (Senese / Dejligbjerg)	slide 19
Venn diagrams (both sets)		slide 20-27
Pre-processing both sets		slide 28
PCA plot (Senese / Dejligbjerg)	slide 29
Clustering (Senese / Dejligbjerg)	slide 30
Tables (all KD combinations)	slide 31-40
*Senese S, Zaragoza K, Minardi S, Muradore I, Ronzoni S, Passafaro A, Bernard L, Draetta GF, Alcalay M, Seiser C, Chiocca S. A role for histone deacetylase 1 in human tumor cell proliferation. Mol. Cell Biol. 2007, 27: 4784-4795

## Slide 2
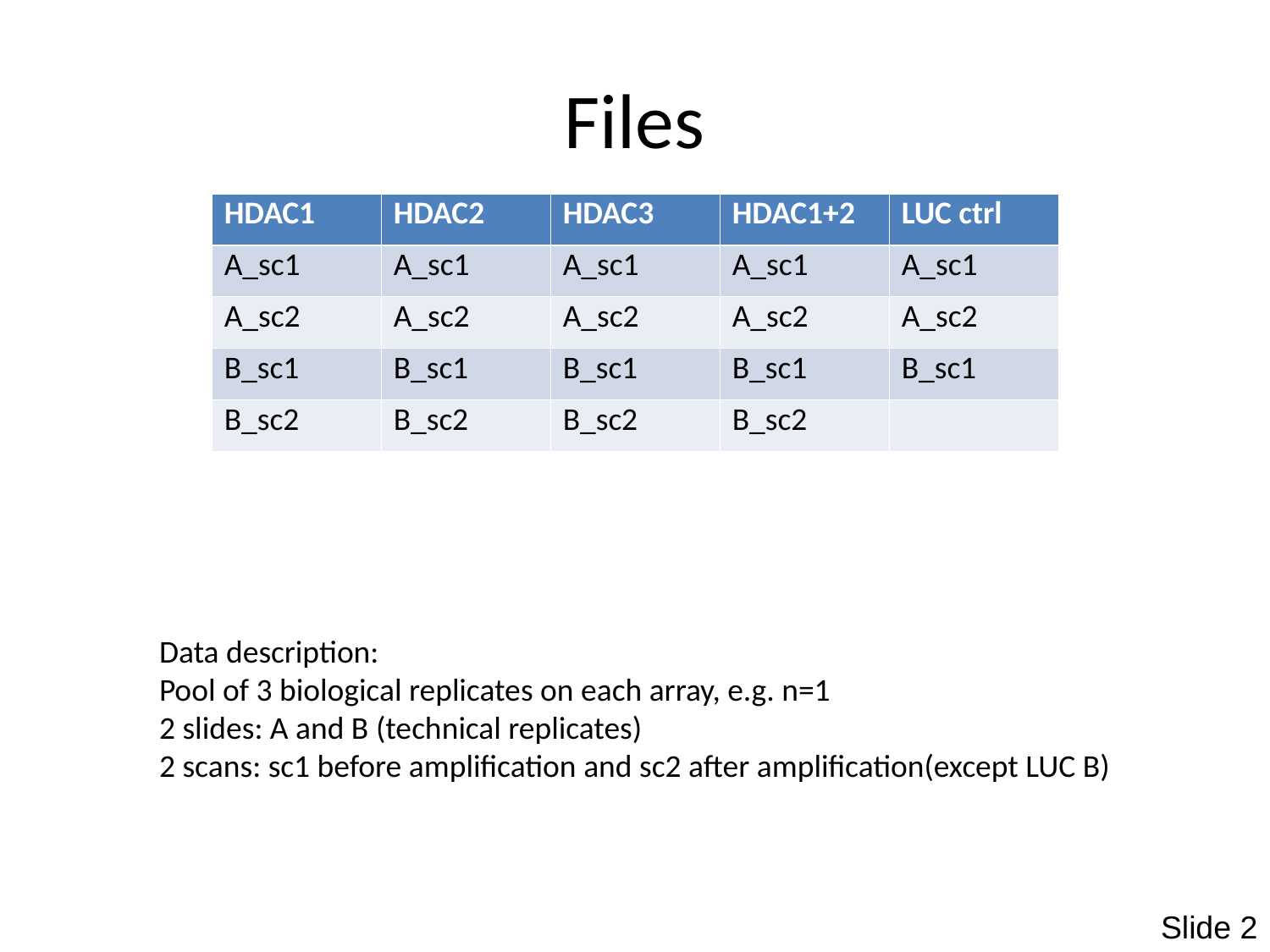

# Files
| HDAC1 | HDAC2 | HDAC3 | HDAC1+2 | LUC ctrl |
| --- | --- | --- | --- | --- |
| A\_sc1 | A\_sc1 | A\_sc1 | A\_sc1 | A\_sc1 |
| A\_sc2 | A\_sc2 | A\_sc2 | A\_sc2 | A\_sc2 |
| B\_sc1 | B\_sc1 | B\_sc1 | B\_sc1 | B\_sc1 |
| B\_sc2 | B\_sc2 | B\_sc2 | B\_sc2 | |
Data description:
Pool of 3 biological replicates on each array, e.g. n=1
2 slides: A and B (technical replicates)
2 scans: sc1 before amplification and sc2 after amplification(except LUC B)
Slide <number>

## Slide 3
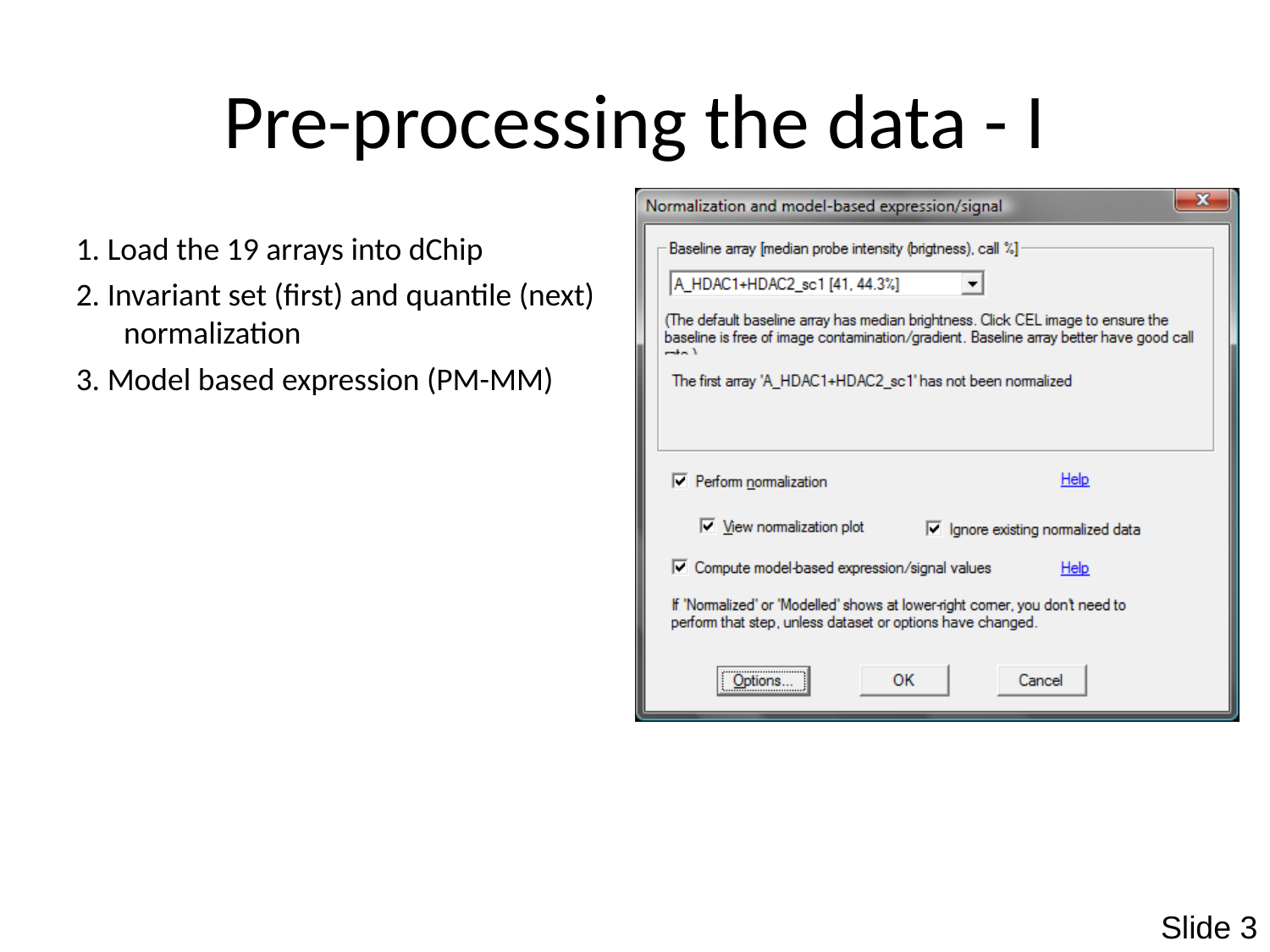

# Pre-processing the data - I
1. Load the 19 arrays into dChip
2. Invariant set (first) and quantile (next)
normalization
3. Model based expression (PM-MM)
Slide <number>

## Slide 4
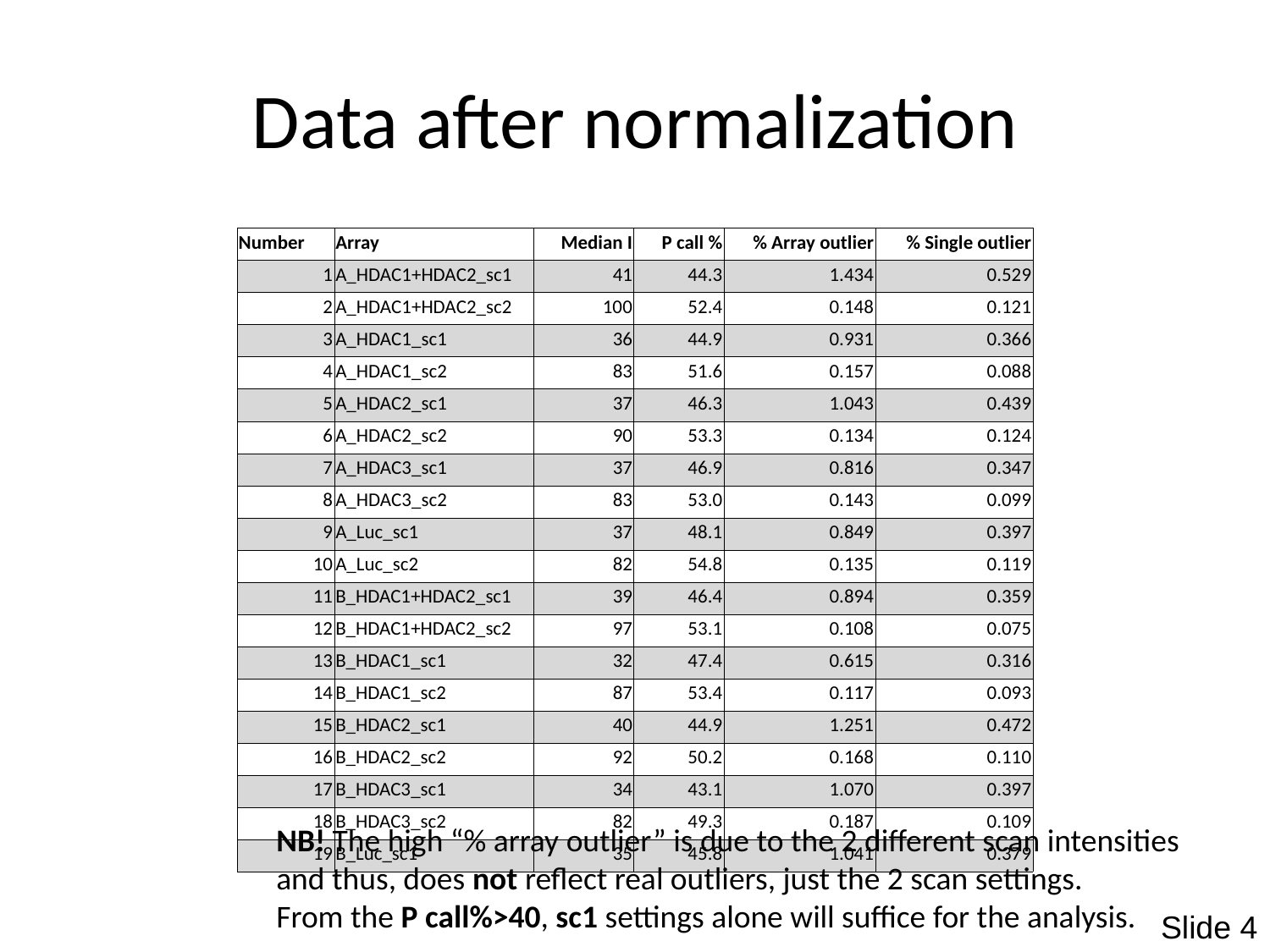

# Data after normalization
| Number | Array | Median I | P call % | % Array outlier | % Single outlier |
| --- | --- | --- | --- | --- | --- |
| 1 | A\_HDAC1+HDAC2\_sc1 | 41 | 44.3 | 1.434 | 0.529 |
| 2 | A\_HDAC1+HDAC2\_sc2 | 100 | 52.4 | 0.148 | 0.121 |
| 3 | A\_HDAC1\_sc1 | 36 | 44.9 | 0.931 | 0.366 |
| 4 | A\_HDAC1\_sc2 | 83 | 51.6 | 0.157 | 0.088 |
| 5 | A\_HDAC2\_sc1 | 37 | 46.3 | 1.043 | 0.439 |
| 6 | A\_HDAC2\_sc2 | 90 | 53.3 | 0.134 | 0.124 |
| 7 | A\_HDAC3\_sc1 | 37 | 46.9 | 0.816 | 0.347 |
| 8 | A\_HDAC3\_sc2 | 83 | 53.0 | 0.143 | 0.099 |
| 9 | A\_Luc\_sc1 | 37 | 48.1 | 0.849 | 0.397 |
| 10 | A\_Luc\_sc2 | 82 | 54.8 | 0.135 | 0.119 |
| 11 | B\_HDAC1+HDAC2\_sc1 | 39 | 46.4 | 0.894 | 0.359 |
| 12 | B\_HDAC1+HDAC2\_sc2 | 97 | 53.1 | 0.108 | 0.075 |
| 13 | B\_HDAC1\_sc1 | 32 | 47.4 | 0.615 | 0.316 |
| 14 | B\_HDAC1\_sc2 | 87 | 53.4 | 0.117 | 0.093 |
| 15 | B\_HDAC2\_sc1 | 40 | 44.9 | 1.251 | 0.472 |
| 16 | B\_HDAC2\_sc2 | 92 | 50.2 | 0.168 | 0.110 |
| 17 | B\_HDAC3\_sc1 | 34 | 43.1 | 1.070 | 0.397 |
| 18 | B\_HDAC3\_sc2 | 82 | 49.3 | 0.187 | 0.109 |
| 19 | B\_Luc\_sc1 | 35 | 45.8 | 1.041 | 0.379 |
NB! The high “% array outlier” is due to the 2 different scan intensities
and thus, does not reflect real outliers, just the 2 scan settings.
From the P call%>40, sc1 settings alone will suffice for the analysis.
Slide <number>

## Slide 5
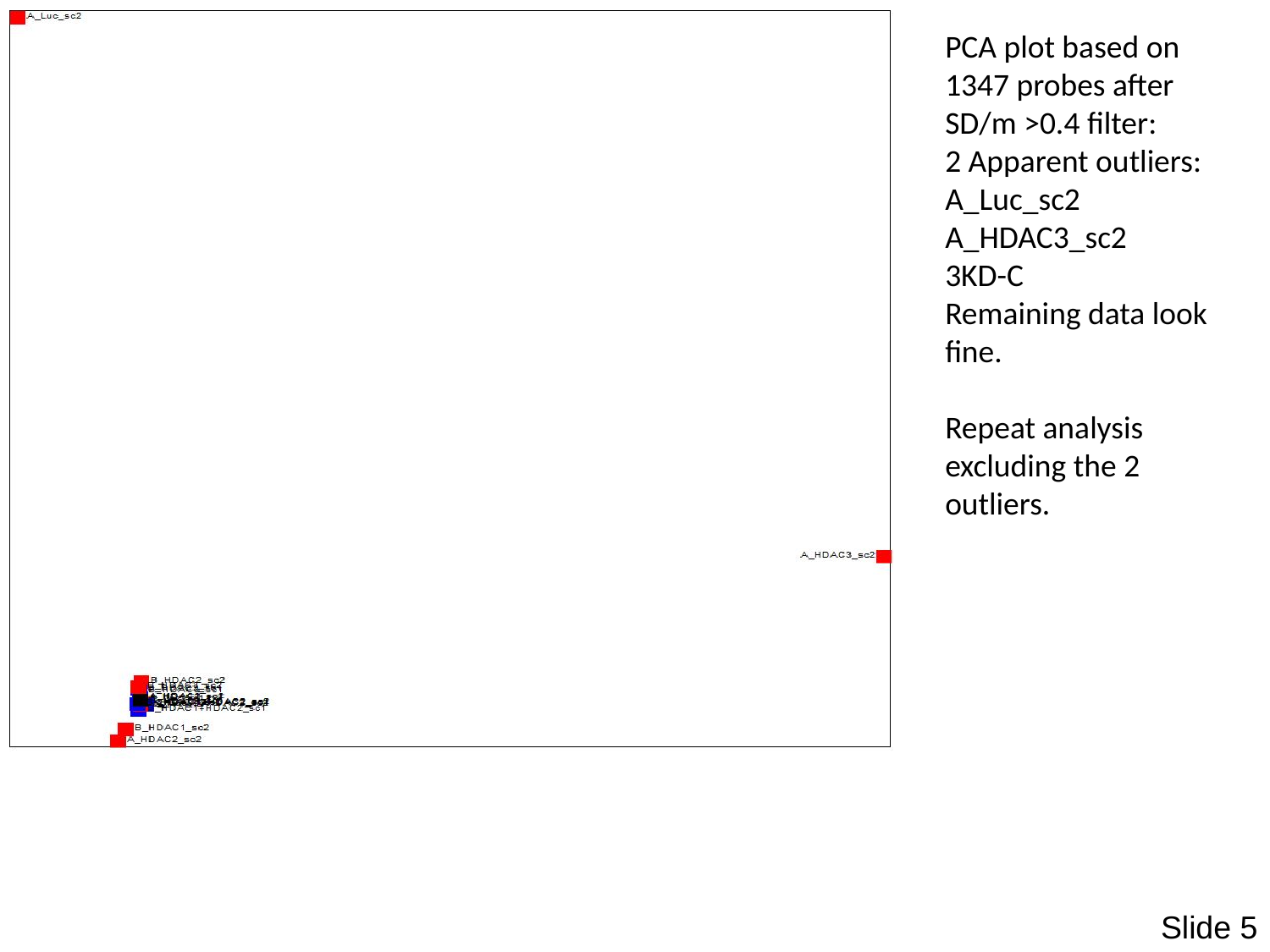

PCA plot based on 1347 probes after
SD/m >0.4 filter:
2 Apparent outliers:
A_Luc_sc2
A_HDAC3_sc2
3KD-C
Remaining data look fine.
Repeat analysis excluding the 2 outliers.
Slide <number>

## Slide 6
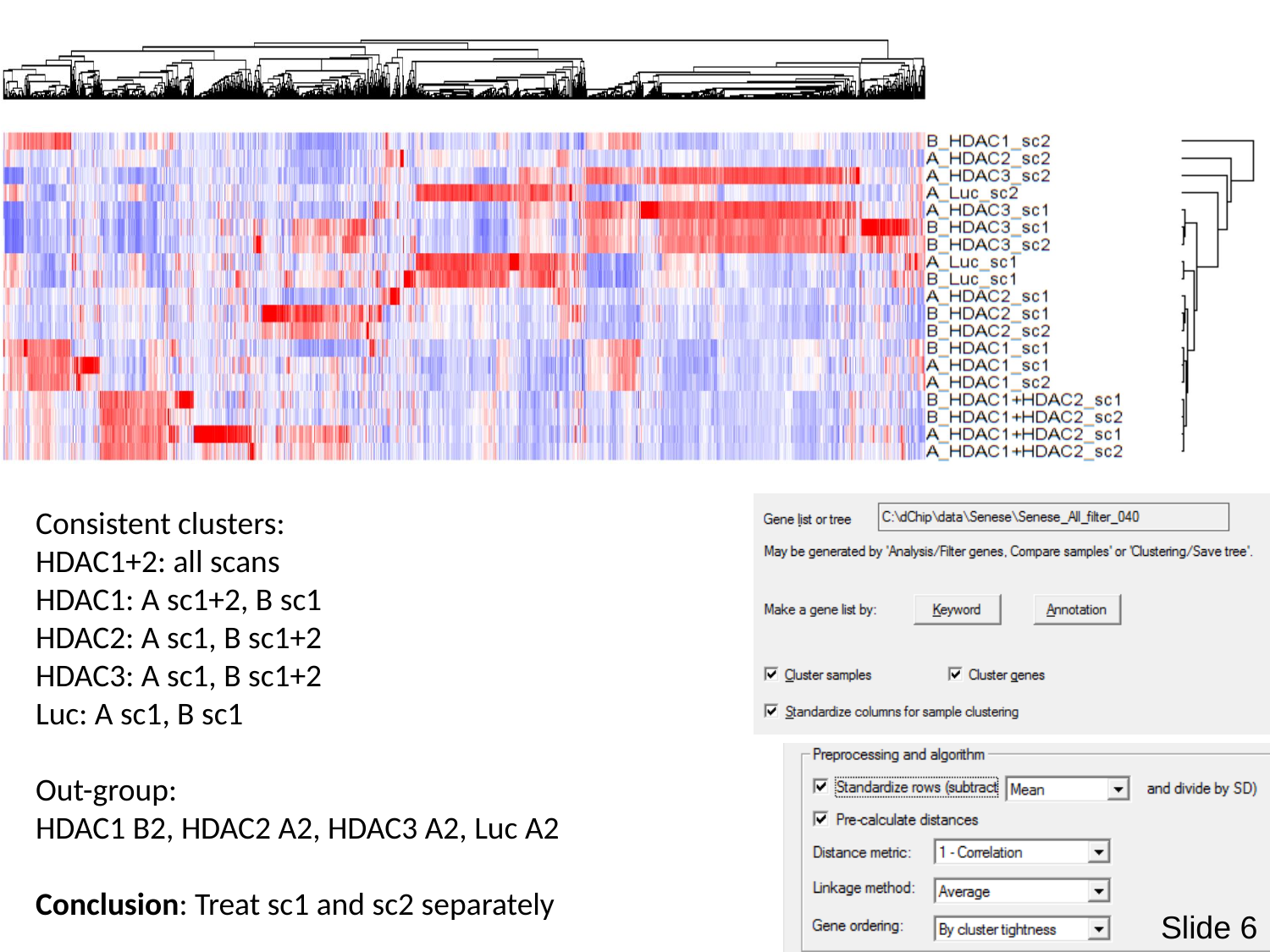

Consistent clusters:
HDAC1+2: all scans
HDAC1: A sc1+2, B sc1
HDAC2: A sc1, B sc1+2
HDAC3: A sc1, B sc1+2
Luc: A sc1, B sc1
Out-group:
HDAC1 B2, HDAC2 A2, HDAC3 A2, Luc A2
Conclusion: Treat sc1 and sc2 separately
Slide <number>

## Slide 7
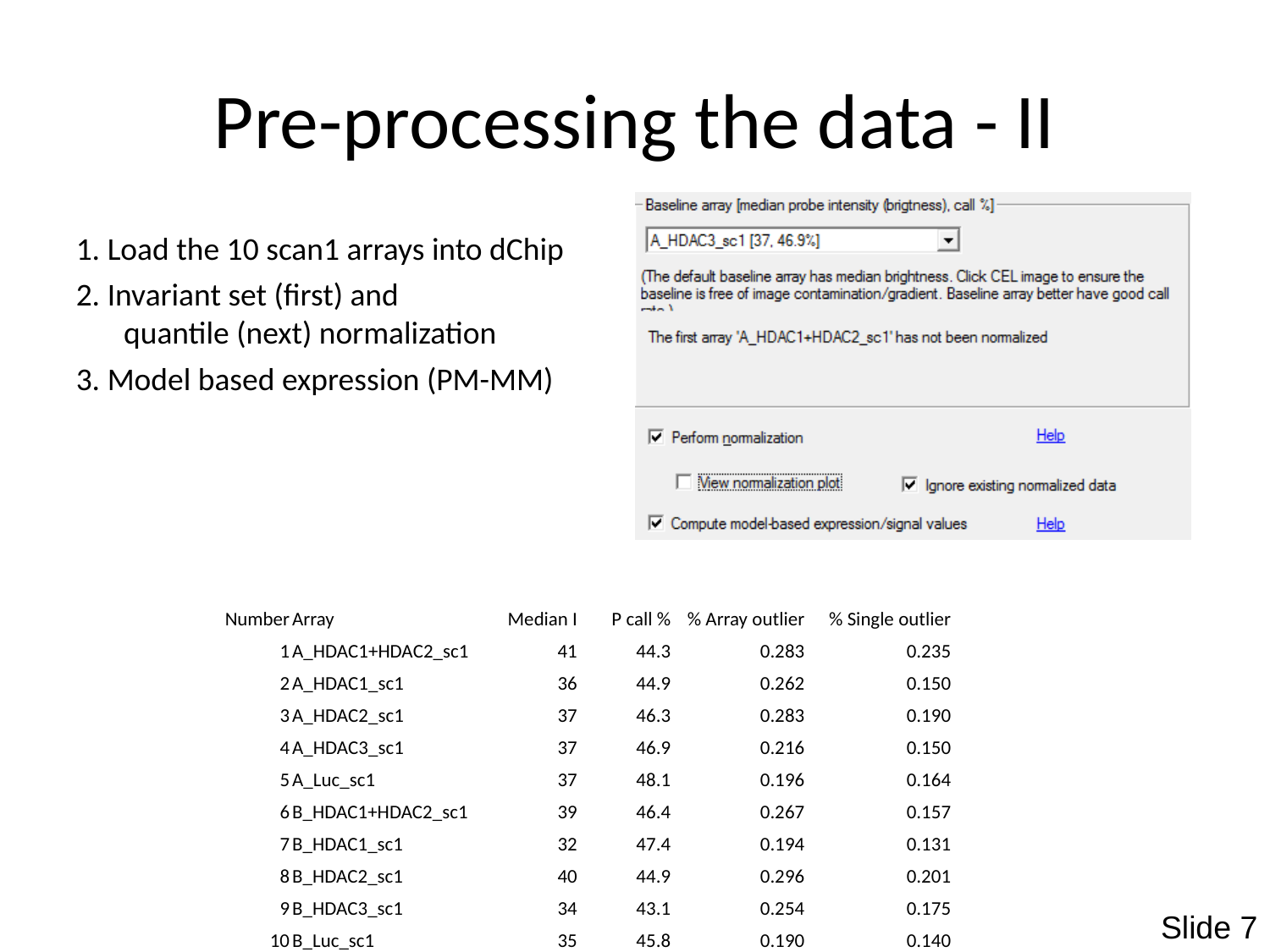

# Pre-processing the data - II
1. Load the 10 scan1 arrays into dChip
2. Invariant set (first) and
quantile (next) normalization
3. Model based expression (PM-MM)
| Number | Array | Median I | P call % | % Array outlier | % Single outlier |
| --- | --- | --- | --- | --- | --- |
| 1 | A\_HDAC1+HDAC2\_sc1 | 41 | 44.3 | 0.283 | 0.235 |
| 2 | A\_HDAC1\_sc1 | 36 | 44.9 | 0.262 | 0.150 |
| 3 | A\_HDAC2\_sc1 | 37 | 46.3 | 0.283 | 0.190 |
| 4 | A\_HDAC3\_sc1 | 37 | 46.9 | 0.216 | 0.150 |
| 5 | A\_Luc\_sc1 | 37 | 48.1 | 0.196 | 0.164 |
| 6 | B\_HDAC1+HDAC2\_sc1 | 39 | 46.4 | 0.267 | 0.157 |
| 7 | B\_HDAC1\_sc1 | 32 | 47.4 | 0.194 | 0.131 |
| 8 | B\_HDAC2\_sc1 | 40 | 44.9 | 0.296 | 0.201 |
| 9 | B\_HDAC3\_sc1 | 34 | 43.1 | 0.254 | 0.175 |
| 10 | B\_Luc\_sc1 | 35 | 45.8 | 0.190 | 0.140 |
Slide <number>

## Slide 8
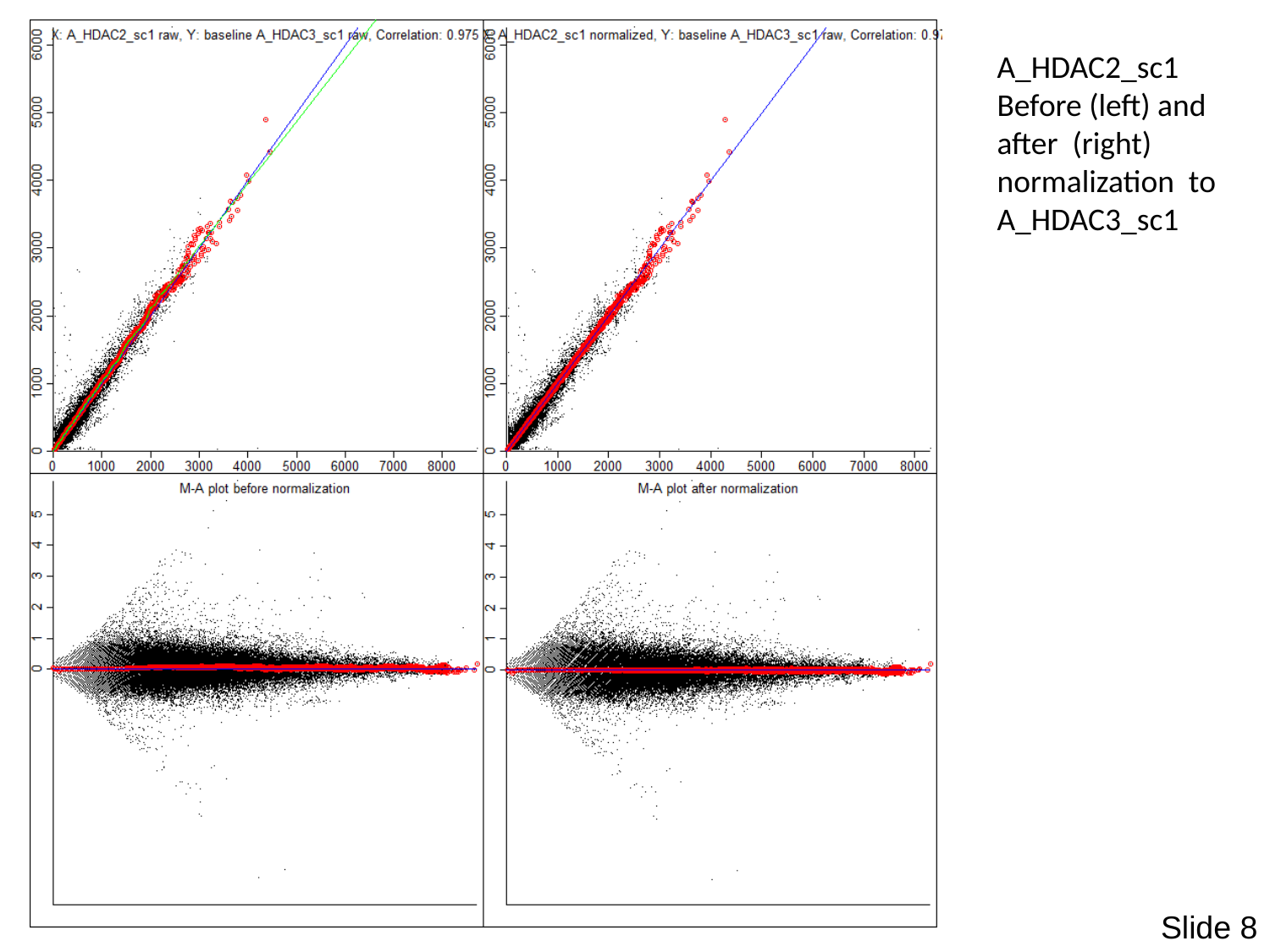

A_HDAC2_sc1
Before (left) and
after (right)
normalization to
A_HDAC3_sc1
Slide <number>

## Slide 9
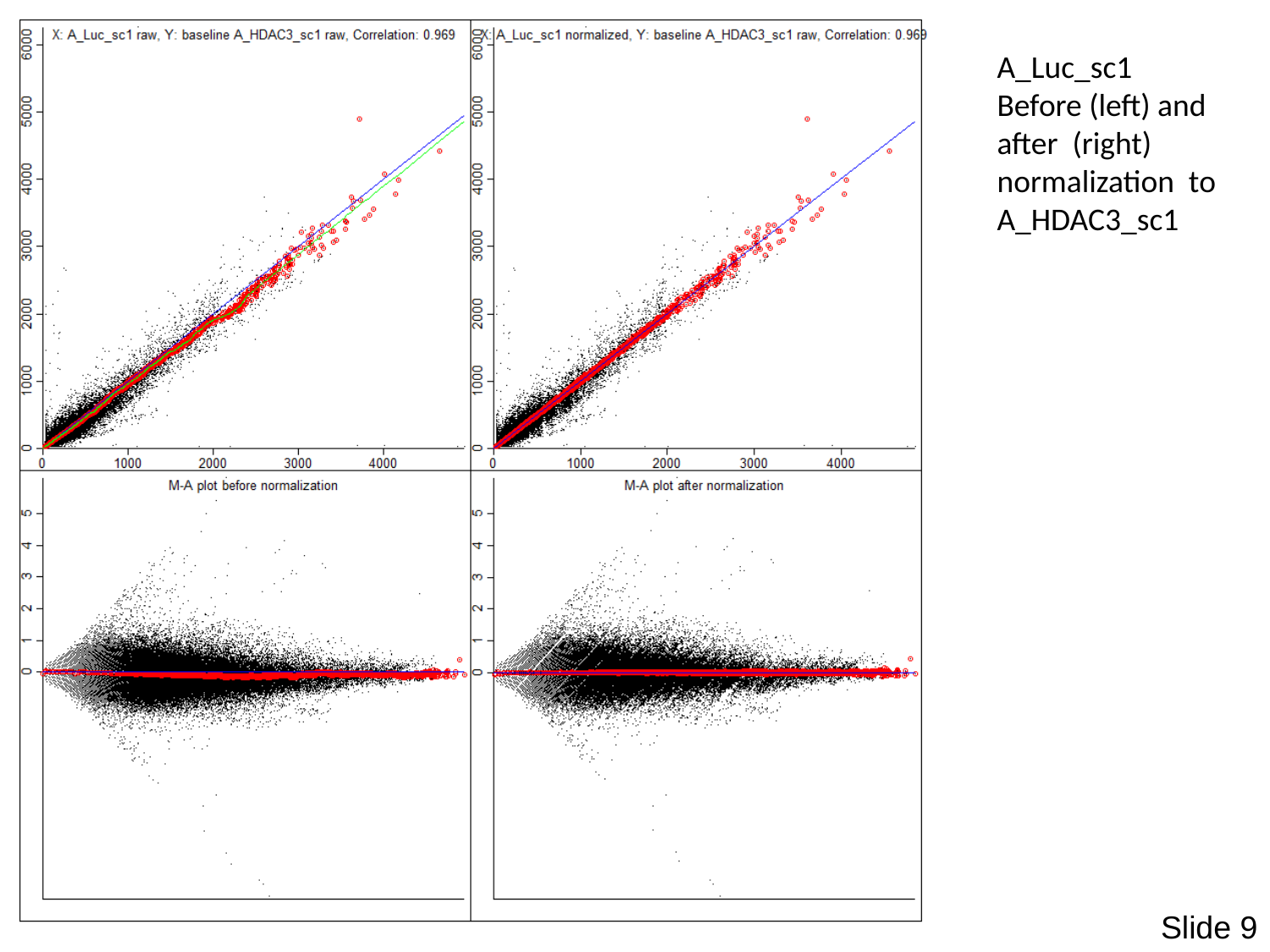

A_Luc_sc1
Before (left) and
after (right)
normalization to
A_HDAC3_sc1
Slide <number>

## Slide 10
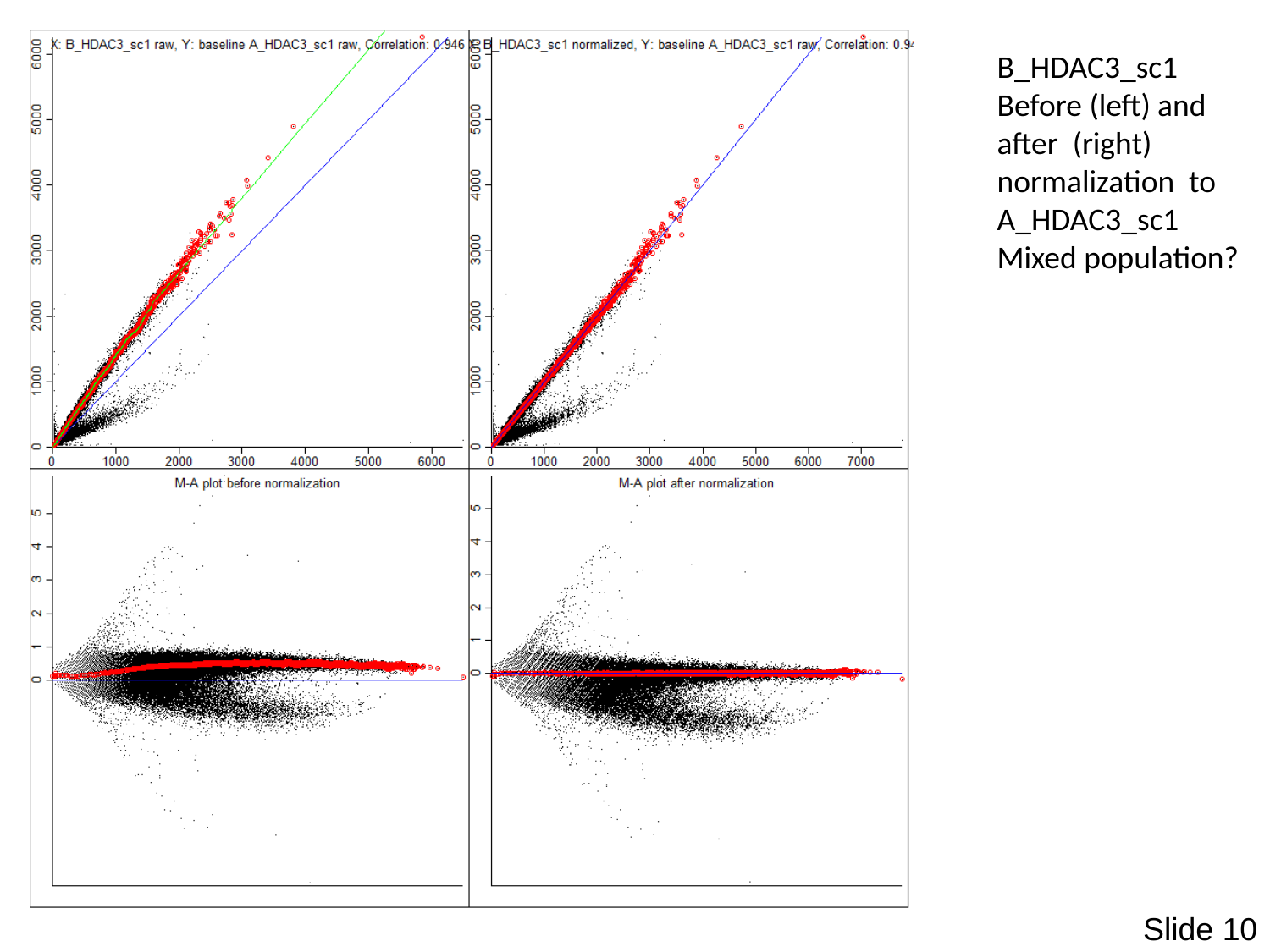

B_HDAC3_sc1
Before (left) and
after (right)
normalization to
A_HDAC3_sc1
Mixed population?
Slide <number>

## Slide 11
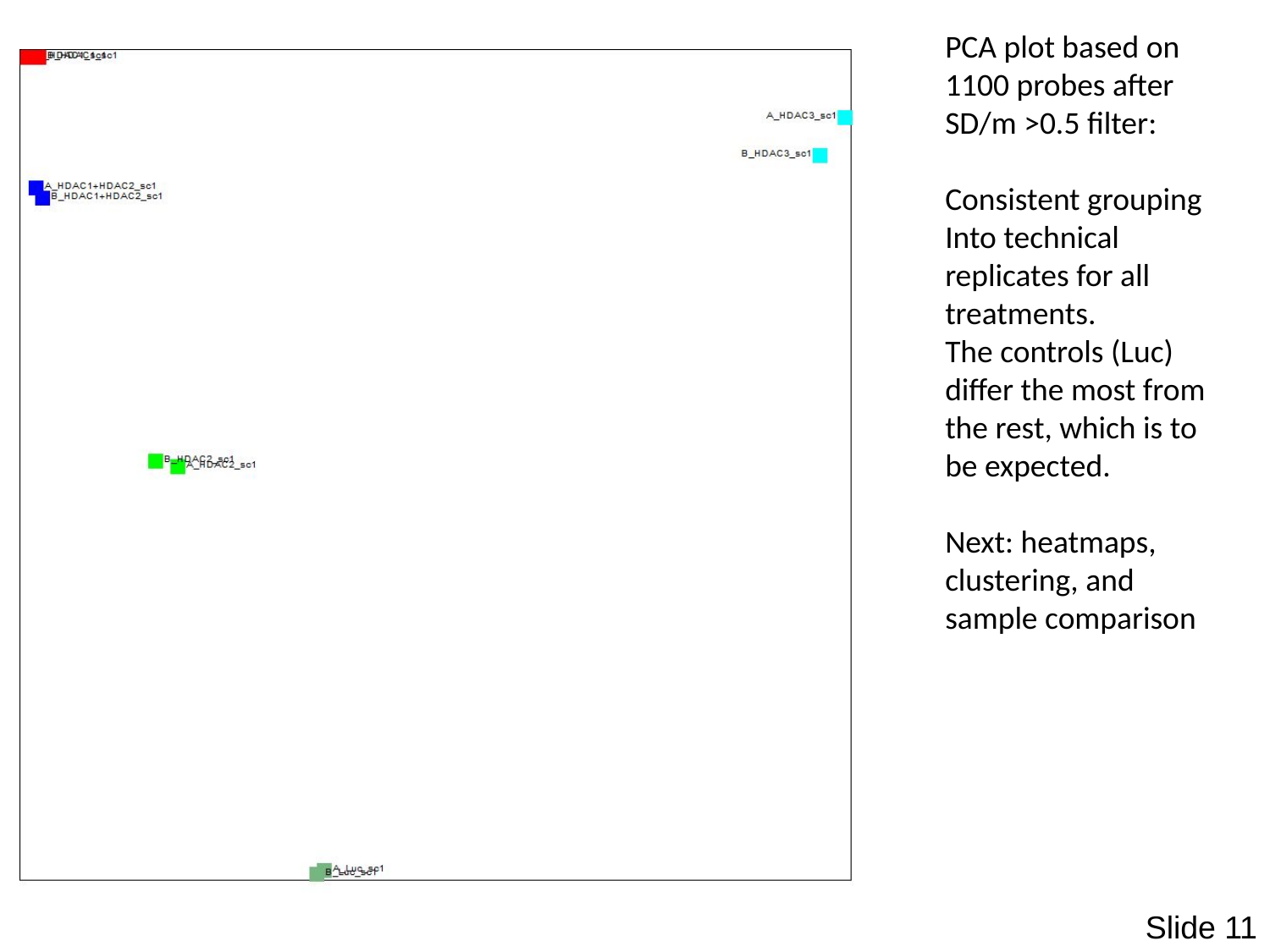

PCA plot based on 1100 probes after
SD/m >0.5 filter:
Consistent grouping
Into technical replicates for all treatments.
The controls (Luc) differ the most from the rest, which is to be expected.
Next: heatmaps, clustering, and sample comparison
Slide <number>

## Slide 12
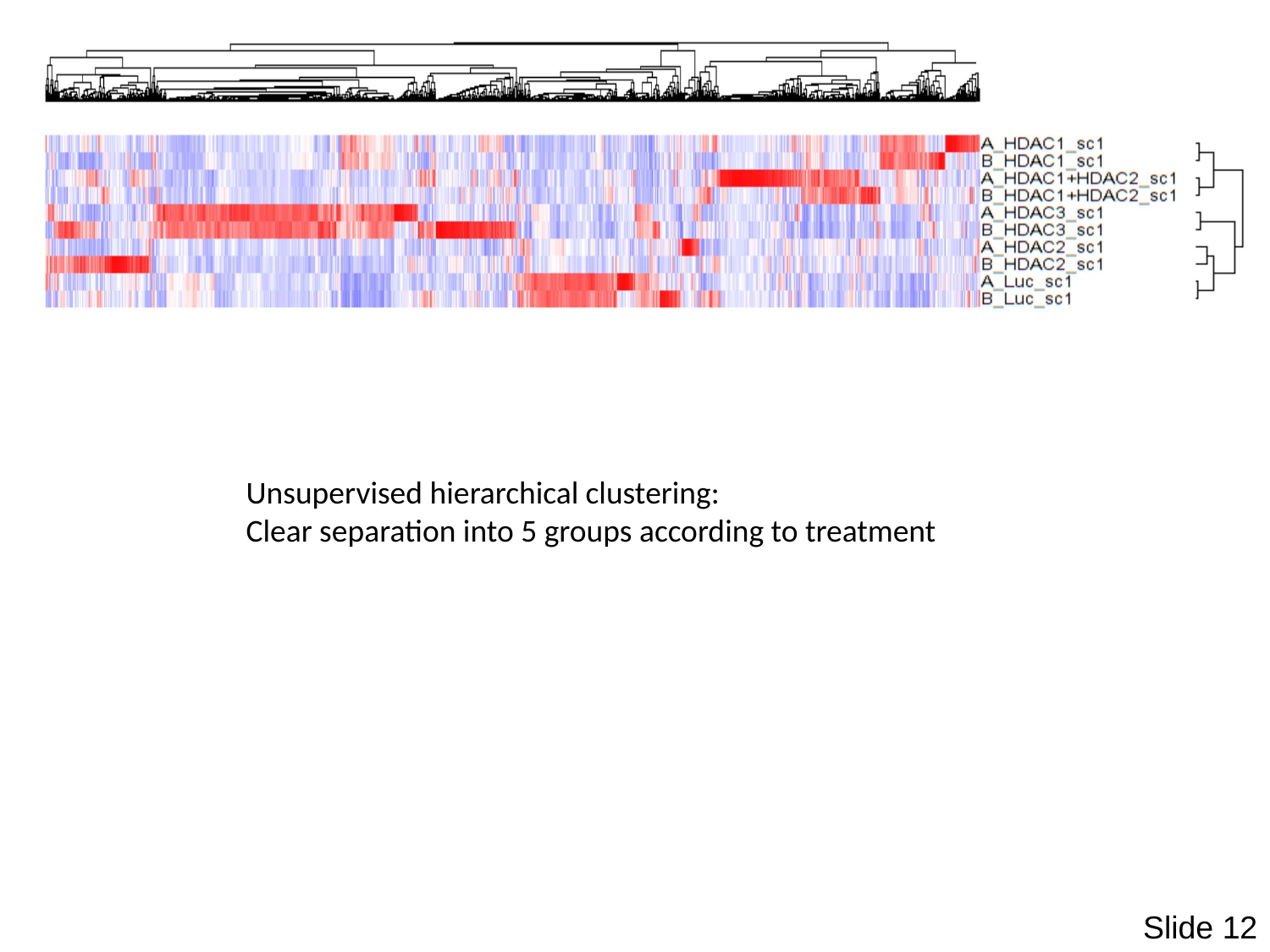

Unsupervised hierarchical clustering:
Clear separation into 5 groups according to treatment
Slide <number>

## Slide 13
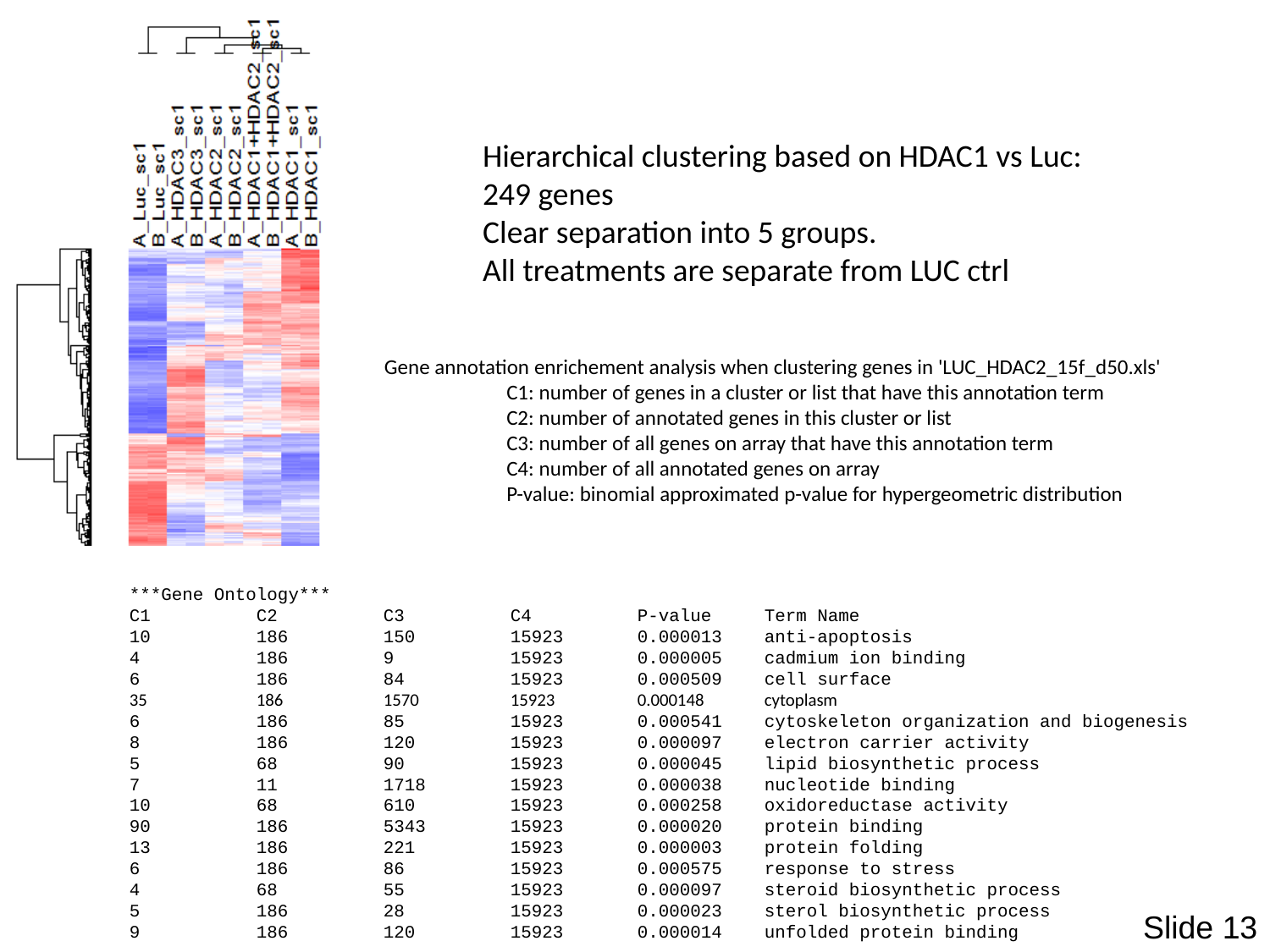

Hierarchical clustering based on HDAC1 vs Luc:
249 genes
Clear separation into 5 groups.
All treatments are separate from LUC ctrl
 Gene annotation enrichement analysis when clustering genes in 'LUC_HDAC2_15f_d50.xls'
	C1: number of genes in a cluster or list that have this annotation term
	C2: number of annotated genes in this cluster or list
	C3: number of all genes on array that have this annotation term
	C4: number of all annotated genes on array
	P-value: binomial approximated p-value for hypergeometric distribution
	***Gene Ontology***
	C1	C2	C3	C4	P-value	Term Name
	10	186	150	15923	0.000013	anti-apoptosis
	4	186	9	15923	0.000005	cadmium ion binding
	6	186	84	15923	0.000509	cell surface
	35	186	1570	15923	0.000148	cytoplasm
	6	186	85	15923	0.000541	cytoskeleton organization and biogenesis
	8	186	120	15923	0.000097	electron carrier activity
	5	68	90	15923	0.000045	lipid biosynthetic process
	7	11	1718	15923	0.000038	nucleotide binding
	10	68	610	15923	0.000258	oxidoreductase activity
	90	186	5343	15923	0.000020	protein binding
	13	186	221	15923	0.000003	protein folding
	6	186	86	15923	0.000575	response to stress
	4	68	55	15923	0.000097	steroid biosynthetic process
	5	186	28	15923	0.000023	sterol biosynthetic process
	9	186	120	15923	0.000014	unfolded protein binding
Slide <number>

## Slide 14
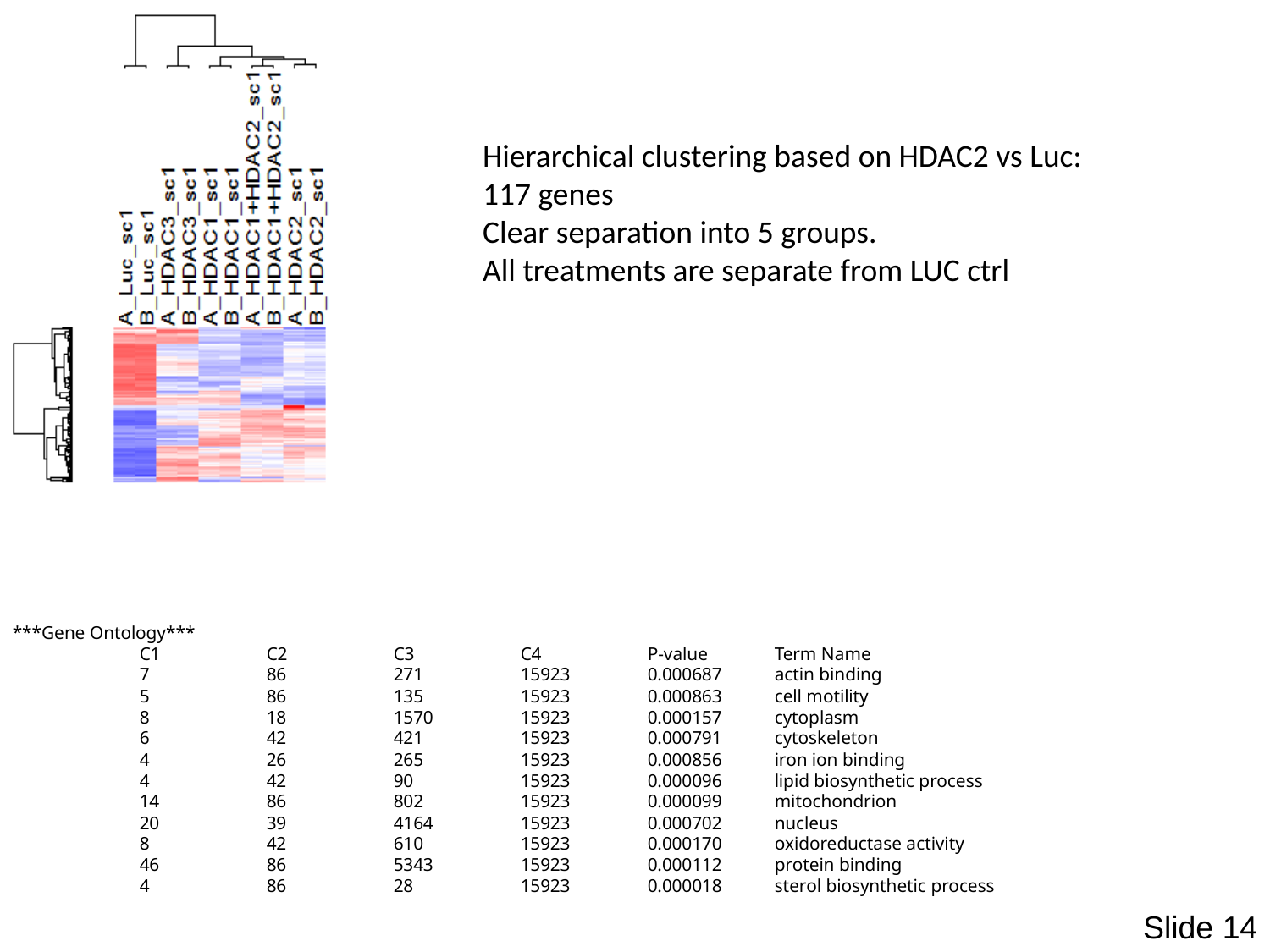

Hierarchical clustering based on HDAC2 vs Luc:
117 genes
Clear separation into 5 groups.
All treatments are separate from LUC ctrl
***Gene Ontology***
	C1	C2	C3	C4	P-value	Term Name
	7	86	271	15923	0.000687	actin binding
	5	86	135	15923	0.000863	cell motility
	8	18	1570	15923	0.000157	cytoplasm
	6	42	421	15923	0.000791	cytoskeleton
	4	26	265	15923	0.000856	iron ion binding
	4	42	90	15923	0.000096	lipid biosynthetic process
	14	86	802	15923	0.000099	mitochondrion
	20	39	4164	15923	0.000702	nucleus
	8	42	610	15923	0.000170	oxidoreductase activity
	46	86	5343	15923	0.000112	protein binding
	4	86	28	15923	0.000018	sterol biosynthetic process
Slide <number>

## Slide 15
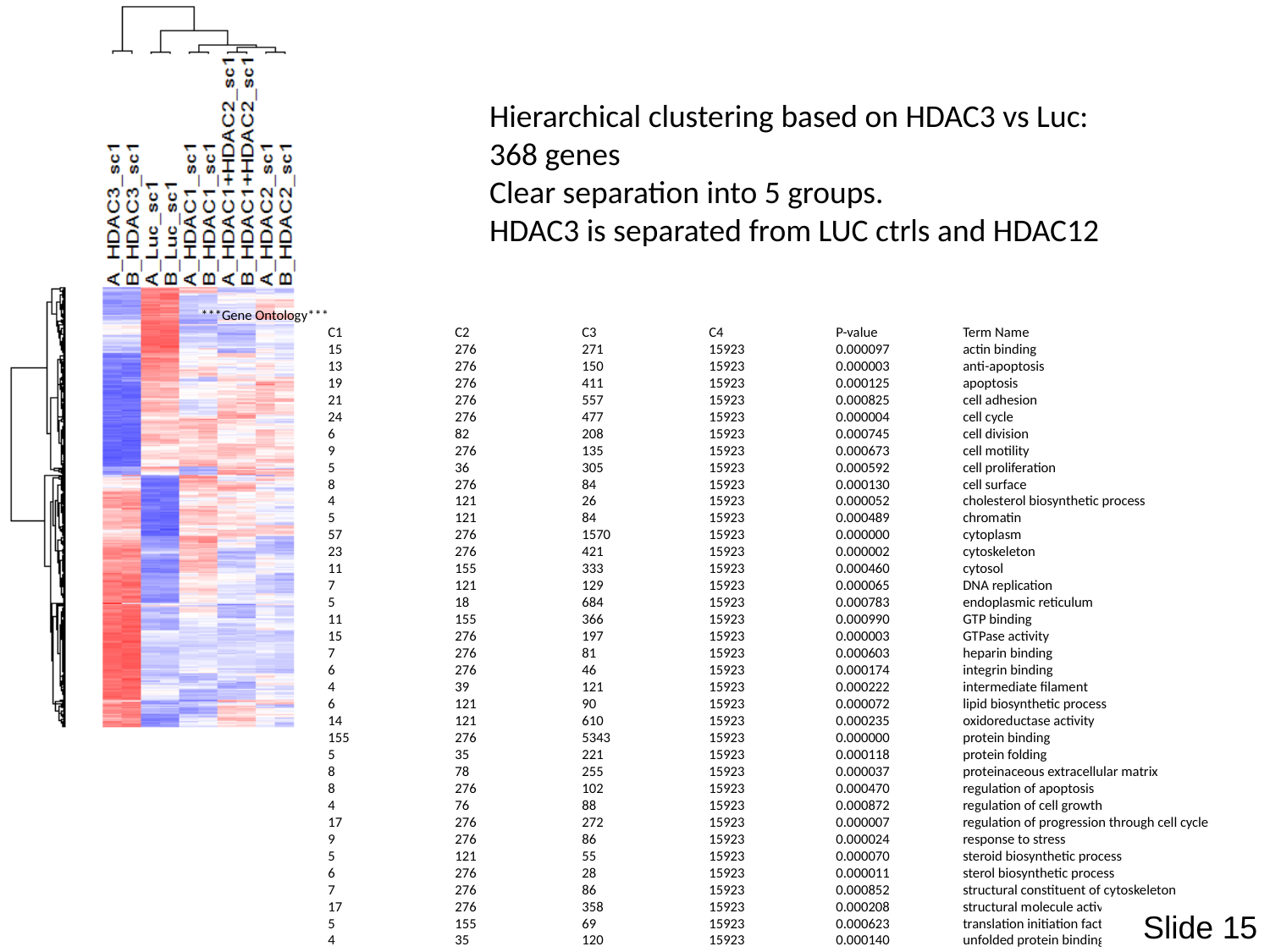

Hierarchical clustering based on HDAC3 vs Luc:
368 genes
Clear separation into 5 groups.
HDAC3 is separated from LUC ctrls and HDAC12
***Gene Ontology***
	C1	C2	C3	C4	P-value	Term Name
	15	276	271	15923	0.000097	actin binding
	13	276	150	15923	0.000003	anti-apoptosis
	19	276	411	15923	0.000125	apoptosis
	21	276	557	15923	0.000825	cell adhesion
	24	276	477	15923	0.000004	cell cycle
	6	82	208	15923	0.000745	cell division
	9	276	135	15923	0.000673	cell motility
	5	36	305	15923	0.000592	cell proliferation
	8	276	84	15923	0.000130	cell surface
	4	121	26	15923	0.000052	cholesterol biosynthetic process
	5	121	84	15923	0.000489	chromatin
	57	276	1570	15923	0.000000	cytoplasm
	23	276	421	15923	0.000002	cytoskeleton
	11	155	333	15923	0.000460	cytosol
	7	121	129	15923	0.000065	DNA replication
	5	18	684	15923	0.000783	endoplasmic reticulum
	11	155	366	15923	0.000990	GTP binding
	15	276	197	15923	0.000003	GTPase activity
	7	276	81	15923	0.000603	heparin binding
	6	276	46	15923	0.000174	integrin binding
	4	39	121	15923	0.000222	intermediate filament
	6	121	90	15923	0.000072	lipid biosynthetic process
	14	121	610	15923	0.000235	oxidoreductase activity
	155	276	5343	15923	0.000000	protein binding
	5	35	221	15923	0.000118	protein folding
	8	78	255	15923	0.000037	proteinaceous extracellular matrix
	8	276	102	15923	0.000470	regulation of apoptosis
	4	76	88	15923	0.000872	regulation of cell growth
	17	276	272	15923	0.000007	regulation of progression through cell cycle
	9	276	86	15923	0.000024	response to stress
	5	121	55	15923	0.000070	steroid biosynthetic process
	6	276	28	15923	0.000011	sterol biosynthetic process
	7	276	86	15923	0.000852	structural constituent of cytoskeleton
	17	276	358	15923	0.000208	structural molecule activity
	5	155	69	15923	0.000623	translation initiation factor activity
	4	35	120	15923	0.000140	unfolded protein binding
Slide <number>

## Slide 16
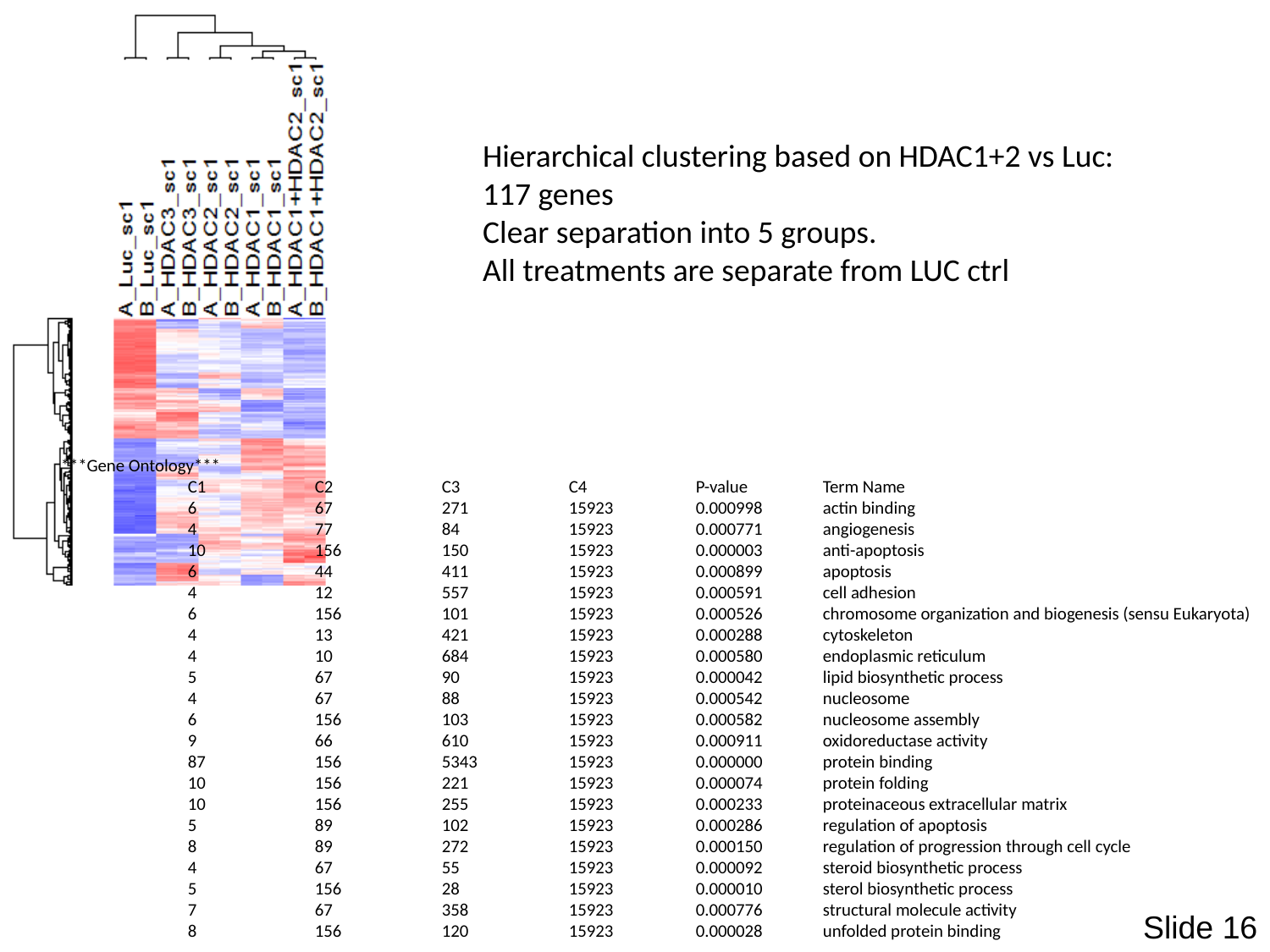

Hierarchical clustering based on HDAC1+2 vs Luc:
117 genes
Clear separation into 5 groups.
All treatments are separate from LUC ctrl
***Gene Ontology***
	C1	C2	C3	C4	P-value	Term Name
	6	67	271	15923	0.000998	actin binding
	4	77	84	15923	0.000771	angiogenesis
	10	156	150	15923	0.000003	anti-apoptosis
	6	44	411	15923	0.000899	apoptosis
	4	12	557	15923	0.000591	cell adhesion
	6	156	101	15923	0.000526	chromosome organization and biogenesis (sensu Eukaryota)
	4	13	421	15923	0.000288	cytoskeleton
	4	10	684	15923	0.000580	endoplasmic reticulum
	5	67	90	15923	0.000042	lipid biosynthetic process
	4	67	88	15923	0.000542	nucleosome
	6	156	103	15923	0.000582	nucleosome assembly
	9	66	610	15923	0.000911	oxidoreductase activity
	87	156	5343	15923	0.000000	protein binding
	10	156	221	15923	0.000074	protein folding
	10	156	255	15923	0.000233	proteinaceous extracellular matrix
	5	89	102	15923	0.000286	regulation of apoptosis
	8	89	272	15923	0.000150	regulation of progression through cell cycle
	4	67	55	15923	0.000092	steroid biosynthetic process
	5	156	28	15923	0.000010	sterol biosynthetic process
	7	67	358	15923	0.000776	structural molecule activity
	8	156	120	15923	0.000028	unfolded protein binding
Slide <number>

## Slide 17
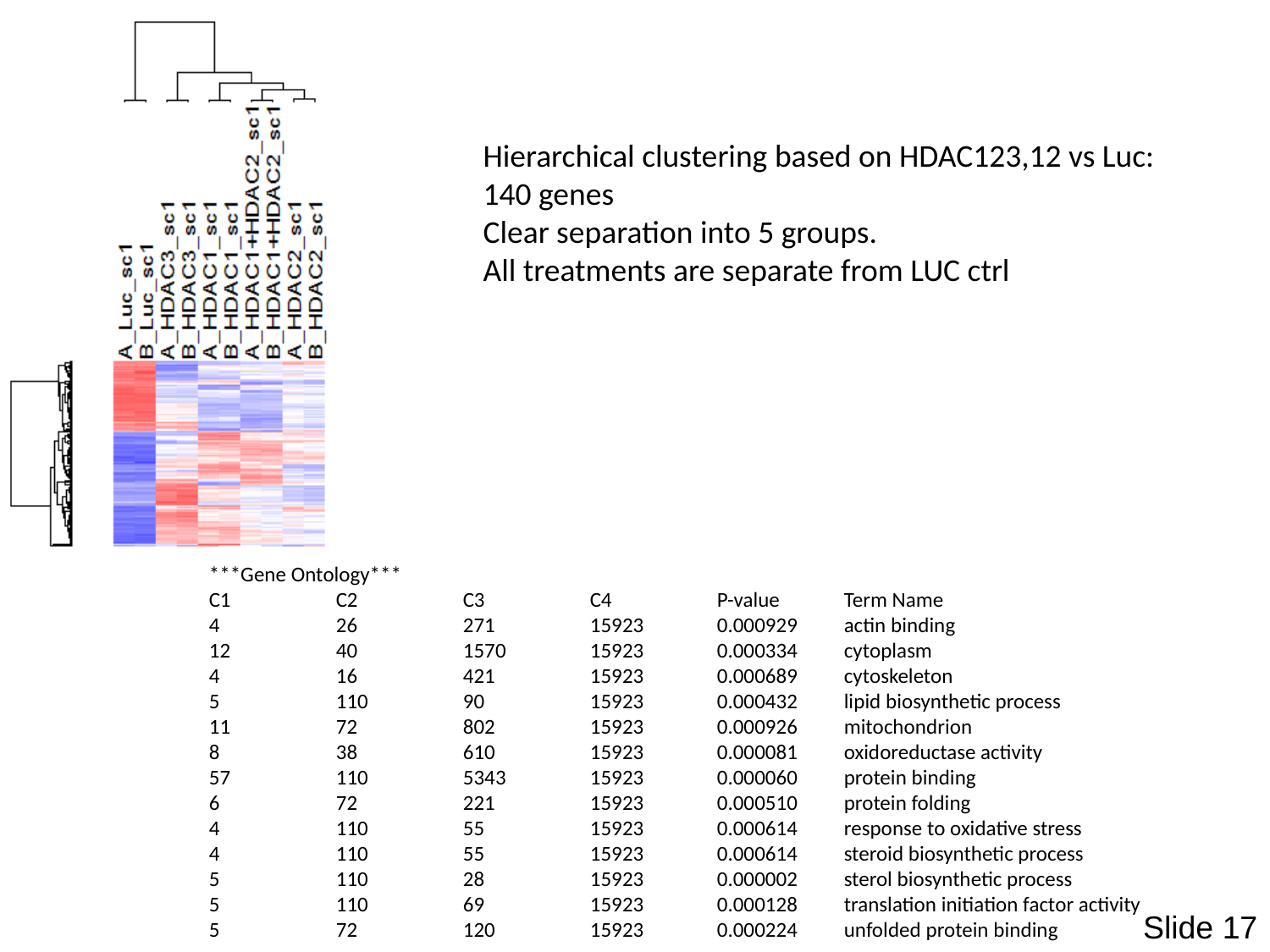

Hierarchical clustering based on HDAC123,12 vs Luc:
140 genes
Clear separation into 5 groups.
All treatments are separate from LUC ctrl
	***Gene Ontology***
	C1	C2	C3	C4	P-value	Term Name
	4	26	271	15923	0.000929	actin binding
	12	40	1570	15923	0.000334	cytoplasm
	4	16	421	15923	0.000689	cytoskeleton
	5	110	90	15923	0.000432	lipid biosynthetic process
	11	72	802	15923	0.000926	mitochondrion
	8	38	610	15923	0.000081	oxidoreductase activity
	57	110	5343	15923	0.000060	protein binding
	6	72	221	15923	0.000510	protein folding
	4	110	55	15923	0.000614	response to oxidative stress
	4	110	55	15923	0.000614	steroid biosynthetic process
	5	110	28	15923	0.000002	sterol biosynthetic process
	5	110	69	15923	0.000128	translation initiation factor activity
	5	72	120	15923	0.000224	unfolded protein binding
Slide <number>

## Slide 18
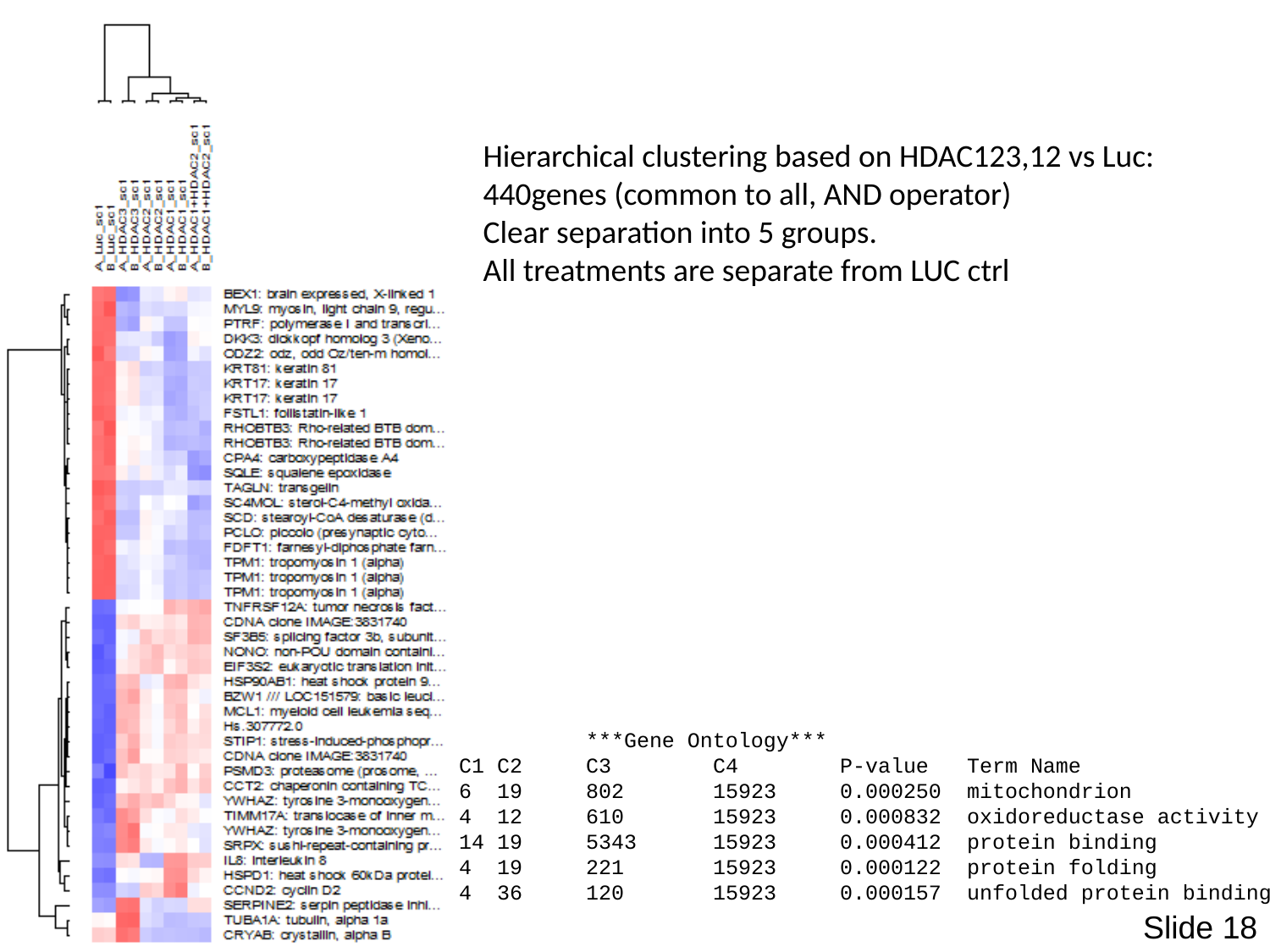

Hierarchical clustering based on HDAC123,12 vs Luc:
440genes (common to all, AND operator)
Clear separation into 5 groups.
All treatments are separate from LUC ctrl
	***Gene Ontology***
C1 C2	C3	C4	P-value	Term Name
6 19	802	15923	0.000250	mitochondrion
4 12	610	15923	0.000832	oxidoreductase activity
14 19	5343	15923	0.000412	protein binding
4 19	221	15923	0.000122	protein folding
4 36	120	15923	0.000157	unfolded protein binding
Slide <number>

## Slide 19
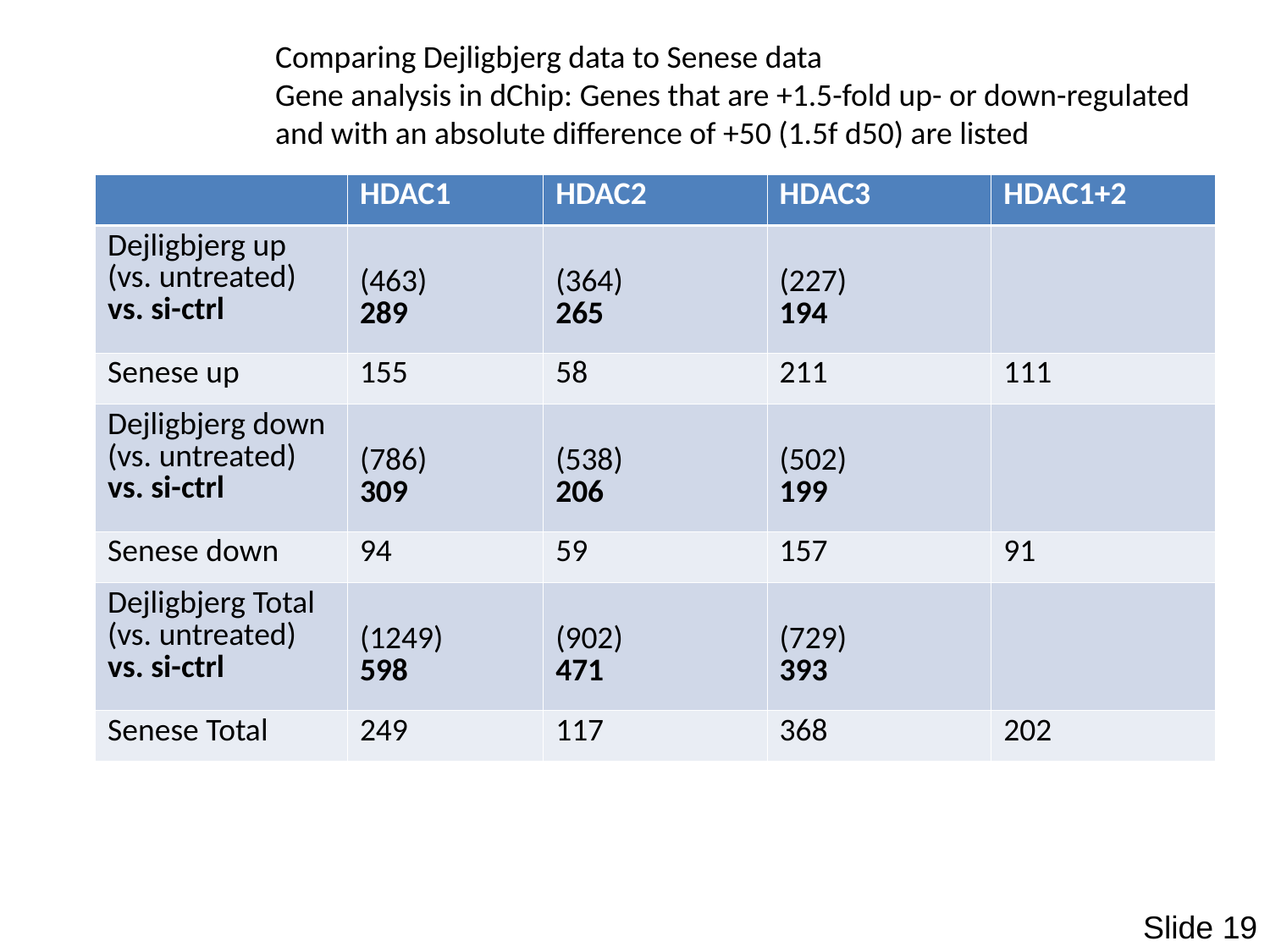

Comparing Dejligbjerg data to Senese data
Gene analysis in dChip: Genes that are +1.5-fold up- or down-regulated
and with an absolute difference of +50 (1.5f d50) are listed
| | HDAC1 | HDAC2 | HDAC3 | HDAC1+2 |
| --- | --- | --- | --- | --- |
| Dejligbjerg up (vs. untreated) vs. si-ctrl | (463) 289 | (364) 265 | (227) 194 | |
| Senese up | 155 | 58 | 211 | 111 |
| Dejligbjerg down (vs. untreated) vs. si-ctrl | (786) 309 | (538) 206 | (502) 199 | |
| Senese down | 94 | 59 | 157 | 91 |
| Dejligbjerg Total (vs. untreated) vs. si-ctrl | (1249) 598 | (902) 471 | (729) 393 | |
| Senese Total | 249 | 117 | 368 | 202 |
Slide <number>

## Slide 20
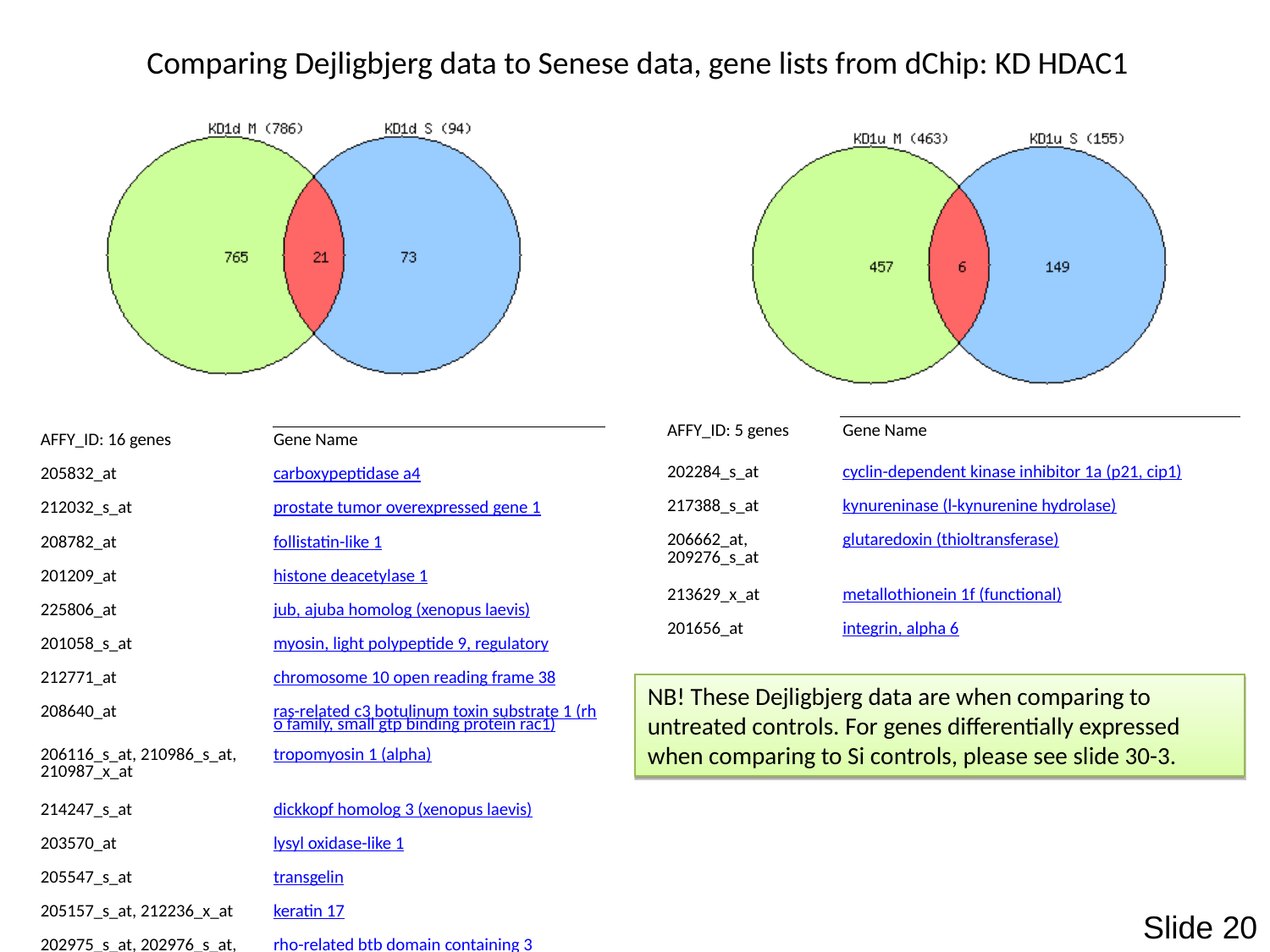

Comparing Dejligbjerg data to Senese data, gene lists from dChip: KD HDAC1
| AFFY\_ID: 5 genes | Gene Name |
| --- | --- |
| 202284\_s\_at | cyclin-dependent kinase inhibitor 1a (p21, cip1) |
| 217388\_s\_at | kynureninase (l-kynurenine hydrolase) |
| 206662\_at, 209276\_s\_at | glutaredoxin (thioltransferase) |
| 213629\_x\_at | metallothionein 1f (functional) |
| 201656\_at | integrin, alpha 6 |
| AFFY\_ID: 16 genes | Gene Name |
| --- | --- |
| 205832\_at | carboxypeptidase a4 |
| 212032\_s\_at | prostate tumor overexpressed gene 1 |
| 208782\_at | follistatin-like 1 |
| 201209\_at | histone deacetylase 1 |
| 225806\_at | jub, ajuba homolog (xenopus laevis) |
| 201058\_s\_at | myosin, light polypeptide 9, regulatory |
| 212771\_at | chromosome 10 open reading frame 38 |
| 208640\_at | ras-related c3 botulinum toxin substrate 1 (rho family, small gtp binding protein rac1) |
| 206116\_s\_at, 210986\_s\_at, 210987\_x\_at | tropomyosin 1 (alpha) |
| 214247\_s\_at | dickkopf homolog 3 (xenopus laevis) |
| 203570\_at | lysyl oxidase-like 1 |
| 205547\_s\_at | transgelin |
| 205157\_s\_at, 212236\_x\_at | keratin 17 |
| 202975\_s\_at, 202976\_s\_at, 225202\_at | rho-related btb domain containing 3 |
| 225275\_at | egf-like repeats and discoidin i-like domains 3 |
| 228158\_at | dopamine receptor d5 pseudogene 2 |
NB! These Dejligbjerg data are when comparing to
untreated controls. For genes differentially expressed
when comparing to Si controls, please see slide 30-3.
Slide <number>

## Slide 21
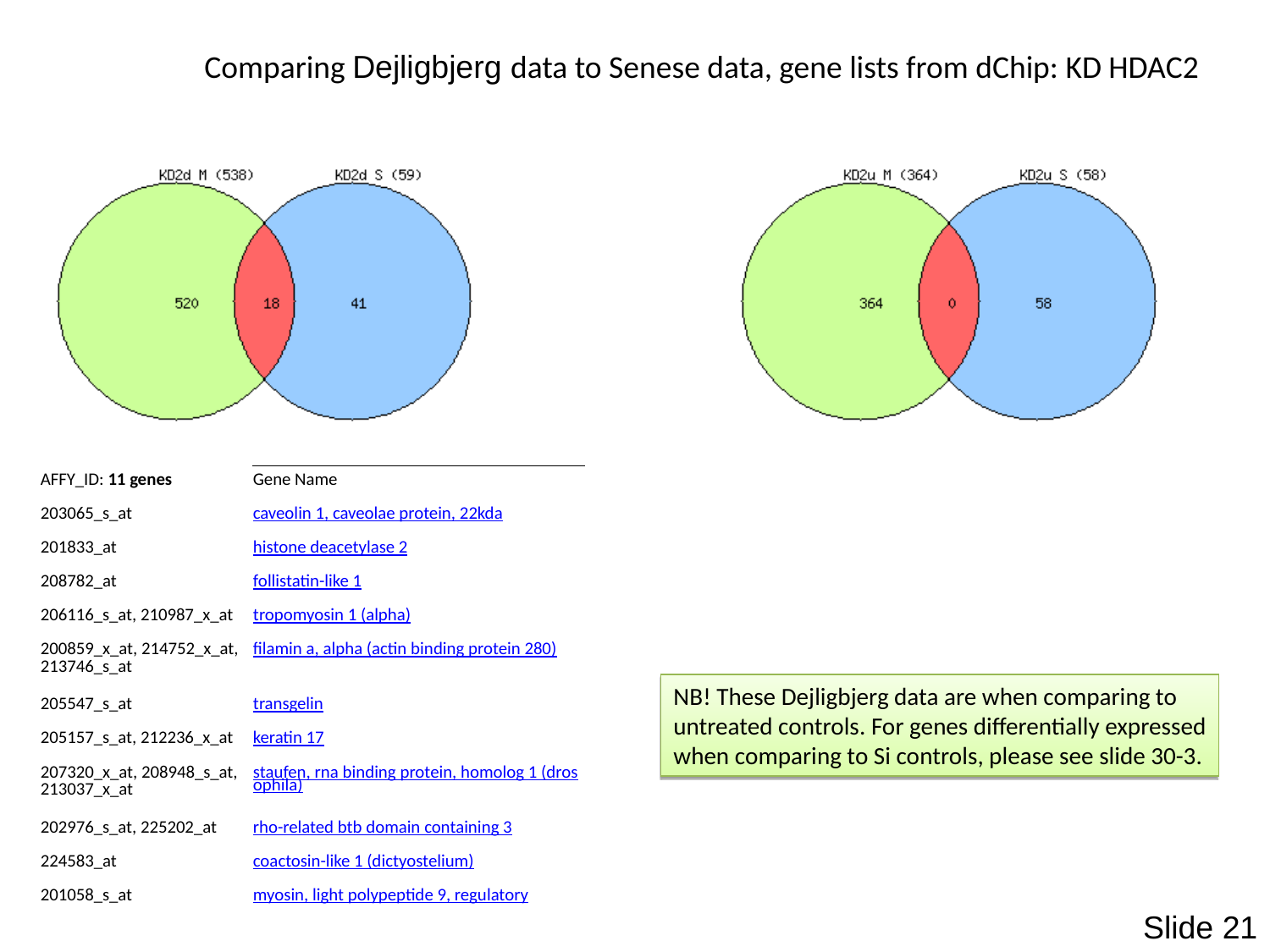

Comparing Dejligbjerg data to Senese data, gene lists from dChip: KD HDAC2
| AFFY\_ID: 11 genes | Gene Name |
| --- | --- |
| 203065\_s\_at | caveolin 1, caveolae protein, 22kda |
| 201833\_at | histone deacetylase 2 |
| 208782\_at | follistatin-like 1 |
| 206116\_s\_at, 210987\_x\_at | tropomyosin 1 (alpha) |
| 200859\_x\_at, 214752\_x\_at, 213746\_s\_at | filamin a, alpha (actin binding protein 280) |
| 205547\_s\_at | transgelin |
| 205157\_s\_at, 212236\_x\_at | keratin 17 |
| 207320\_x\_at, 208948\_s\_at, 213037\_x\_at | staufen, rna binding protein, homolog 1 (drosophila) |
| 202976\_s\_at, 225202\_at | rho-related btb domain containing 3 |
| 224583\_at | coactosin-like 1 (dictyostelium) |
| 201058\_s\_at | myosin, light polypeptide 9, regulatory |
NB! These Dejligbjerg data are when comparing to
untreated controls. For genes differentially expressed
when comparing to Si controls, please see slide 30-3.
Slide <number>

## Slide 22
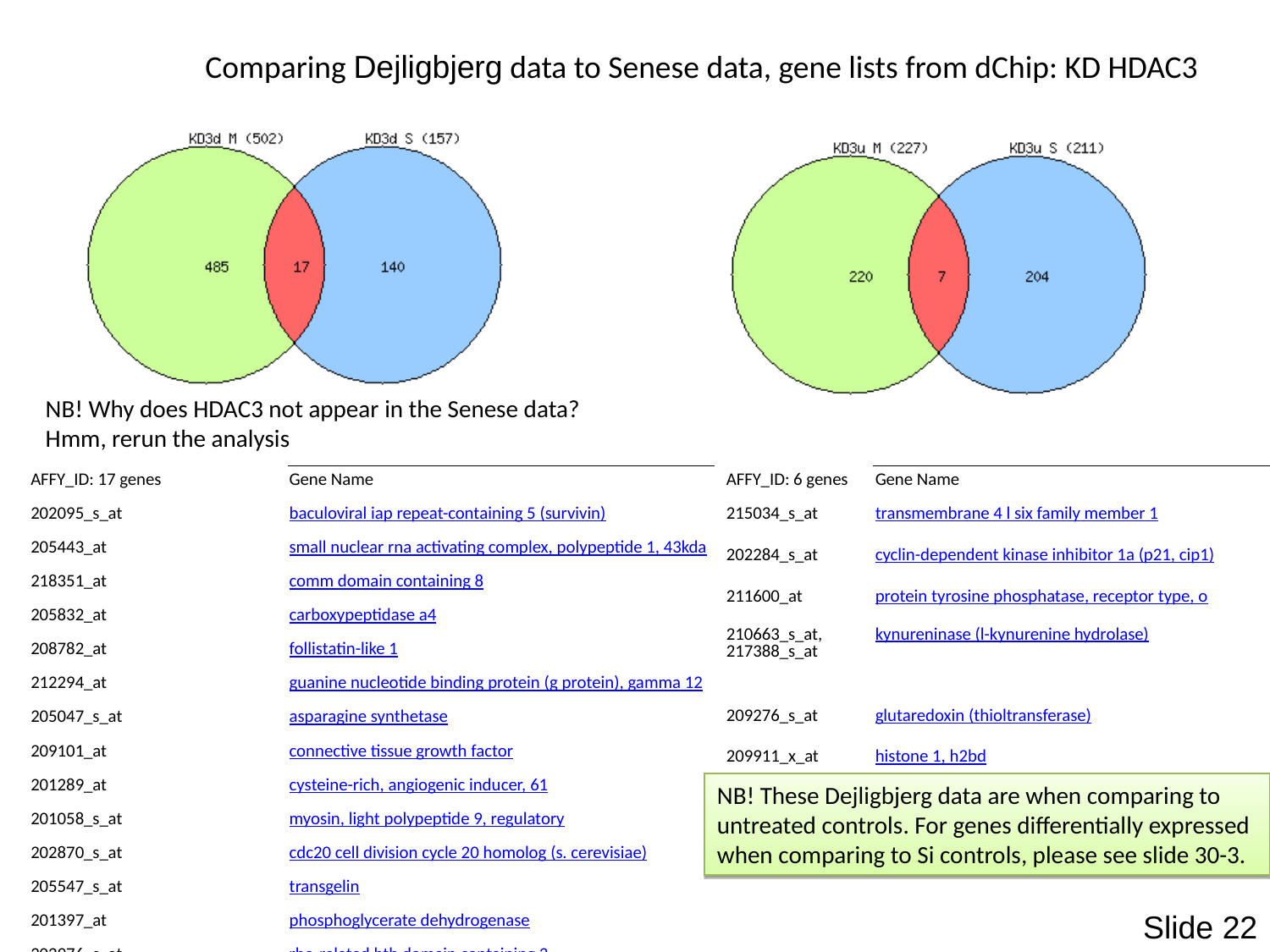

Comparing Dejligbjerg data to Senese data, gene lists from dChip: KD HDAC3
NB! Why does HDAC3 not appear in the Senese data?
Hmm, rerun the analysis
| AFFY\_ID: 17 genes | Gene Name |
| --- | --- |
| 202095\_s\_at | baculoviral iap repeat-containing 5 (survivin) |
| 205443\_at | small nuclear rna activating complex, polypeptide 1, 43kda |
| 218351\_at | comm domain containing 8 |
| 205832\_at | carboxypeptidase a4 |
| 208782\_at | follistatin-like 1 |
| 212294\_at | guanine nucleotide binding protein (g protein), gamma 12 |
| 205047\_s\_at | asparagine synthetase |
| 209101\_at | connective tissue growth factor |
| 201289\_at | cysteine-rich, angiogenic inducer, 61 |
| 201058\_s\_at | myosin, light polypeptide 9, regulatory |
| 202870\_s\_at | cdc20 cell division cycle 20 homolog (s. cerevisiae) |
| 205547\_s\_at | transgelin |
| 201397\_at | phosphoglycerate dehydrogenase |
| 202976\_s\_at | rho-related btb domain containing 3 |
| 212352\_s\_at | transmembrane emp24-like trafficking protein 10 (yeast) |
| 228121\_at | transforming growth factor, beta 2 |
| 212457\_at | transcription factor binding to ighm enhancer 3 |
| AFFY\_ID: 6 genes | Gene Name |
| --- | --- |
| 215034\_s\_at | transmembrane 4 l six family member 1 |
| 202284\_s\_at | cyclin-dependent kinase inhibitor 1a (p21, cip1) |
| 211600\_at | protein tyrosine phosphatase, receptor type, o |
| 210663\_s\_at, 217388\_s\_at | kynureninase (l-kynurenine hydrolase) |
| 209276\_s\_at | glutaredoxin (thioltransferase) |
| 209911\_x\_at | histone 1, h2bd |
NB! These Dejligbjerg data are when comparing to
untreated controls. For genes differentially expressed
when comparing to Si controls, please see slide 30-3.
Slide <number>

## Slide 23
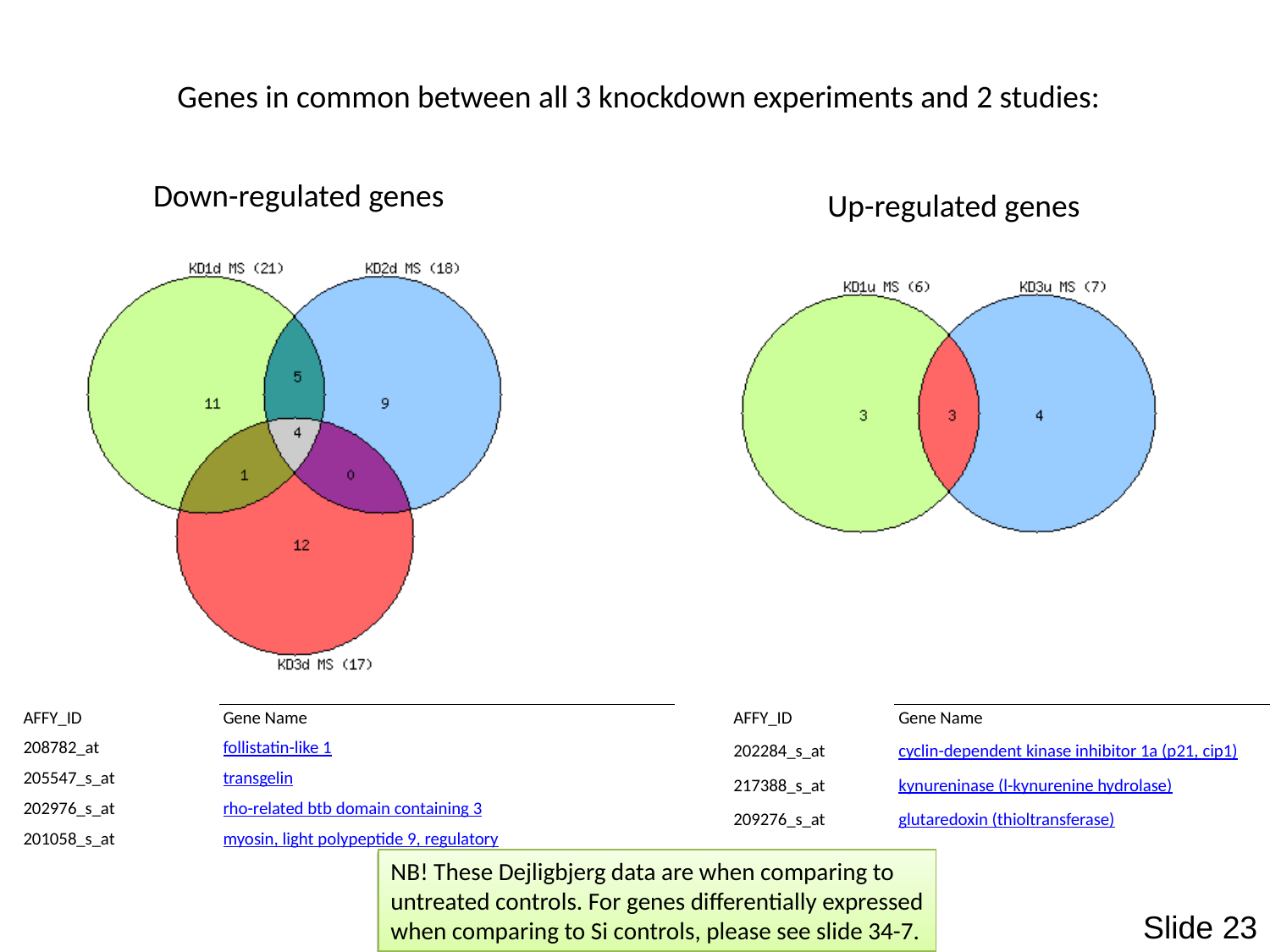

Genes in common between all 3 knockdown experiments and 2 studies:
Down-regulated genes
Up-regulated genes
| AFFY\_ID | Gene Name |
| --- | --- |
| 208782\_at | follistatin-like 1 |
| 205547\_s\_at | transgelin |
| 202976\_s\_at | rho-related btb domain containing 3 |
| 201058\_s\_at | myosin, light polypeptide 9, regulatory |
| AFFY\_ID | Gene Name |
| --- | --- |
| 202284\_s\_at | cyclin-dependent kinase inhibitor 1a (p21, cip1) |
| 217388\_s\_at | kynureninase (l-kynurenine hydrolase) |
| 209276\_s\_at | glutaredoxin (thioltransferase) |
NB! These Dejligbjerg data are when comparing to
untreated controls. For genes differentially expressed
when comparing to Si controls, please see slide 34-7.
Slide <number>

## Slide 24
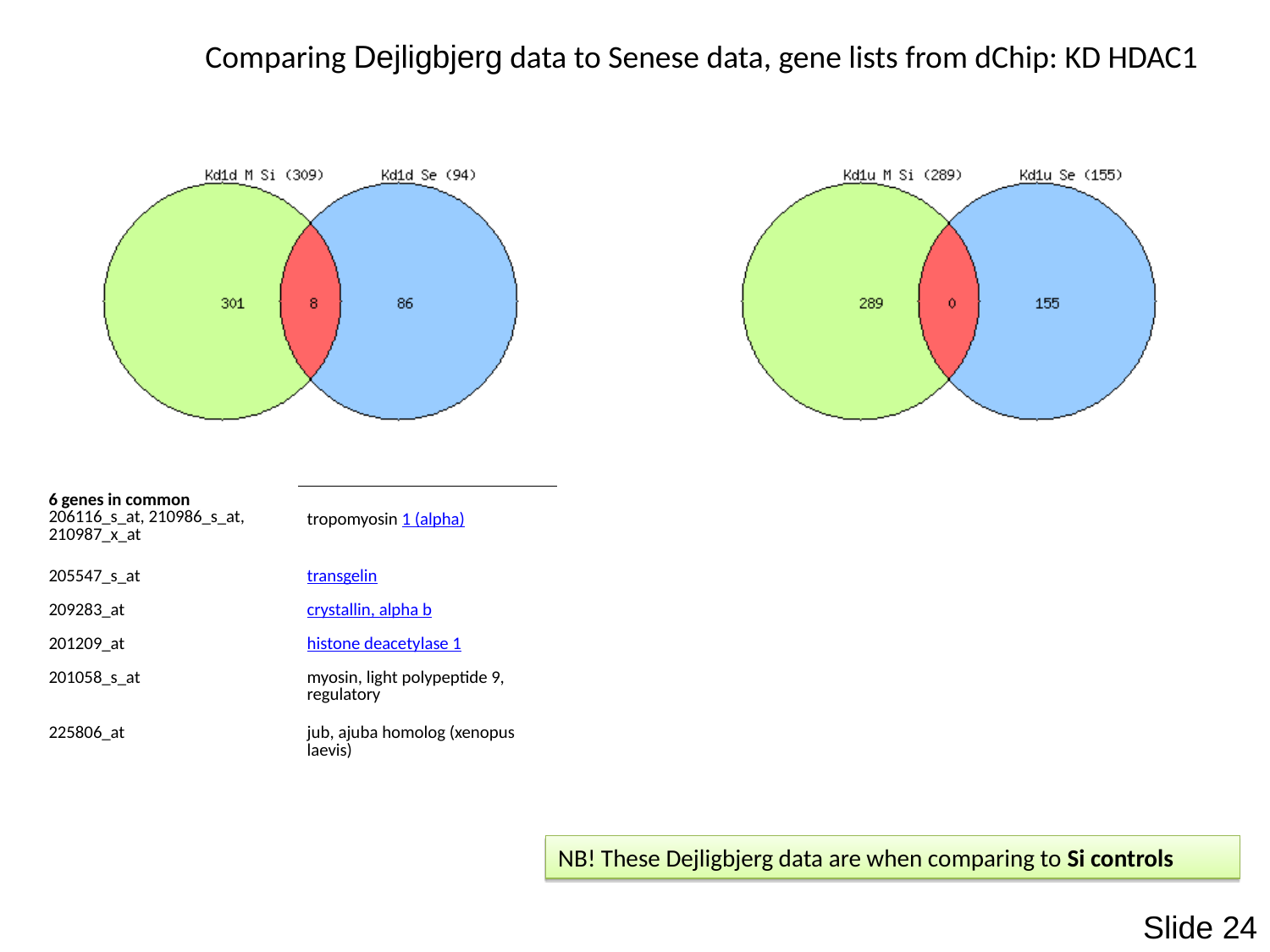

Comparing Dejligbjerg data to Senese data, gene lists from dChip: KD HDAC1
| 6 genes in common 206116\_s\_at, 210986\_s\_at, 210987\_x\_at | tropomyosin 1 (alpha) |
| --- | --- |
| 205547\_s\_at | transgelin |
| 209283\_at | crystallin, alpha b |
| 201209\_at | histone deacetylase 1 |
| 201058\_s\_at | myosin, light polypeptide 9, regulatory |
| 225806\_at | jub, ajuba homolog (xenopus laevis) |
NB! These Dejligbjerg data are when comparing to Si controls
Slide <number>

## Slide 25
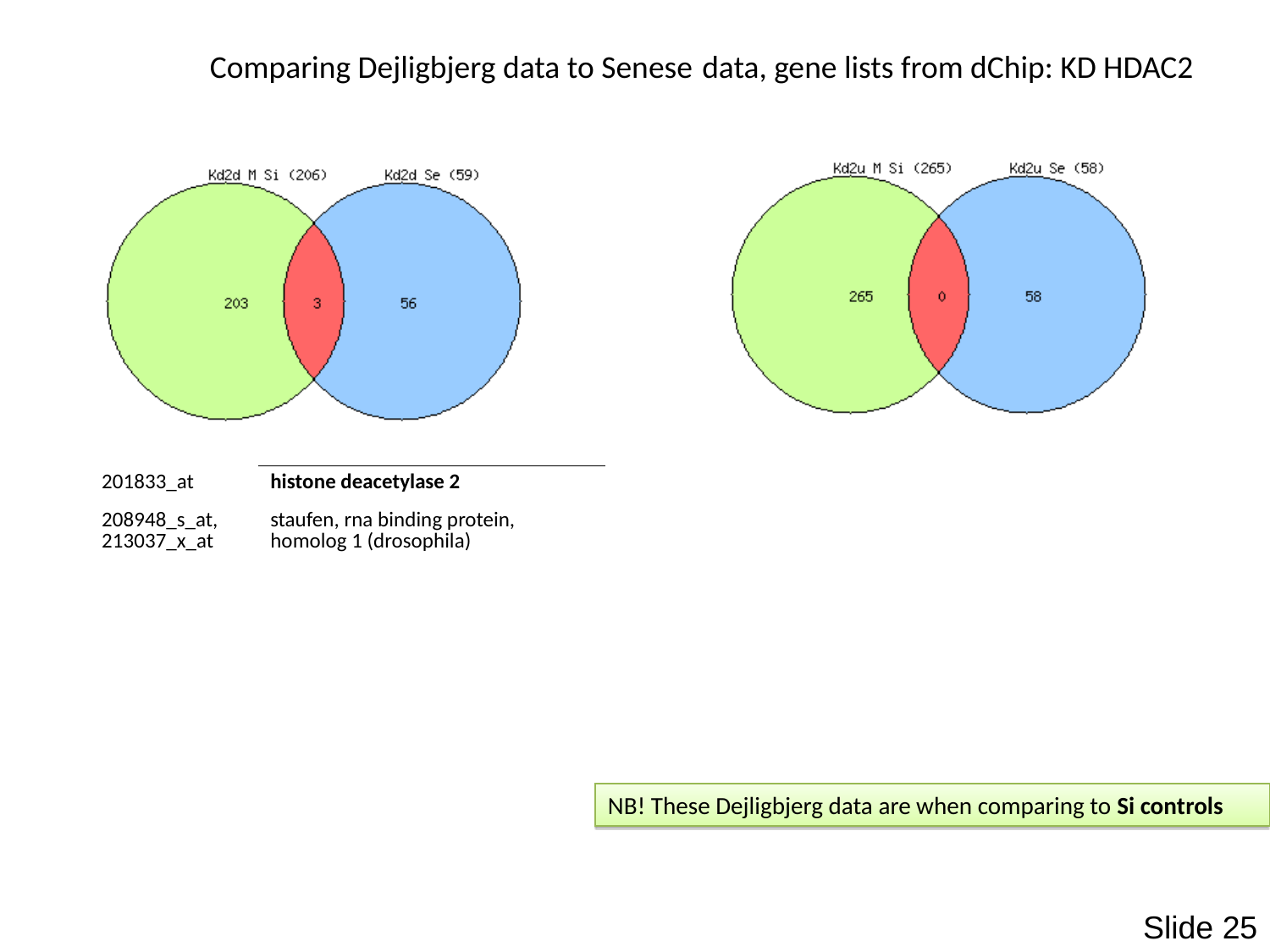

Comparing Dejligbjerg data to Senese data, gene lists from dChip: KD HDAC2
| 201833\_at | histone deacetylase 2 |
| --- | --- |
| 208948\_s\_at, 213037\_x\_at | staufen, rna binding protein, homolog 1 (drosophila) |
NB! These Dejligbjerg data are when comparing to Si controls
Slide <number>

## Slide 26
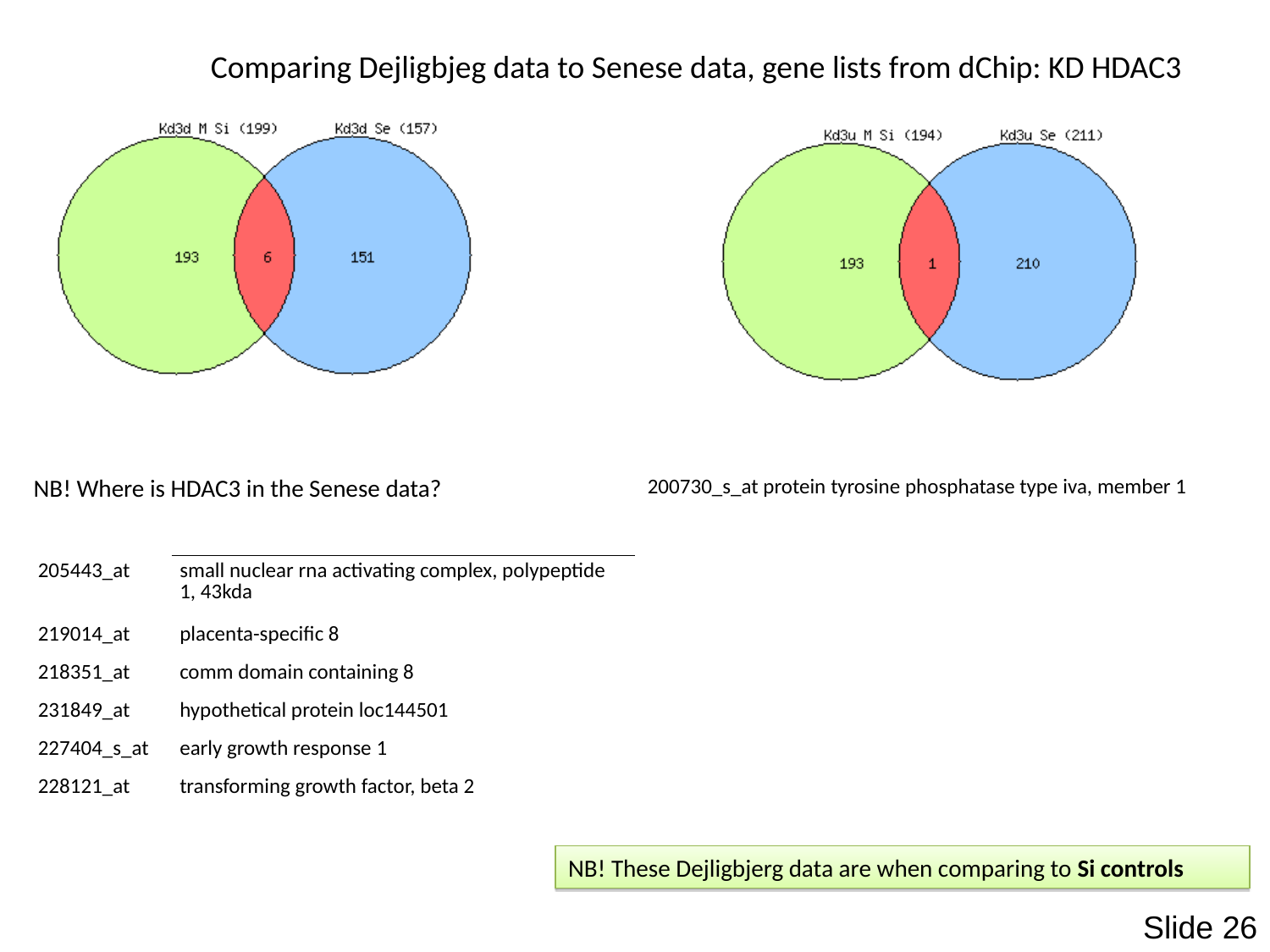

Comparing Dejligbjeg data to Senese data, gene lists from dChip: KD HDAC3
NB! Where is HDAC3 in the Senese data?
200730_s_at protein tyrosine phosphatase type iva, member 1
| 205443\_at | small nuclear rna activating complex, polypeptide 1, 43kda |
| --- | --- |
| 219014\_at | placenta-specific 8 |
| 218351\_at | comm domain containing 8 |
| 231849\_at | hypothetical protein loc144501 |
| 227404\_s\_at | early growth response 1 |
| 228121\_at | transforming growth factor, beta 2 |
NB! These Dejligbjerg data are when comparing to Si controls
Slide <number>

## Slide 27
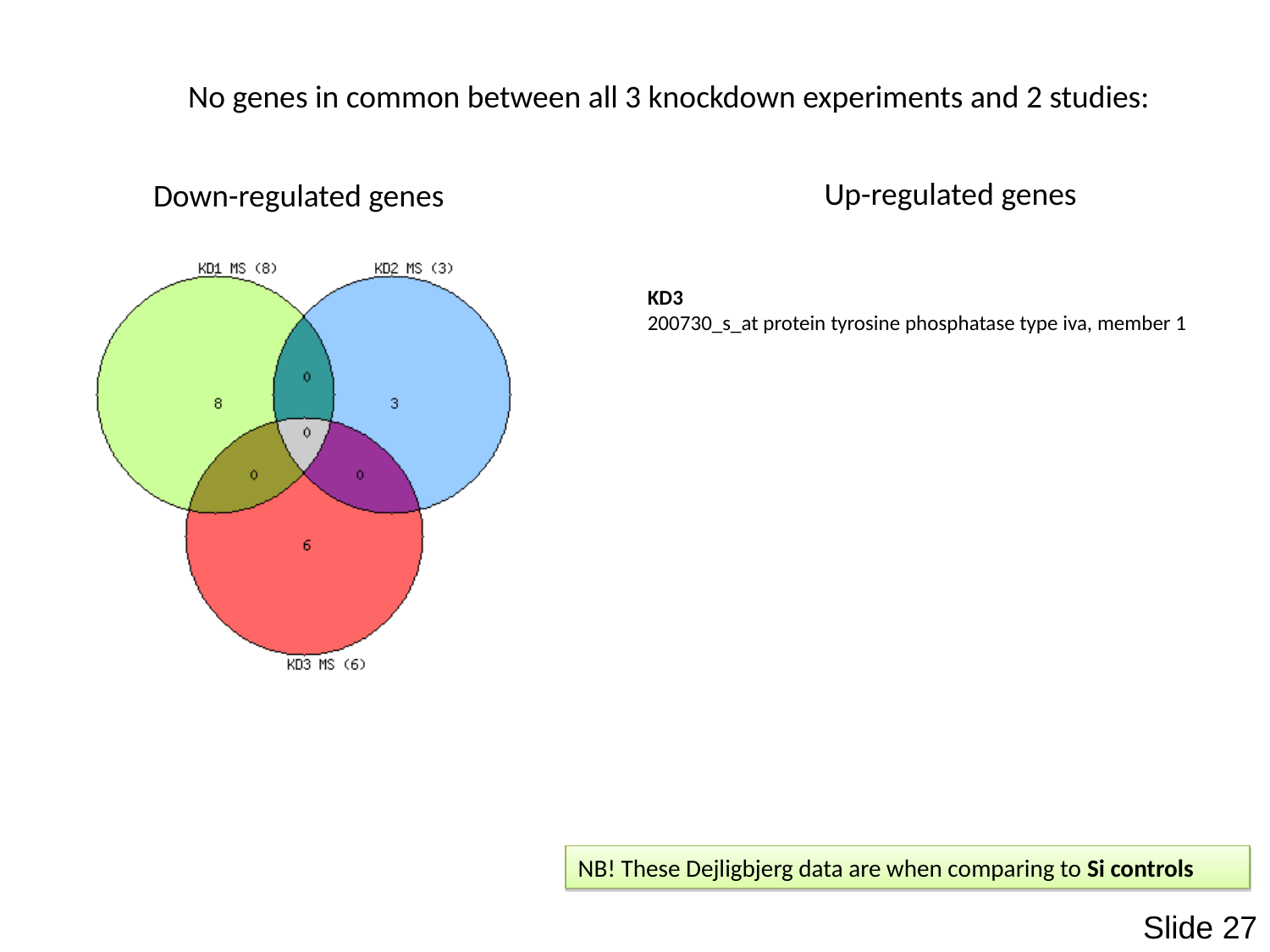

No genes in common between all 3 knockdown experiments and 2 studies:
Up-regulated genes
Down-regulated genes
KD3
200730_s_at protein tyrosine phosphatase type iva, member 1
NB! These Dejligbjerg data are when comparing to Si controls
Slide <number>

## Slide 28
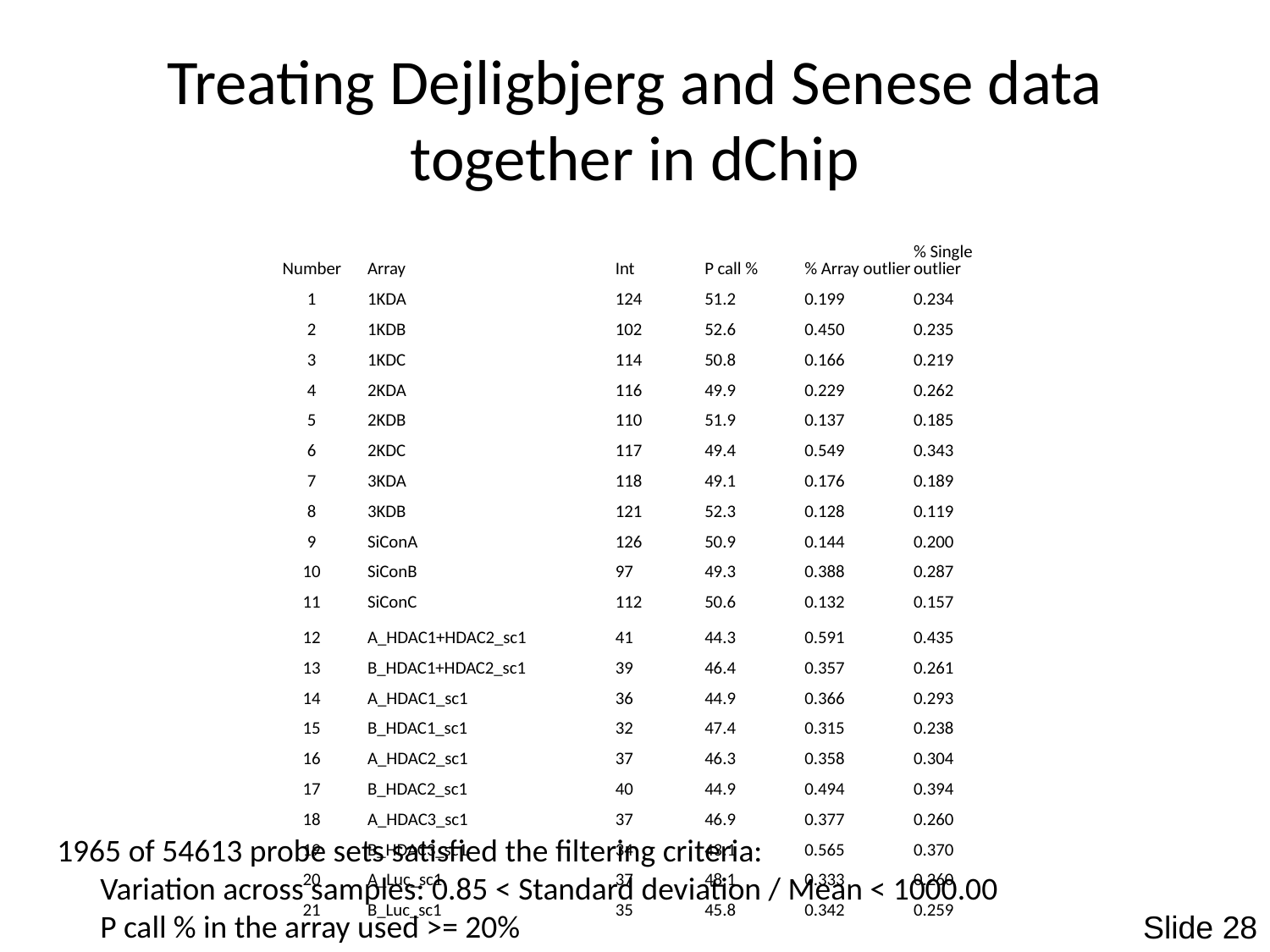

# Treating Dejligbjerg and Senese data together in dChip
| Number | Array | Int | P call % | % Array outlier | % Single outlier |
| --- | --- | --- | --- | --- | --- |
| 1 | 1KDA | 124 | 51.2 | 0.199 | 0.234 |
| 2 | 1KDB | 102 | 52.6 | 0.450 | 0.235 |
| 3 | 1KDC | 114 | 50.8 | 0.166 | 0.219 |
| 4 | 2KDA | 116 | 49.9 | 0.229 | 0.262 |
| 5 | 2KDB | 110 | 51.9 | 0.137 | 0.185 |
| 6 | 2KDC | 117 | 49.4 | 0.549 | 0.343 |
| 7 | 3KDA | 118 | 49.1 | 0.176 | 0.189 |
| 8 | 3KDB | 121 | 52.3 | 0.128 | 0.119 |
| 9 | SiConA | 126 | 50.9 | 0.144 | 0.200 |
| 10 | SiConB | 97 | 49.3 | 0.388 | 0.287 |
| 11 | SiConC | 112 | 50.6 | 0.132 | 0.157 |
| 12 | A\_HDAC1+HDAC2\_sc1 | 41 | 44.3 | 0.591 | 0.435 |
| 13 | B\_HDAC1+HDAC2\_sc1 | 39 | 46.4 | 0.357 | 0.261 |
| 14 | A\_HDAC1\_sc1 | 36 | 44.9 | 0.366 | 0.293 |
| 15 | B\_HDAC1\_sc1 | 32 | 47.4 | 0.315 | 0.238 |
| 16 | A\_HDAC2\_sc1 | 37 | 46.3 | 0.358 | 0.304 |
| 17 | B\_HDAC2\_sc1 | 40 | 44.9 | 0.494 | 0.394 |
| 18 | A\_HDAC3\_sc1 | 37 | 46.9 | 0.377 | 0.260 |
| 19 | B\_HDAC3\_sc1 | 34 | 43.1 | 0.565 | 0.370 |
| 20 | A\_Luc\_sc1 | 37 | 48.1 | 0.333 | 0.260 |
| 21 | B\_Luc\_sc1 | 35 | 45.8 | 0.342 | 0.259 |
1965 of 54613 probe sets satisfied the filtering criteria:
 Variation across samples: 0.85 < Standard deviation / Mean < 1000.00
 P call % in the array used >= 20%
Slide <number>

## Slide 29
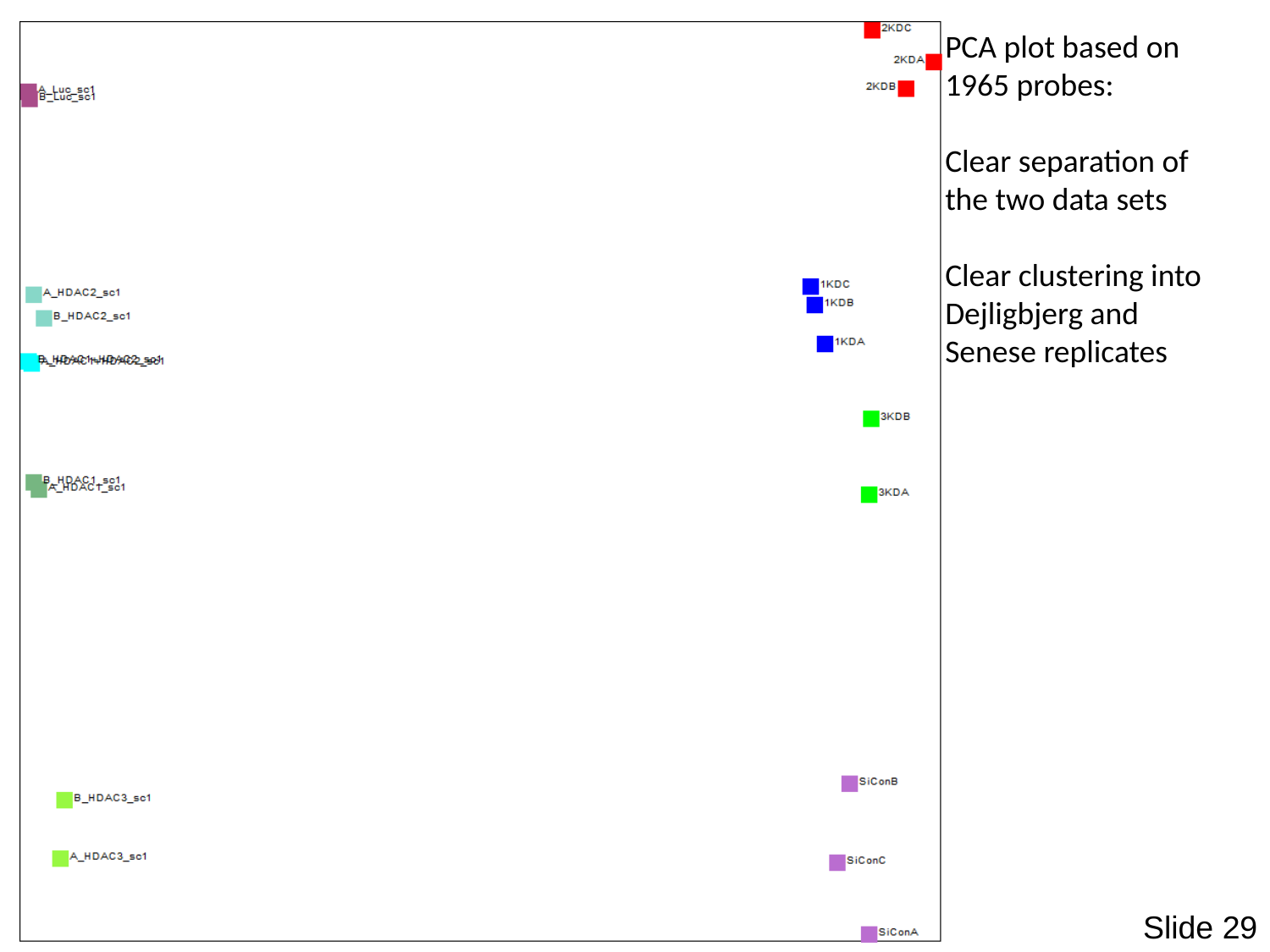

PCA plot based on 1965 probes:
Clear separation of the two data sets
Clear clustering into Dejligbjerg and Senese replicates
Slide <number>

## Slide 30
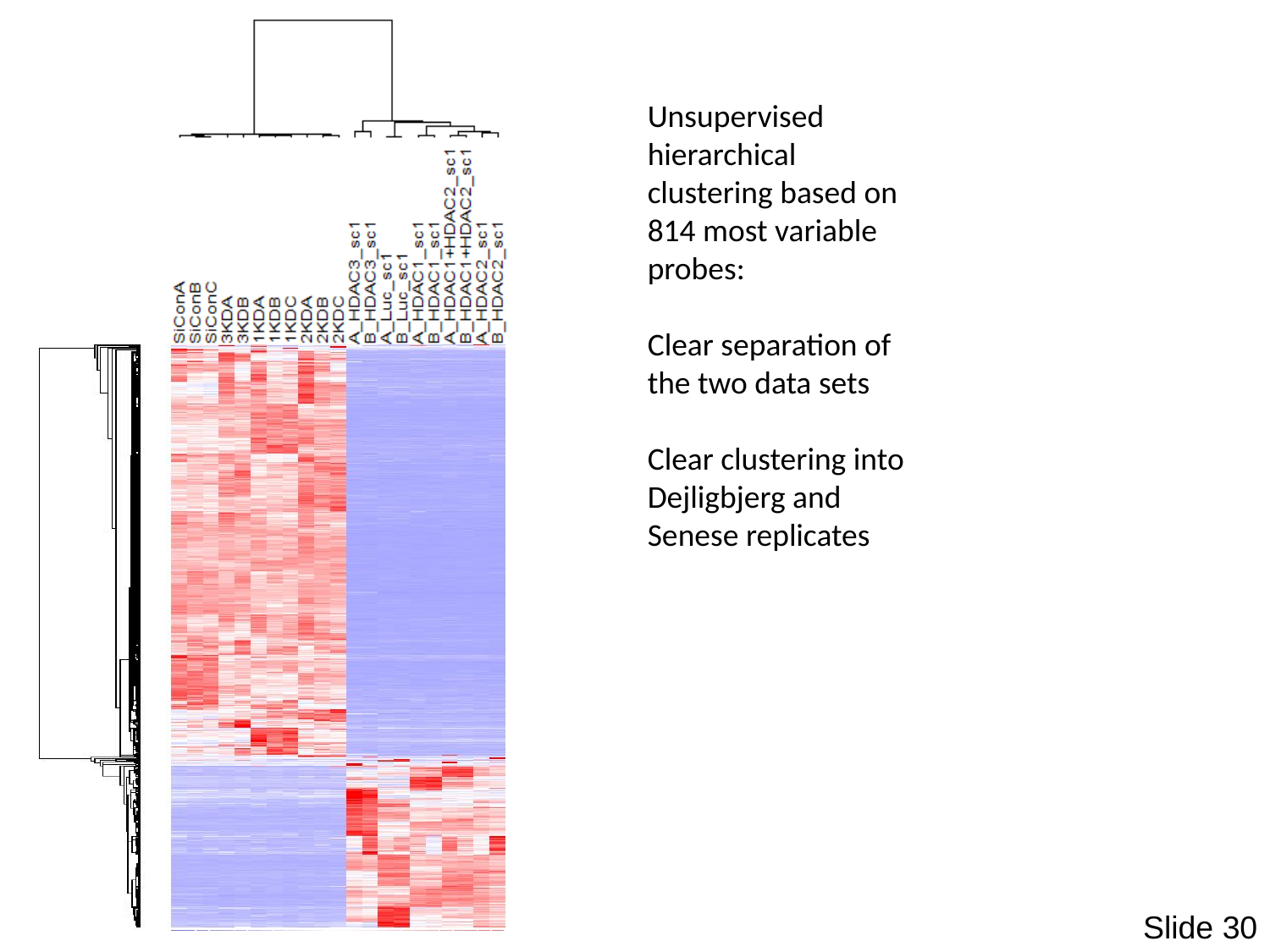

Unsupervised hierarchical clustering based on 814 most variable probes:
Clear separation of the two data sets
Clear clustering into Dejligbjerg and Senese replicates
Slide <number>

## Slide 31
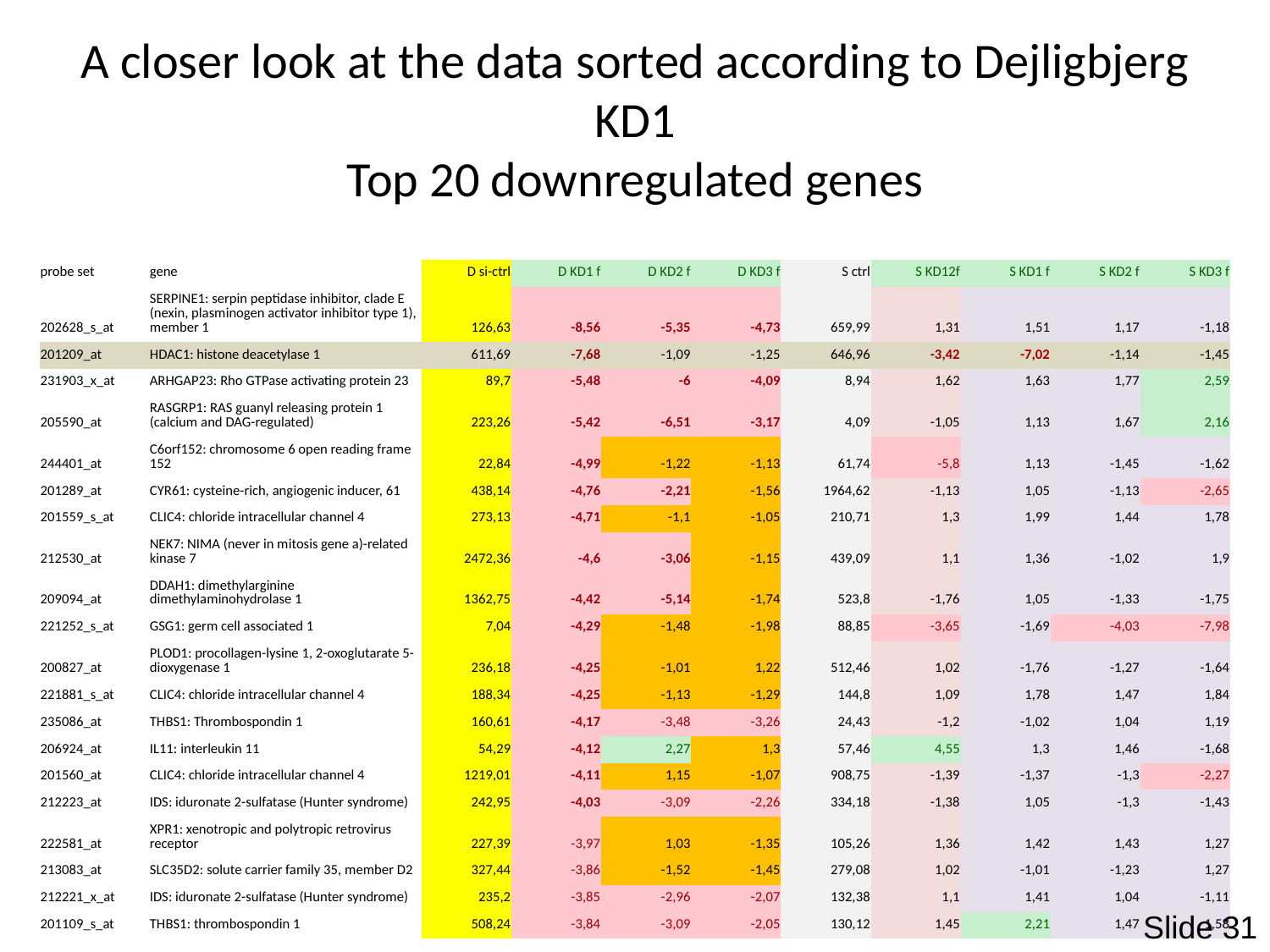

# A closer look at the data sorted according to Dejligbjerg KD1 Top 20 downregulated genes
| probe set | gene | D si-ctrl | D KD1 f | D KD2 f | D KD3 f | S ctrl | S KD12f | S KD1 f | S KD2 f | S KD3 f |
| --- | --- | --- | --- | --- | --- | --- | --- | --- | --- | --- |
| 202628\_s\_at | SERPINE1: serpin peptidase inhibitor, clade E (nexin, plasminogen activator inhibitor type 1), member 1 | 126,63 | -8,56 | -5,35 | -4,73 | 659,99 | 1,31 | 1,51 | 1,17 | -1,18 |
| 201209\_at | HDAC1: histone deacetylase 1 | 611,69 | -7,68 | -1,09 | -1,25 | 646,96 | -3,42 | -7,02 | -1,14 | -1,45 |
| 231903\_x\_at | ARHGAP23: Rho GTPase activating protein 23 | 89,7 | -5,48 | -6 | -4,09 | 8,94 | 1,62 | 1,63 | 1,77 | 2,59 |
| 205590\_at | RASGRP1: RAS guanyl releasing protein 1 (calcium and DAG-regulated) | 223,26 | -5,42 | -6,51 | -3,17 | 4,09 | -1,05 | 1,13 | 1,67 | 2,16 |
| 244401\_at | C6orf152: chromosome 6 open reading frame 152 | 22,84 | -4,99 | -1,22 | -1,13 | 61,74 | -5,8 | 1,13 | -1,45 | -1,62 |
| 201289\_at | CYR61: cysteine-rich, angiogenic inducer, 61 | 438,14 | -4,76 | -2,21 | -1,56 | 1964,62 | -1,13 | 1,05 | -1,13 | -2,65 |
| 201559\_s\_at | CLIC4: chloride intracellular channel 4 | 273,13 | -4,71 | -1,1 | -1,05 | 210,71 | 1,3 | 1,99 | 1,44 | 1,78 |
| 212530\_at | NEK7: NIMA (never in mitosis gene a)-related kinase 7 | 2472,36 | -4,6 | -3,06 | -1,15 | 439,09 | 1,1 | 1,36 | -1,02 | 1,9 |
| 209094\_at | DDAH1: dimethylarginine dimethylaminohydrolase 1 | 1362,75 | -4,42 | -5,14 | -1,74 | 523,8 | -1,76 | 1,05 | -1,33 | -1,75 |
| 221252\_s\_at | GSG1: germ cell associated 1 | 7,04 | -4,29 | -1,48 | -1,98 | 88,85 | -3,65 | -1,69 | -4,03 | -7,98 |
| 200827\_at | PLOD1: procollagen-lysine 1, 2-oxoglutarate 5-dioxygenase 1 | 236,18 | -4,25 | -1,01 | 1,22 | 512,46 | 1,02 | -1,76 | -1,27 | -1,64 |
| 221881\_s\_at | CLIC4: chloride intracellular channel 4 | 188,34 | -4,25 | -1,13 | -1,29 | 144,8 | 1,09 | 1,78 | 1,47 | 1,84 |
| 235086\_at | THBS1: Thrombospondin 1 | 160,61 | -4,17 | -3,48 | -3,26 | 24,43 | -1,2 | -1,02 | 1,04 | 1,19 |
| 206924\_at | IL11: interleukin 11 | 54,29 | -4,12 | 2,27 | 1,3 | 57,46 | 4,55 | 1,3 | 1,46 | -1,68 |
| 201560\_at | CLIC4: chloride intracellular channel 4 | 1219,01 | -4,11 | 1,15 | -1,07 | 908,75 | -1,39 | -1,37 | -1,3 | -2,27 |
| 212223\_at | IDS: iduronate 2-sulfatase (Hunter syndrome) | 242,95 | -4,03 | -3,09 | -2,26 | 334,18 | -1,38 | 1,05 | -1,3 | -1,43 |
| 222581\_at | XPR1: xenotropic and polytropic retrovirus receptor | 227,39 | -3,97 | 1,03 | -1,35 | 105,26 | 1,36 | 1,42 | 1,43 | 1,27 |
| 213083\_at | SLC35D2: solute carrier family 35, member D2 | 327,44 | -3,86 | -1,52 | -1,45 | 279,08 | 1,02 | -1,01 | -1,23 | 1,27 |
| 212221\_x\_at | IDS: iduronate 2-sulfatase (Hunter syndrome) | 235,2 | -3,85 | -2,96 | -2,07 | 132,38 | 1,1 | 1,41 | 1,04 | -1,11 |
| 201109\_s\_at | THBS1: thrombospondin 1 | 508,24 | -3,84 | -3,09 | -2,05 | 130,12 | 1,45 | 2,21 | 1,47 | 1,58 |
Slide <number>

## Slide 32
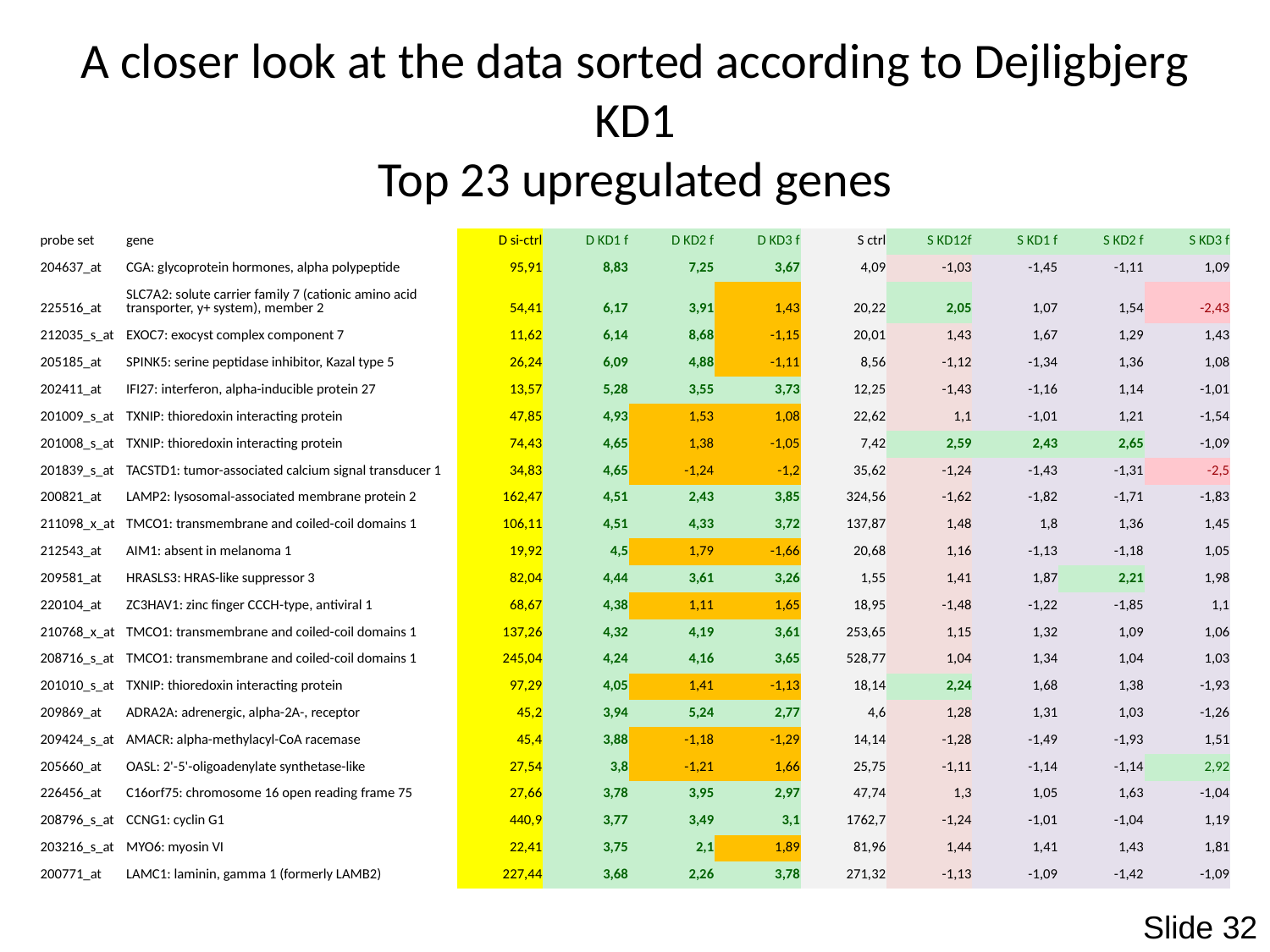

# A closer look at the data sorted according to Dejligbjerg KD1 Top 23 upregulated genes
| probe set | gene | D si-ctrl | D KD1 f | D KD2 f | D KD3 f | S ctrl | S KD12f | S KD1 f | S KD2 f | S KD3 f |
| --- | --- | --- | --- | --- | --- | --- | --- | --- | --- | --- |
| 204637\_at | CGA: glycoprotein hormones, alpha polypeptide | 95,91 | 8,83 | 7,25 | 3,67 | 4,09 | -1,03 | -1,45 | -1,11 | 1,09 |
| 225516\_at | SLC7A2: solute carrier family 7 (cationic amino acid transporter, y+ system), member 2 | 54,41 | 6,17 | 3,91 | 1,43 | 20,22 | 2,05 | 1,07 | 1,54 | -2,43 |
| 212035\_s\_at | EXOC7: exocyst complex component 7 | 11,62 | 6,14 | 8,68 | -1,15 | 20,01 | 1,43 | 1,67 | 1,29 | 1,43 |
| 205185\_at | SPINK5: serine peptidase inhibitor, Kazal type 5 | 26,24 | 6,09 | 4,88 | -1,11 | 8,56 | -1,12 | -1,34 | 1,36 | 1,08 |
| 202411\_at | IFI27: interferon, alpha-inducible protein 27 | 13,57 | 5,28 | 3,55 | 3,73 | 12,25 | -1,43 | -1,16 | 1,14 | -1,01 |
| 201009\_s\_at | TXNIP: thioredoxin interacting protein | 47,85 | 4,93 | 1,53 | 1,08 | 22,62 | 1,1 | -1,01 | 1,21 | -1,54 |
| 201008\_s\_at | TXNIP: thioredoxin interacting protein | 74,43 | 4,65 | 1,38 | -1,05 | 7,42 | 2,59 | 2,43 | 2,65 | -1,09 |
| 201839\_s\_at | TACSTD1: tumor-associated calcium signal transducer 1 | 34,83 | 4,65 | -1,24 | -1,2 | 35,62 | -1,24 | -1,43 | -1,31 | -2,5 |
| 200821\_at | LAMP2: lysosomal-associated membrane protein 2 | 162,47 | 4,51 | 2,43 | 3,85 | 324,56 | -1,62 | -1,82 | -1,71 | -1,83 |
| 211098\_x\_at | TMCO1: transmembrane and coiled-coil domains 1 | 106,11 | 4,51 | 4,33 | 3,72 | 137,87 | 1,48 | 1,8 | 1,36 | 1,45 |
| 212543\_at | AIM1: absent in melanoma 1 | 19,92 | 4,5 | 1,79 | -1,66 | 20,68 | 1,16 | -1,13 | -1,18 | 1,05 |
| 209581\_at | HRASLS3: HRAS-like suppressor 3 | 82,04 | 4,44 | 3,61 | 3,26 | 1,55 | 1,41 | 1,87 | 2,21 | 1,98 |
| 220104\_at | ZC3HAV1: zinc finger CCCH-type, antiviral 1 | 68,67 | 4,38 | 1,11 | 1,65 | 18,95 | -1,48 | -1,22 | -1,85 | 1,1 |
| 210768\_x\_at | TMCO1: transmembrane and coiled-coil domains 1 | 137,26 | 4,32 | 4,19 | 3,61 | 253,65 | 1,15 | 1,32 | 1,09 | 1,06 |
| 208716\_s\_at | TMCO1: transmembrane and coiled-coil domains 1 | 245,04 | 4,24 | 4,16 | 3,65 | 528,77 | 1,04 | 1,34 | 1,04 | 1,03 |
| 201010\_s\_at | TXNIP: thioredoxin interacting protein | 97,29 | 4,05 | 1,41 | -1,13 | 18,14 | 2,24 | 1,68 | 1,38 | -1,93 |
| 209869\_at | ADRA2A: adrenergic, alpha-2A-, receptor | 45,2 | 3,94 | 5,24 | 2,77 | 4,6 | 1,28 | 1,31 | 1,03 | -1,26 |
| 209424\_s\_at | AMACR: alpha-methylacyl-CoA racemase | 45,4 | 3,88 | -1,18 | -1,29 | 14,14 | -1,28 | -1,49 | -1,93 | 1,51 |
| 205660\_at | OASL: 2'-5'-oligoadenylate synthetase-like | 27,54 | 3,8 | -1,21 | 1,66 | 25,75 | -1,11 | -1,14 | -1,14 | 2,92 |
| 226456\_at | C16orf75: chromosome 16 open reading frame 75 | 27,66 | 3,78 | 3,95 | 2,97 | 47,74 | 1,3 | 1,05 | 1,63 | -1,04 |
| 208796\_s\_at | CCNG1: cyclin G1 | 440,9 | 3,77 | 3,49 | 3,1 | 1762,7 | -1,24 | -1,01 | -1,04 | 1,19 |
| 203216\_s\_at | MYO6: myosin VI | 22,41 | 3,75 | 2,1 | 1,89 | 81,96 | 1,44 | 1,41 | 1,43 | 1,81 |
| 200771\_at | LAMC1: laminin, gamma 1 (formerly LAMB2) | 227,44 | 3,68 | 2,26 | 3,78 | 271,32 | -1,13 | -1,09 | -1,42 | -1,09 |
Slide <number>

## Slide 33
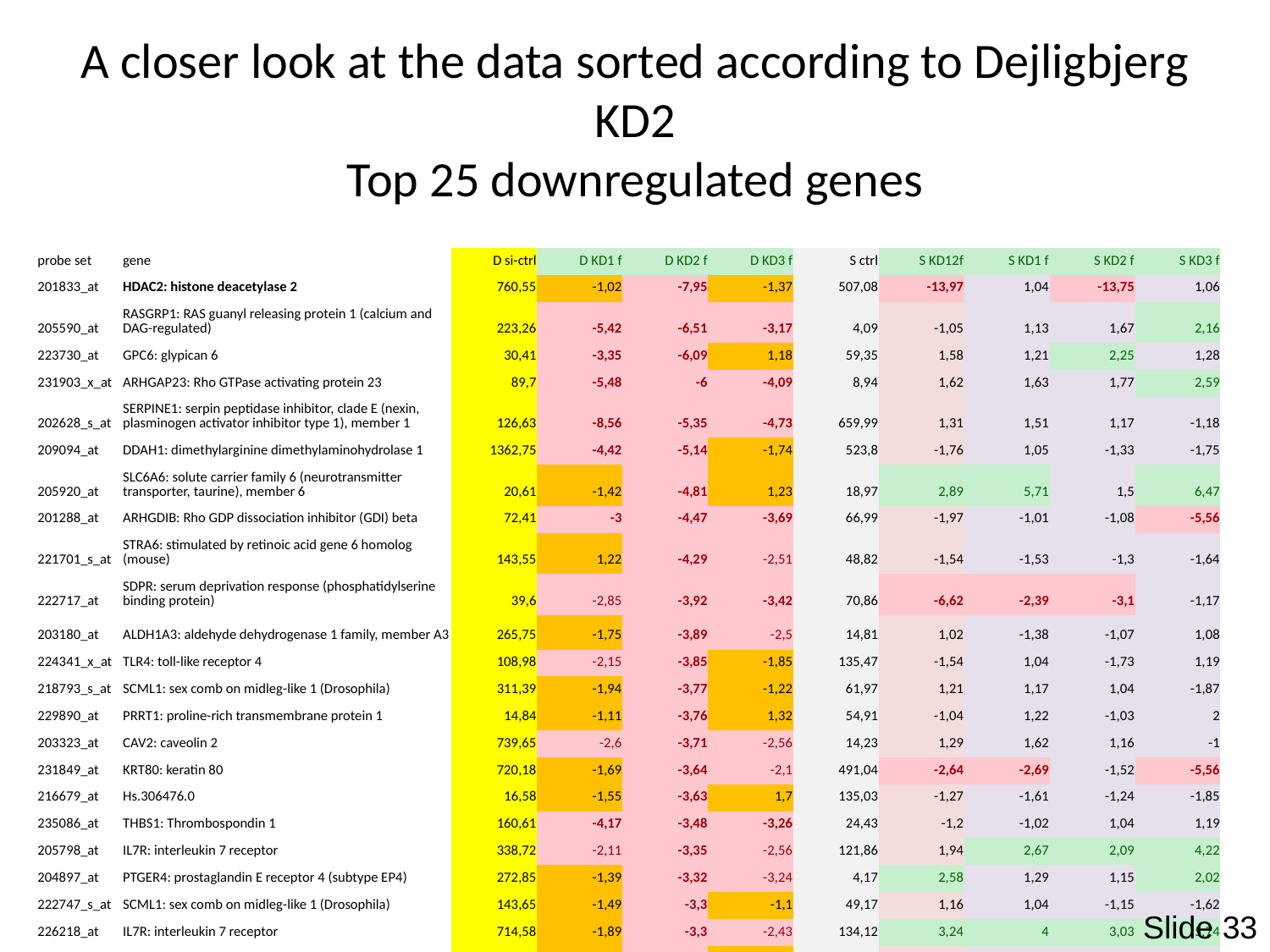

# A closer look at the data sorted according to Dejligbjerg KD2 Top 25 downregulated genes
| probe set | gene | D si-ctrl | D KD1 f | D KD2 f | D KD3 f | S ctrl | S KD12f | S KD1 f | S KD2 f | S KD3 f |
| --- | --- | --- | --- | --- | --- | --- | --- | --- | --- | --- |
| 201833\_at | HDAC2: histone deacetylase 2 | 760,55 | -1,02 | -7,95 | -1,37 | 507,08 | -13,97 | 1,04 | -13,75 | 1,06 |
| 205590\_at | RASGRP1: RAS guanyl releasing protein 1 (calcium and DAG-regulated) | 223,26 | -5,42 | -6,51 | -3,17 | 4,09 | -1,05 | 1,13 | 1,67 | 2,16 |
| 223730\_at | GPC6: glypican 6 | 30,41 | -3,35 | -6,09 | 1,18 | 59,35 | 1,58 | 1,21 | 2,25 | 1,28 |
| 231903\_x\_at | ARHGAP23: Rho GTPase activating protein 23 | 89,7 | -5,48 | -6 | -4,09 | 8,94 | 1,62 | 1,63 | 1,77 | 2,59 |
| 202628\_s\_at | SERPINE1: serpin peptidase inhibitor, clade E (nexin, plasminogen activator inhibitor type 1), member 1 | 126,63 | -8,56 | -5,35 | -4,73 | 659,99 | 1,31 | 1,51 | 1,17 | -1,18 |
| 209094\_at | DDAH1: dimethylarginine dimethylaminohydrolase 1 | 1362,75 | -4,42 | -5,14 | -1,74 | 523,8 | -1,76 | 1,05 | -1,33 | -1,75 |
| 205920\_at | SLC6A6: solute carrier family 6 (neurotransmitter transporter, taurine), member 6 | 20,61 | -1,42 | -4,81 | 1,23 | 18,97 | 2,89 | 5,71 | 1,5 | 6,47 |
| 201288\_at | ARHGDIB: Rho GDP dissociation inhibitor (GDI) beta | 72,41 | -3 | -4,47 | -3,69 | 66,99 | -1,97 | -1,01 | -1,08 | -5,56 |
| 221701\_s\_at | STRA6: stimulated by retinoic acid gene 6 homolog (mouse) | 143,55 | 1,22 | -4,29 | -2,51 | 48,82 | -1,54 | -1,53 | -1,3 | -1,64 |
| 222717\_at | SDPR: serum deprivation response (phosphatidylserine binding protein) | 39,6 | -2,85 | -3,92 | -3,42 | 70,86 | -6,62 | -2,39 | -3,1 | -1,17 |
| 203180\_at | ALDH1A3: aldehyde dehydrogenase 1 family, member A3 | 265,75 | -1,75 | -3,89 | -2,5 | 14,81 | 1,02 | -1,38 | -1,07 | 1,08 |
| 224341\_x\_at | TLR4: toll-like receptor 4 | 108,98 | -2,15 | -3,85 | -1,85 | 135,47 | -1,54 | 1,04 | -1,73 | 1,19 |
| 218793\_s\_at | SCML1: sex comb on midleg-like 1 (Drosophila) | 311,39 | -1,94 | -3,77 | -1,22 | 61,97 | 1,21 | 1,17 | 1,04 | -1,87 |
| 229890\_at | PRRT1: proline-rich transmembrane protein 1 | 14,84 | -1,11 | -3,76 | 1,32 | 54,91 | -1,04 | 1,22 | -1,03 | 2 |
| 203323\_at | CAV2: caveolin 2 | 739,65 | -2,6 | -3,71 | -2,56 | 14,23 | 1,29 | 1,62 | 1,16 | -1 |
| 231849\_at | KRT80: keratin 80 | 720,18 | -1,69 | -3,64 | -2,1 | 491,04 | -2,64 | -2,69 | -1,52 | -5,56 |
| 216679\_at | Hs.306476.0 | 16,58 | -1,55 | -3,63 | 1,7 | 135,03 | -1,27 | -1,61 | -1,24 | -1,85 |
| 235086\_at | THBS1: Thrombospondin 1 | 160,61 | -4,17 | -3,48 | -3,26 | 24,43 | -1,2 | -1,02 | 1,04 | 1,19 |
| 205798\_at | IL7R: interleukin 7 receptor | 338,72 | -2,11 | -3,35 | -2,56 | 121,86 | 1,94 | 2,67 | 2,09 | 4,22 |
| 204897\_at | PTGER4: prostaglandin E receptor 4 (subtype EP4) | 272,85 | -1,39 | -3,32 | -3,24 | 4,17 | 2,58 | 1,29 | 1,15 | 2,02 |
| 222747\_s\_at | SCML1: sex comb on midleg-like 1 (Drosophila) | 143,65 | -1,49 | -3,3 | -1,1 | 49,17 | 1,16 | 1,04 | -1,15 | -1,62 |
| 226218\_at | IL7R: interleukin 7 receptor | 714,58 | -1,89 | -3,3 | -2,43 | 134,12 | 3,24 | 4 | 3,03 | 5,74 |
| 225397\_at | CCDC32: coiled-coil domain containing 32 | 96,13 | -1,27 | -3,25 | -1,59 | 50,82 | -1,09 | -1,11 | 1,06 | 1,36 |
| 206147\_x\_at | SCML2: sex comb on midleg-like 2 (Drosophila) | 637,35 | -1,72 | -3,12 | -1,5 | 130,02 | 1,07 | 1,03 | -1,05 | -1,47 |
| 209904\_at | TNNC1: troponin C type 1 (slow) | 54,74 | -2,37 | -3,11 | -1,44 | 281,82 | -7,9 | -13,64 | -6,27 | -14,75 |
Slide <number>

## Slide 34
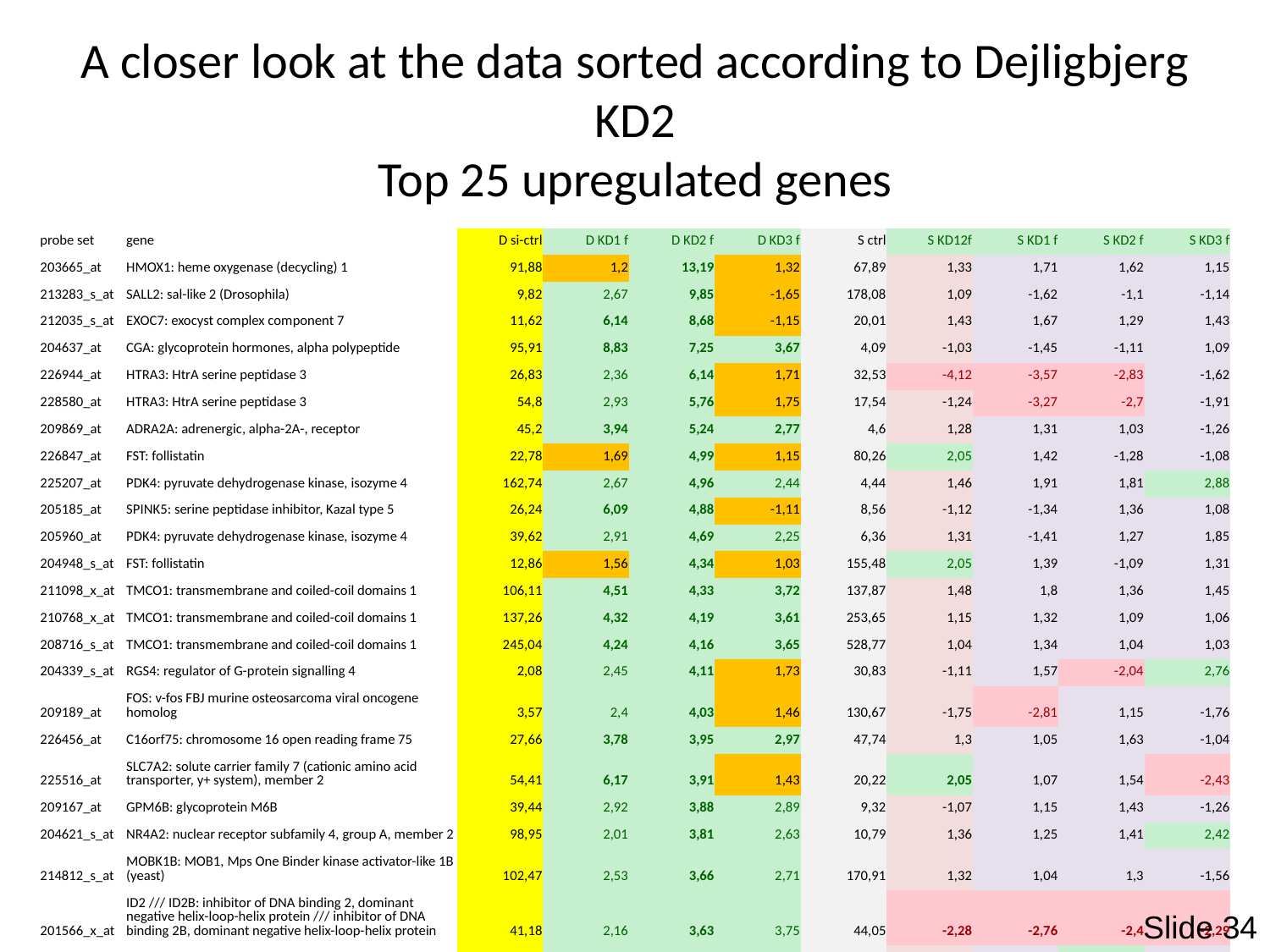

# A closer look at the data sorted according to Dejligbjerg KD2 Top 25 upregulated genes
| probe set | gene | D si-ctrl | D KD1 f | D KD2 f | D KD3 f | S ctrl | S KD12f | S KD1 f | S KD2 f | S KD3 f |
| --- | --- | --- | --- | --- | --- | --- | --- | --- | --- | --- |
| 203665\_at | HMOX1: heme oxygenase (decycling) 1 | 91,88 | 1,2 | 13,19 | 1,32 | 67,89 | 1,33 | 1,71 | 1,62 | 1,15 |
| 213283\_s\_at | SALL2: sal-like 2 (Drosophila) | 9,82 | 2,67 | 9,85 | -1,65 | 178,08 | 1,09 | -1,62 | -1,1 | -1,14 |
| 212035\_s\_at | EXOC7: exocyst complex component 7 | 11,62 | 6,14 | 8,68 | -1,15 | 20,01 | 1,43 | 1,67 | 1,29 | 1,43 |
| 204637\_at | CGA: glycoprotein hormones, alpha polypeptide | 95,91 | 8,83 | 7,25 | 3,67 | 4,09 | -1,03 | -1,45 | -1,11 | 1,09 |
| 226944\_at | HTRA3: HtrA serine peptidase 3 | 26,83 | 2,36 | 6,14 | 1,71 | 32,53 | -4,12 | -3,57 | -2,83 | -1,62 |
| 228580\_at | HTRA3: HtrA serine peptidase 3 | 54,8 | 2,93 | 5,76 | 1,75 | 17,54 | -1,24 | -3,27 | -2,7 | -1,91 |
| 209869\_at | ADRA2A: adrenergic, alpha-2A-, receptor | 45,2 | 3,94 | 5,24 | 2,77 | 4,6 | 1,28 | 1,31 | 1,03 | -1,26 |
| 226847\_at | FST: follistatin | 22,78 | 1,69 | 4,99 | 1,15 | 80,26 | 2,05 | 1,42 | -1,28 | -1,08 |
| 225207\_at | PDK4: pyruvate dehydrogenase kinase, isozyme 4 | 162,74 | 2,67 | 4,96 | 2,44 | 4,44 | 1,46 | 1,91 | 1,81 | 2,88 |
| 205185\_at | SPINK5: serine peptidase inhibitor, Kazal type 5 | 26,24 | 6,09 | 4,88 | -1,11 | 8,56 | -1,12 | -1,34 | 1,36 | 1,08 |
| 205960\_at | PDK4: pyruvate dehydrogenase kinase, isozyme 4 | 39,62 | 2,91 | 4,69 | 2,25 | 6,36 | 1,31 | -1,41 | 1,27 | 1,85 |
| 204948\_s\_at | FST: follistatin | 12,86 | 1,56 | 4,34 | 1,03 | 155,48 | 2,05 | 1,39 | -1,09 | 1,31 |
| 211098\_x\_at | TMCO1: transmembrane and coiled-coil domains 1 | 106,11 | 4,51 | 4,33 | 3,72 | 137,87 | 1,48 | 1,8 | 1,36 | 1,45 |
| 210768\_x\_at | TMCO1: transmembrane and coiled-coil domains 1 | 137,26 | 4,32 | 4,19 | 3,61 | 253,65 | 1,15 | 1,32 | 1,09 | 1,06 |
| 208716\_s\_at | TMCO1: transmembrane and coiled-coil domains 1 | 245,04 | 4,24 | 4,16 | 3,65 | 528,77 | 1,04 | 1,34 | 1,04 | 1,03 |
| 204339\_s\_at | RGS4: regulator of G-protein signalling 4 | 2,08 | 2,45 | 4,11 | 1,73 | 30,83 | -1,11 | 1,57 | -2,04 | 2,76 |
| 209189\_at | FOS: v-fos FBJ murine osteosarcoma viral oncogene homolog | 3,57 | 2,4 | 4,03 | 1,46 | 130,67 | -1,75 | -2,81 | 1,15 | -1,76 |
| 226456\_at | C16orf75: chromosome 16 open reading frame 75 | 27,66 | 3,78 | 3,95 | 2,97 | 47,74 | 1,3 | 1,05 | 1,63 | -1,04 |
| 225516\_at | SLC7A2: solute carrier family 7 (cationic amino acid transporter, y+ system), member 2 | 54,41 | 6,17 | 3,91 | 1,43 | 20,22 | 2,05 | 1,07 | 1,54 | -2,43 |
| 209167\_at | GPM6B: glycoprotein M6B | 39,44 | 2,92 | 3,88 | 2,89 | 9,32 | -1,07 | 1,15 | 1,43 | -1,26 |
| 204621\_s\_at | NR4A2: nuclear receptor subfamily 4, group A, member 2 | 98,95 | 2,01 | 3,81 | 2,63 | 10,79 | 1,36 | 1,25 | 1,41 | 2,42 |
| 214812\_s\_at | MOBK1B: MOB1, Mps One Binder kinase activator-like 1B (yeast) | 102,47 | 2,53 | 3,66 | 2,71 | 170,91 | 1,32 | 1,04 | 1,3 | -1,56 |
| 201566\_x\_at | ID2 /// ID2B: inhibitor of DNA binding 2, dominant negative helix-loop-helix protein /// inhibitor of DNA binding 2B, dominant negative helix-loop-helix protein | 41,18 | 2,16 | 3,63 | 3,75 | 44,05 | -2,28 | -2,76 | -2,4 | -2,29 |
| 209581\_at | HRASLS3: HRAS-like suppressor 3 | 82,04 | 4,44 | 3,61 | 3,26 | 1,55 | 1,41 | 1,87 | 2,21 | 1,98 |
| 201565\_s\_at | ID2: inhibitor of DNA binding 2, dominant negative helix-loop-helix protein | 71,33 | 2,35 | 3,61 | 3,46 | 94,05 | -3,32 | -2,95 | -2,38 | -2,06 |
Slide <number>

## Slide 35
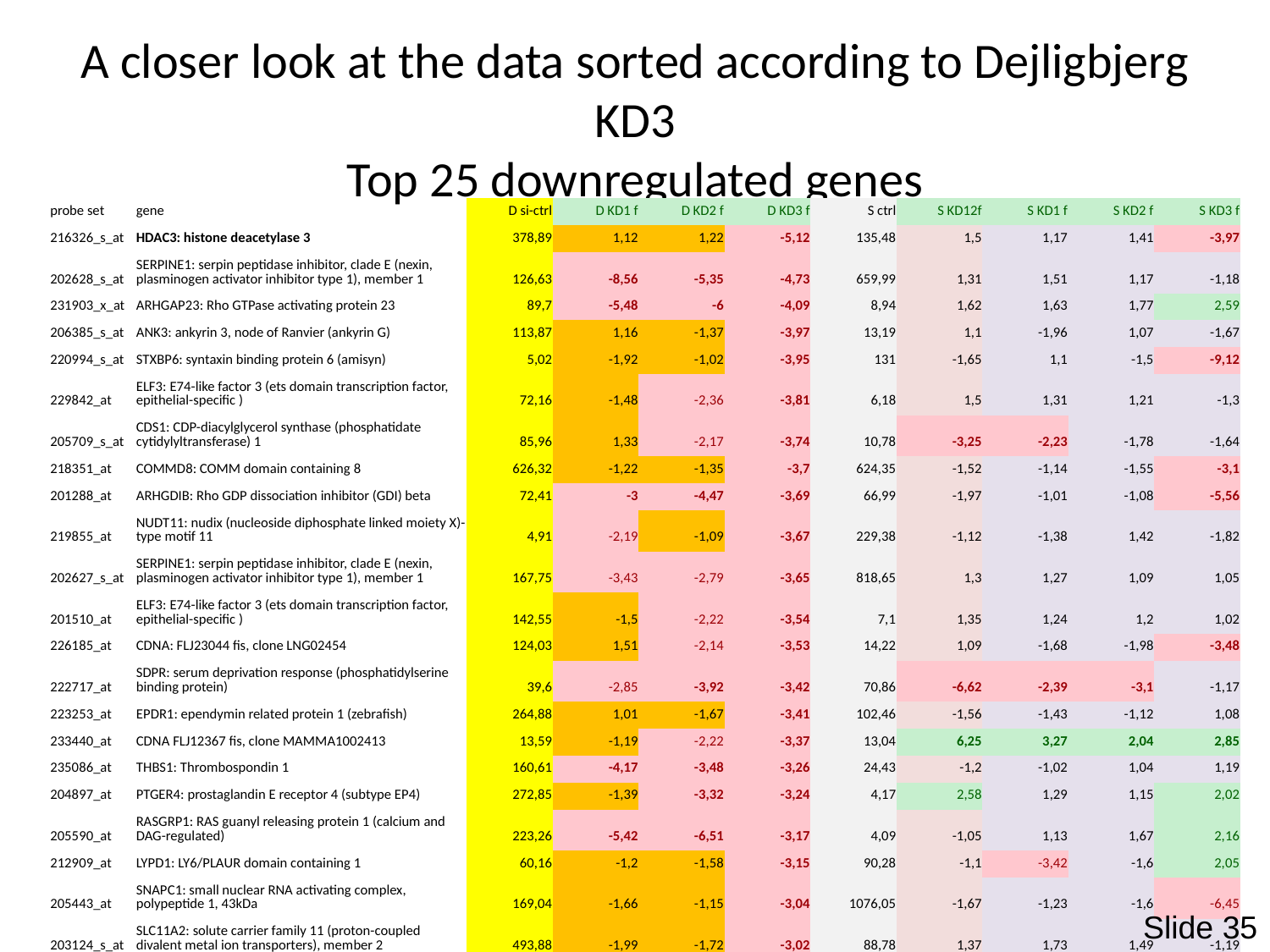

# A closer look at the data sorted according to Dejligbjerg KD3 Top 25 downregulated genes
| probe set | gene | D si-ctrl | D KD1 f | D KD2 f | D KD3 f | S ctrl | S KD12f | S KD1 f | S KD2 f | S KD3 f |
| --- | --- | --- | --- | --- | --- | --- | --- | --- | --- | --- |
| 216326\_s\_at | HDAC3: histone deacetylase 3 | 378,89 | 1,12 | 1,22 | -5,12 | 135,48 | 1,5 | 1,17 | 1,41 | -3,97 |
| 202628\_s\_at | SERPINE1: serpin peptidase inhibitor, clade E (nexin, plasminogen activator inhibitor type 1), member 1 | 126,63 | -8,56 | -5,35 | -4,73 | 659,99 | 1,31 | 1,51 | 1,17 | -1,18 |
| 231903\_x\_at | ARHGAP23: Rho GTPase activating protein 23 | 89,7 | -5,48 | -6 | -4,09 | 8,94 | 1,62 | 1,63 | 1,77 | 2,59 |
| 206385\_s\_at | ANK3: ankyrin 3, node of Ranvier (ankyrin G) | 113,87 | 1,16 | -1,37 | -3,97 | 13,19 | 1,1 | -1,96 | 1,07 | -1,67 |
| 220994\_s\_at | STXBP6: syntaxin binding protein 6 (amisyn) | 5,02 | -1,92 | -1,02 | -3,95 | 131 | -1,65 | 1,1 | -1,5 | -9,12 |
| 229842\_at | ELF3: E74-like factor 3 (ets domain transcription factor, epithelial-specific ) | 72,16 | -1,48 | -2,36 | -3,81 | 6,18 | 1,5 | 1,31 | 1,21 | -1,3 |
| 205709\_s\_at | CDS1: CDP-diacylglycerol synthase (phosphatidate cytidylyltransferase) 1 | 85,96 | 1,33 | -2,17 | -3,74 | 10,78 | -3,25 | -2,23 | -1,78 | -1,64 |
| 218351\_at | COMMD8: COMM domain containing 8 | 626,32 | -1,22 | -1,35 | -3,7 | 624,35 | -1,52 | -1,14 | -1,55 | -3,1 |
| 201288\_at | ARHGDIB: Rho GDP dissociation inhibitor (GDI) beta | 72,41 | -3 | -4,47 | -3,69 | 66,99 | -1,97 | -1,01 | -1,08 | -5,56 |
| 219855\_at | NUDT11: nudix (nucleoside diphosphate linked moiety X)-type motif 11 | 4,91 | -2,19 | -1,09 | -3,67 | 229,38 | -1,12 | -1,38 | 1,42 | -1,82 |
| 202627\_s\_at | SERPINE1: serpin peptidase inhibitor, clade E (nexin, plasminogen activator inhibitor type 1), member 1 | 167,75 | -3,43 | -2,79 | -3,65 | 818,65 | 1,3 | 1,27 | 1,09 | 1,05 |
| 201510\_at | ELF3: E74-like factor 3 (ets domain transcription factor, epithelial-specific ) | 142,55 | -1,5 | -2,22 | -3,54 | 7,1 | 1,35 | 1,24 | 1,2 | 1,02 |
| 226185\_at | CDNA: FLJ23044 fis, clone LNG02454 | 124,03 | 1,51 | -2,14 | -3,53 | 14,22 | 1,09 | -1,68 | -1,98 | -3,48 |
| 222717\_at | SDPR: serum deprivation response (phosphatidylserine binding protein) | 39,6 | -2,85 | -3,92 | -3,42 | 70,86 | -6,62 | -2,39 | -3,1 | -1,17 |
| 223253\_at | EPDR1: ependymin related protein 1 (zebrafish) | 264,88 | 1,01 | -1,67 | -3,41 | 102,46 | -1,56 | -1,43 | -1,12 | 1,08 |
| 233440\_at | CDNA FLJ12367 fis, clone MAMMA1002413 | 13,59 | -1,19 | -2,22 | -3,37 | 13,04 | 6,25 | 3,27 | 2,04 | 2,85 |
| 235086\_at | THBS1: Thrombospondin 1 | 160,61 | -4,17 | -3,48 | -3,26 | 24,43 | -1,2 | -1,02 | 1,04 | 1,19 |
| 204897\_at | PTGER4: prostaglandin E receptor 4 (subtype EP4) | 272,85 | -1,39 | -3,32 | -3,24 | 4,17 | 2,58 | 1,29 | 1,15 | 2,02 |
| 205590\_at | RASGRP1: RAS guanyl releasing protein 1 (calcium and DAG-regulated) | 223,26 | -5,42 | -6,51 | -3,17 | 4,09 | -1,05 | 1,13 | 1,67 | 2,16 |
| 212909\_at | LYPD1: LY6/PLAUR domain containing 1 | 60,16 | -1,2 | -1,58 | -3,15 | 90,28 | -1,1 | -3,42 | -1,6 | 2,05 |
| 205443\_at | SNAPC1: small nuclear RNA activating complex, polypeptide 1, 43kDa | 169,04 | -1,66 | -1,15 | -3,04 | 1076,05 | -1,67 | -1,23 | -1,6 | -6,45 |
| 203124\_s\_at | SLC11A2: solute carrier family 11 (proton-coupled divalent metal ion transporters), member 2 | 493,88 | -1,99 | -1,72 | -3,02 | 88,78 | 1,37 | 1,73 | 1,49 | -1,19 |
| 223370\_at | PLEKHA3: pleckstrin homology domain containing, family A (phosphoinositide binding specific) member 3 | 298,5 | -1,79 | -1,14 | -3 | 158,41 | 1,05 | 1,22 | -1,11 | -1,28 |
Slide <number>

## Slide 36
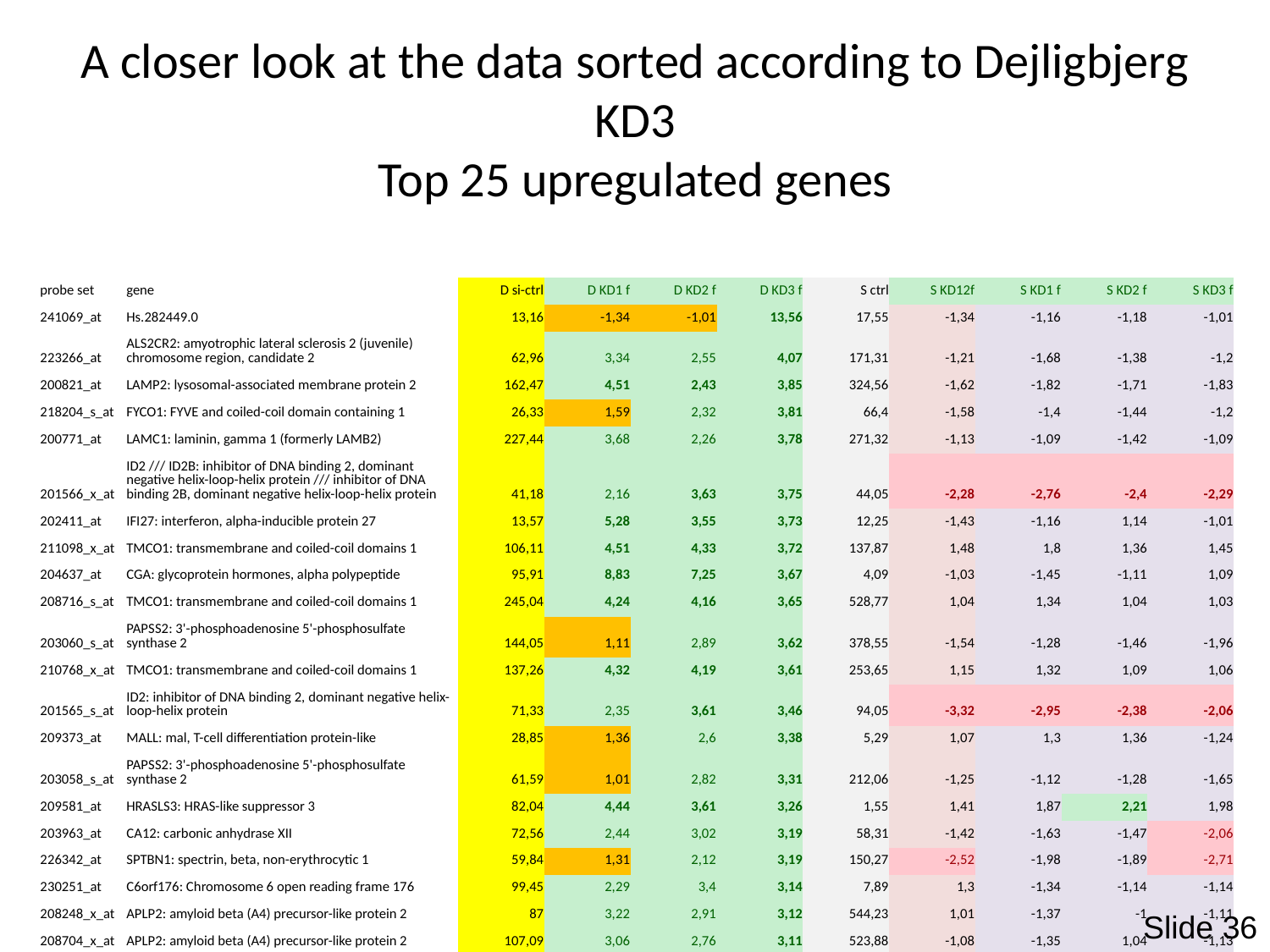

# A closer look at the data sorted according to Dejligbjerg KD3 Top 25 upregulated genes
| probe set | gene | D si-ctrl | D KD1 f | D KD2 f | D KD3 f | S ctrl | S KD12f | S KD1 f | S KD2 f | S KD3 f |
| --- | --- | --- | --- | --- | --- | --- | --- | --- | --- | --- |
| 241069\_at | Hs.282449.0 | 13,16 | -1,34 | -1,01 | 13,56 | 17,55 | -1,34 | -1,16 | -1,18 | -1,01 |
| 223266\_at | ALS2CR2: amyotrophic lateral sclerosis 2 (juvenile) chromosome region, candidate 2 | 62,96 | 3,34 | 2,55 | 4,07 | 171,31 | -1,21 | -1,68 | -1,38 | -1,2 |
| 200821\_at | LAMP2: lysosomal-associated membrane protein 2 | 162,47 | 4,51 | 2,43 | 3,85 | 324,56 | -1,62 | -1,82 | -1,71 | -1,83 |
| 218204\_s\_at | FYCO1: FYVE and coiled-coil domain containing 1 | 26,33 | 1,59 | 2,32 | 3,81 | 66,4 | -1,58 | -1,4 | -1,44 | -1,2 |
| 200771\_at | LAMC1: laminin, gamma 1 (formerly LAMB2) | 227,44 | 3,68 | 2,26 | 3,78 | 271,32 | -1,13 | -1,09 | -1,42 | -1,09 |
| 201566\_x\_at | ID2 /// ID2B: inhibitor of DNA binding 2, dominant negative helix-loop-helix protein /// inhibitor of DNA binding 2B, dominant negative helix-loop-helix protein | 41,18 | 2,16 | 3,63 | 3,75 | 44,05 | -2,28 | -2,76 | -2,4 | -2,29 |
| 202411\_at | IFI27: interferon, alpha-inducible protein 27 | 13,57 | 5,28 | 3,55 | 3,73 | 12,25 | -1,43 | -1,16 | 1,14 | -1,01 |
| 211098\_x\_at | TMCO1: transmembrane and coiled-coil domains 1 | 106,11 | 4,51 | 4,33 | 3,72 | 137,87 | 1,48 | 1,8 | 1,36 | 1,45 |
| 204637\_at | CGA: glycoprotein hormones, alpha polypeptide | 95,91 | 8,83 | 7,25 | 3,67 | 4,09 | -1,03 | -1,45 | -1,11 | 1,09 |
| 208716\_s\_at | TMCO1: transmembrane and coiled-coil domains 1 | 245,04 | 4,24 | 4,16 | 3,65 | 528,77 | 1,04 | 1,34 | 1,04 | 1,03 |
| 203060\_s\_at | PAPSS2: 3'-phosphoadenosine 5'-phosphosulfate synthase 2 | 144,05 | 1,11 | 2,89 | 3,62 | 378,55 | -1,54 | -1,28 | -1,46 | -1,96 |
| 210768\_x\_at | TMCO1: transmembrane and coiled-coil domains 1 | 137,26 | 4,32 | 4,19 | 3,61 | 253,65 | 1,15 | 1,32 | 1,09 | 1,06 |
| 201565\_s\_at | ID2: inhibitor of DNA binding 2, dominant negative helix-loop-helix protein | 71,33 | 2,35 | 3,61 | 3,46 | 94,05 | -3,32 | -2,95 | -2,38 | -2,06 |
| 209373\_at | MALL: mal, T-cell differentiation protein-like | 28,85 | 1,36 | 2,6 | 3,38 | 5,29 | 1,07 | 1,3 | 1,36 | -1,24 |
| 203058\_s\_at | PAPSS2: 3'-phosphoadenosine 5'-phosphosulfate synthase 2 | 61,59 | 1,01 | 2,82 | 3,31 | 212,06 | -1,25 | -1,12 | -1,28 | -1,65 |
| 209581\_at | HRASLS3: HRAS-like suppressor 3 | 82,04 | 4,44 | 3,61 | 3,26 | 1,55 | 1,41 | 1,87 | 2,21 | 1,98 |
| 203963\_at | CA12: carbonic anhydrase XII | 72,56 | 2,44 | 3,02 | 3,19 | 58,31 | -1,42 | -1,63 | -1,47 | -2,06 |
| 226342\_at | SPTBN1: spectrin, beta, non-erythrocytic 1 | 59,84 | 1,31 | 2,12 | 3,19 | 150,27 | -2,52 | -1,98 | -1,89 | -2,71 |
| 230251\_at | C6orf176: Chromosome 6 open reading frame 176 | 99,45 | 2,29 | 3,4 | 3,14 | 7,89 | 1,3 | -1,34 | -1,14 | -1,14 |
| 208248\_x\_at | APLP2: amyloid beta (A4) precursor-like protein 2 | 87 | 3,22 | 2,91 | 3,12 | 544,23 | 1,01 | -1,37 | -1 | -1,11 |
| 208704\_x\_at | APLP2: amyloid beta (A4) precursor-like protein 2 | 107,09 | 3,06 | 2,76 | 3,11 | 523,88 | -1,08 | -1,35 | 1,04 | -1,13 |
| 208796\_s\_at | CCNG1: cyclin G1 | 440,9 | 3,77 | 3,49 | 3,1 | 1762,7 | -1,24 | -1,01 | -1,04 | 1,19 |
| 208937\_s\_at | ID1: inhibitor of DNA binding 1, dominant negative helix-loop-helix protein | 930,31 | 1,56 | 2,07 | 3,03 | 1419,07 | -1,23 | -1,57 | -1,56 | -1,57 |
| 223843\_at | SCARA3: scavenger receptor class A, member 3 | 119,6 | 1,85 | 3,2 | 3,02 | 76,19 | -4,8 | -2,46 | -4,21 | -6,35 |
Slide <number>

## Slide 37
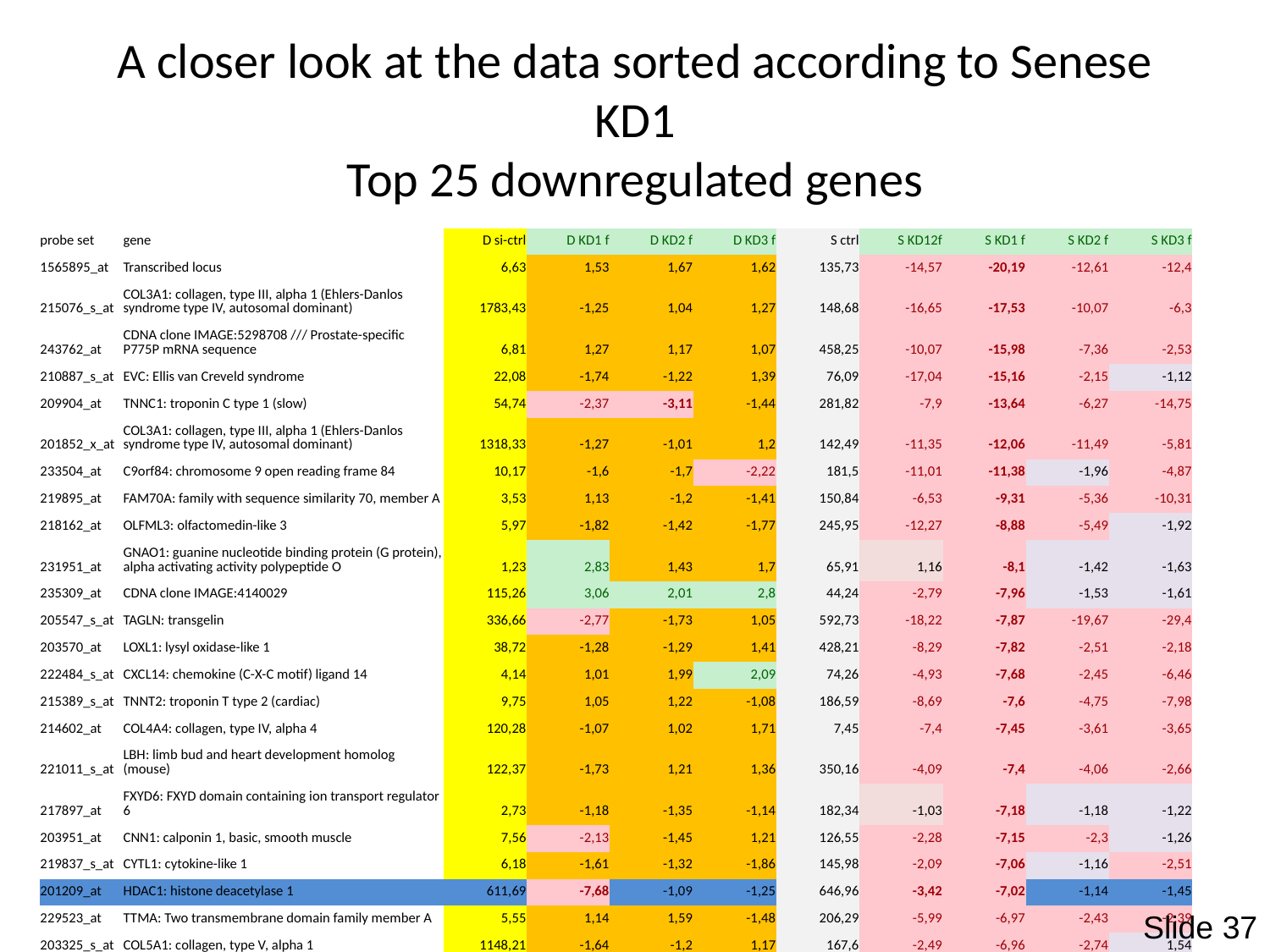

# A closer look at the data sorted according to Senese KD1 Top 25 downregulated genes
| probe set | gene | D si-ctrl | D KD1 f | D KD2 f | D KD3 f | S ctrl | S KD12f | S KD1 f | S KD2 f | S KD3 f |
| --- | --- | --- | --- | --- | --- | --- | --- | --- | --- | --- |
| 1565895\_at | Transcribed locus | 6,63 | 1,53 | 1,67 | 1,62 | 135,73 | -14,57 | -20,19 | -12,61 | -12,4 |
| 215076\_s\_at | COL3A1: collagen, type III, alpha 1 (Ehlers-Danlos syndrome type IV, autosomal dominant) | 1783,43 | -1,25 | 1,04 | 1,27 | 148,68 | -16,65 | -17,53 | -10,07 | -6,3 |
| 243762\_at | CDNA clone IMAGE:5298708 /// Prostate-specific P775P mRNA sequence | 6,81 | 1,27 | 1,17 | 1,07 | 458,25 | -10,07 | -15,98 | -7,36 | -2,53 |
| 210887\_s\_at | EVC: Ellis van Creveld syndrome | 22,08 | -1,74 | -1,22 | 1,39 | 76,09 | -17,04 | -15,16 | -2,15 | -1,12 |
| 209904\_at | TNNC1: troponin C type 1 (slow) | 54,74 | -2,37 | -3,11 | -1,44 | 281,82 | -7,9 | -13,64 | -6,27 | -14,75 |
| 201852\_x\_at | COL3A1: collagen, type III, alpha 1 (Ehlers-Danlos syndrome type IV, autosomal dominant) | 1318,33 | -1,27 | -1,01 | 1,2 | 142,49 | -11,35 | -12,06 | -11,49 | -5,81 |
| 233504\_at | C9orf84: chromosome 9 open reading frame 84 | 10,17 | -1,6 | -1,7 | -2,22 | 181,5 | -11,01 | -11,38 | -1,96 | -4,87 |
| 219895\_at | FAM70A: family with sequence similarity 70, member A | 3,53 | 1,13 | -1,2 | -1,41 | 150,84 | -6,53 | -9,31 | -5,36 | -10,31 |
| 218162\_at | OLFML3: olfactomedin-like 3 | 5,97 | -1,82 | -1,42 | -1,77 | 245,95 | -12,27 | -8,88 | -5,49 | -1,92 |
| 231951\_at | GNAO1: guanine nucleotide binding protein (G protein), alpha activating activity polypeptide O | 1,23 | 2,83 | 1,43 | 1,7 | 65,91 | 1,16 | -8,1 | -1,42 | -1,63 |
| 235309\_at | CDNA clone IMAGE:4140029 | 115,26 | 3,06 | 2,01 | 2,8 | 44,24 | -2,79 | -7,96 | -1,53 | -1,61 |
| 205547\_s\_at | TAGLN: transgelin | 336,66 | -2,77 | -1,73 | 1,05 | 592,73 | -18,22 | -7,87 | -19,67 | -29,4 |
| 203570\_at | LOXL1: lysyl oxidase-like 1 | 38,72 | -1,28 | -1,29 | 1,41 | 428,21 | -8,29 | -7,82 | -2,51 | -2,18 |
| 222484\_s\_at | CXCL14: chemokine (C-X-C motif) ligand 14 | 4,14 | 1,01 | 1,99 | 2,09 | 74,26 | -4,93 | -7,68 | -2,45 | -6,46 |
| 215389\_s\_at | TNNT2: troponin T type 2 (cardiac) | 9,75 | 1,05 | 1,22 | -1,08 | 186,59 | -8,69 | -7,6 | -4,75 | -7,98 |
| 214602\_at | COL4A4: collagen, type IV, alpha 4 | 120,28 | -1,07 | 1,02 | 1,71 | 7,45 | -7,4 | -7,45 | -3,61 | -3,65 |
| 221011\_s\_at | LBH: limb bud and heart development homolog (mouse) | 122,37 | -1,73 | 1,21 | 1,36 | 350,16 | -4,09 | -7,4 | -4,06 | -2,66 |
| 217897\_at | FXYD6: FXYD domain containing ion transport regulator 6 | 2,73 | -1,18 | -1,35 | -1,14 | 182,34 | -1,03 | -7,18 | -1,18 | -1,22 |
| 203951\_at | CNN1: calponin 1, basic, smooth muscle | 7,56 | -2,13 | -1,45 | 1,21 | 126,55 | -2,28 | -7,15 | -2,3 | -1,26 |
| 219837\_s\_at | CYTL1: cytokine-like 1 | 6,18 | -1,61 | -1,32 | -1,86 | 145,98 | -2,09 | -7,06 | -1,16 | -2,51 |
| 201209\_at | HDAC1: histone deacetylase 1 | 611,69 | -7,68 | -1,09 | -1,25 | 646,96 | -3,42 | -7,02 | -1,14 | -1,45 |
| 229523\_at | TTMA: Two transmembrane domain family member A | 5,55 | 1,14 | 1,59 | -1,48 | 206,29 | -5,99 | -6,97 | -2,43 | -2,39 |
| 203325\_s\_at | COL5A1: collagen, type V, alpha 1 | 1148,21 | -1,64 | -1,2 | 1,17 | 167,6 | -2,49 | -6,96 | -2,74 | 1,54 |
| 203071\_at | SEMA3B: sema domain, immunoglobulin domain (Ig), short basic domain, secreted, (semaphorin) 3B | 64,85 | 1,03 | 1,09 | 1,45 | 60,5 | -3,22 | -6,89 | -3,49 | -2,43 |
| 1562926\_at | Homo sapiens, clone IMAGE:4512650, mRNA | 6,43 | -1,03 | 1,07 | -1,32 | 203,93 | -4,2 | -6,68 | -3,62 | -4,37 |
Slide <number>

## Slide 38
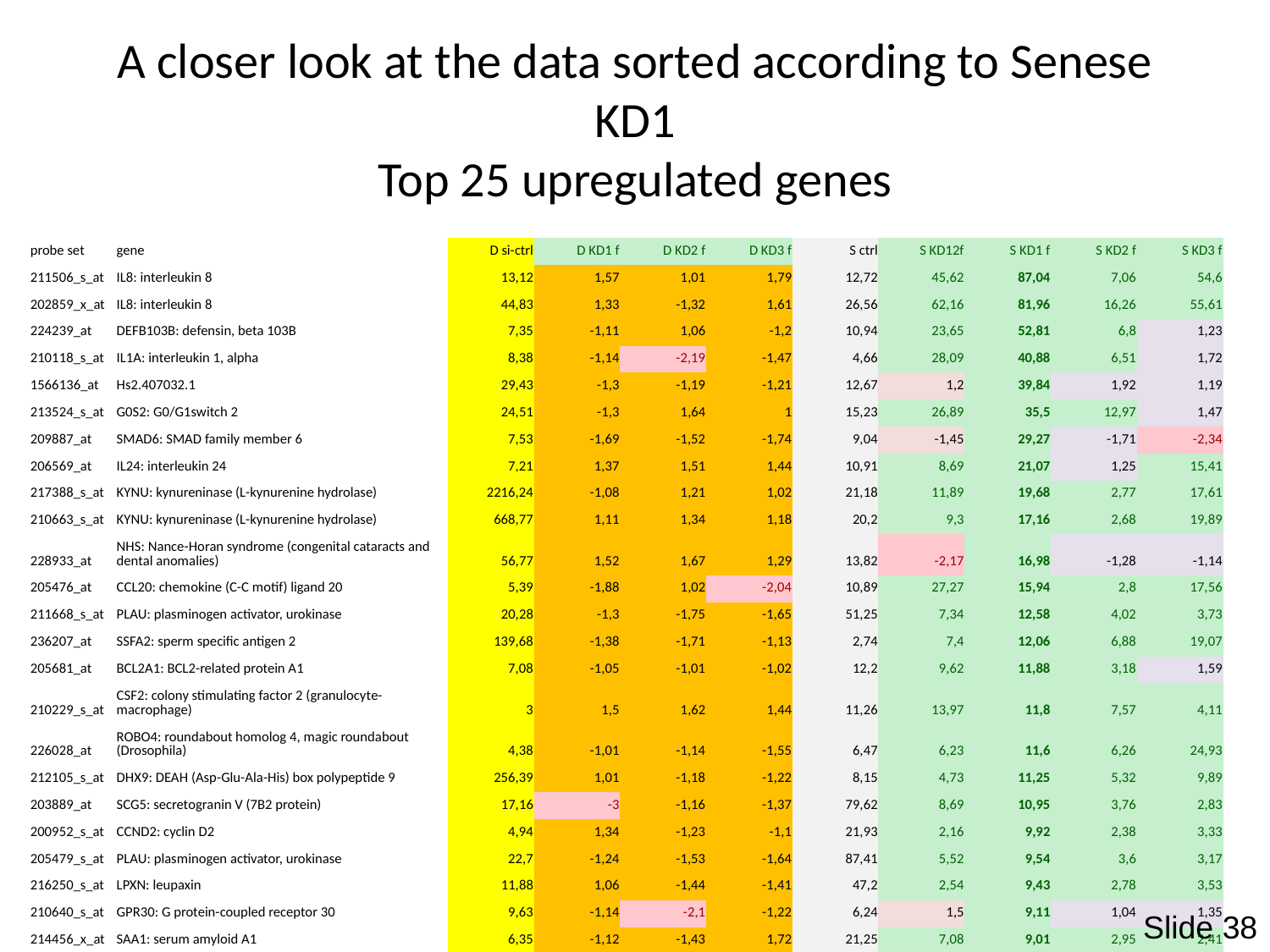

# A closer look at the data sorted according to Senese KD1 Top 25 upregulated genes
| probe set | gene | D si-ctrl | D KD1 f | D KD2 f | D KD3 f | S ctrl | S KD12f | S KD1 f | S KD2 f | S KD3 f |
| --- | --- | --- | --- | --- | --- | --- | --- | --- | --- | --- |
| 211506\_s\_at | IL8: interleukin 8 | 13,12 | 1,57 | 1,01 | 1,79 | 12,72 | 45,62 | 87,04 | 7,06 | 54,6 |
| 202859\_x\_at | IL8: interleukin 8 | 44,83 | 1,33 | -1,32 | 1,61 | 26,56 | 62,16 | 81,96 | 16,26 | 55,61 |
| 224239\_at | DEFB103B: defensin, beta 103B | 7,35 | -1,11 | 1,06 | -1,2 | 10,94 | 23,65 | 52,81 | 6,8 | 1,23 |
| 210118\_s\_at | IL1A: interleukin 1, alpha | 8,38 | -1,14 | -2,19 | -1,47 | 4,66 | 28,09 | 40,88 | 6,51 | 1,72 |
| 1566136\_at | Hs2.407032.1 | 29,43 | -1,3 | -1,19 | -1,21 | 12,67 | 1,2 | 39,84 | 1,92 | 1,19 |
| 213524\_s\_at | G0S2: G0/G1switch 2 | 24,51 | -1,3 | 1,64 | 1 | 15,23 | 26,89 | 35,5 | 12,97 | 1,47 |
| 209887\_at | SMAD6: SMAD family member 6 | 7,53 | -1,69 | -1,52 | -1,74 | 9,04 | -1,45 | 29,27 | -1,71 | -2,34 |
| 206569\_at | IL24: interleukin 24 | 7,21 | 1,37 | 1,51 | 1,44 | 10,91 | 8,69 | 21,07 | 1,25 | 15,41 |
| 217388\_s\_at | KYNU: kynureninase (L-kynurenine hydrolase) | 2216,24 | -1,08 | 1,21 | 1,02 | 21,18 | 11,89 | 19,68 | 2,77 | 17,61 |
| 210663\_s\_at | KYNU: kynureninase (L-kynurenine hydrolase) | 668,77 | 1,11 | 1,34 | 1,18 | 20,2 | 9,3 | 17,16 | 2,68 | 19,89 |
| 228933\_at | NHS: Nance-Horan syndrome (congenital cataracts and dental anomalies) | 56,77 | 1,52 | 1,67 | 1,29 | 13,82 | -2,17 | 16,98 | -1,28 | -1,14 |
| 205476\_at | CCL20: chemokine (C-C motif) ligand 20 | 5,39 | -1,88 | 1,02 | -2,04 | 10,89 | 27,27 | 15,94 | 2,8 | 17,56 |
| 211668\_s\_at | PLAU: plasminogen activator, urokinase | 20,28 | -1,3 | -1,75 | -1,65 | 51,25 | 7,34 | 12,58 | 4,02 | 3,73 |
| 236207\_at | SSFA2: sperm specific antigen 2 | 139,68 | -1,38 | -1,71 | -1,13 | 2,74 | 7,4 | 12,06 | 6,88 | 19,07 |
| 205681\_at | BCL2A1: BCL2-related protein A1 | 7,08 | -1,05 | -1,01 | -1,02 | 12,2 | 9,62 | 11,88 | 3,18 | 1,59 |
| 210229\_s\_at | CSF2: colony stimulating factor 2 (granulocyte-macrophage) | 3 | 1,5 | 1,62 | 1,44 | 11,26 | 13,97 | 11,8 | 7,57 | 4,11 |
| 226028\_at | ROBO4: roundabout homolog 4, magic roundabout (Drosophila) | 4,38 | -1,01 | -1,14 | -1,55 | 6,47 | 6,23 | 11,6 | 6,26 | 24,93 |
| 212105\_s\_at | DHX9: DEAH (Asp-Glu-Ala-His) box polypeptide 9 | 256,39 | 1,01 | -1,18 | -1,22 | 8,15 | 4,73 | 11,25 | 5,32 | 9,89 |
| 203889\_at | SCG5: secretogranin V (7B2 protein) | 17,16 | -3 | -1,16 | -1,37 | 79,62 | 8,69 | 10,95 | 3,76 | 2,83 |
| 200952\_s\_at | CCND2: cyclin D2 | 4,94 | 1,34 | -1,23 | -1,1 | 21,93 | 2,16 | 9,92 | 2,38 | 3,33 |
| 205479\_s\_at | PLAU: plasminogen activator, urokinase | 22,7 | -1,24 | -1,53 | -1,64 | 87,41 | 5,52 | 9,54 | 3,6 | 3,17 |
| 216250\_s\_at | LPXN: leupaxin | 11,88 | 1,06 | -1,44 | -1,41 | 47,2 | 2,54 | 9,43 | 2,78 | 3,53 |
| 210640\_s\_at | GPR30: G protein-coupled receptor 30 | 9,63 | -1,14 | -2,1 | -1,22 | 6,24 | 1,5 | 9,11 | 1,04 | 1,35 |
| 214456\_x\_at | SAA1: serum amyloid A1 | 6,35 | -1,12 | -1,43 | 1,72 | 21,25 | 7,08 | 9,01 | 2,95 | 2,41 |
| 210457\_x\_at | HMGA1: high mobility group AT-hook 1 | 41,27 | -1,17 | -1,07 | -1,37 | 54,53 | 5,77 | 8,96 | 4,65 | 4 |
Slide <number>

## Slide 39
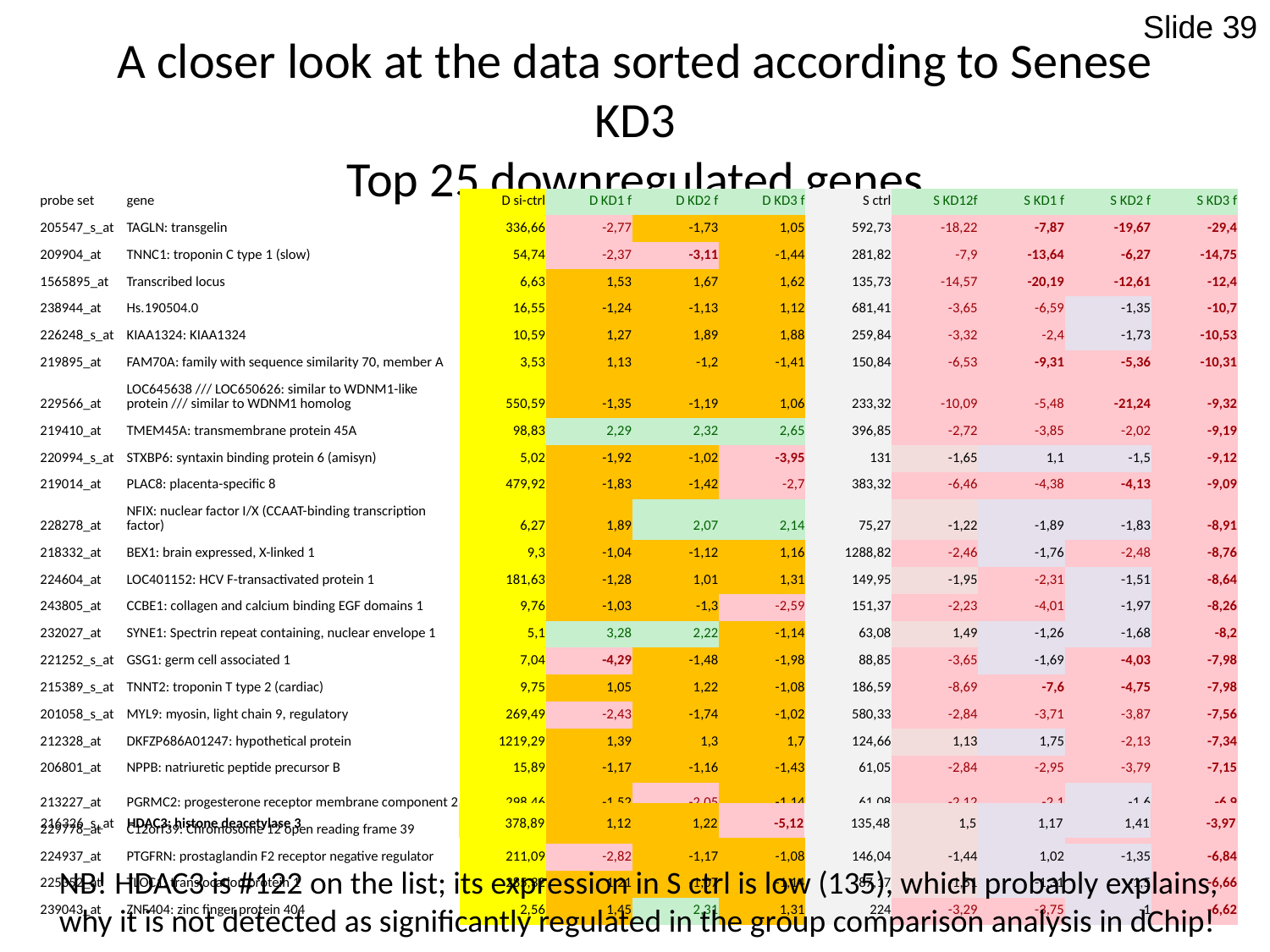

Slide <number>
# A closer look at the data sorted according to Senese KD3 Top 25 downregulated genes
| probe set | gene | D si-ctrl | D KD1 f | D KD2 f | D KD3 f | S ctrl | S KD12f | S KD1 f | S KD2 f | S KD3 f |
| --- | --- | --- | --- | --- | --- | --- | --- | --- | --- | --- |
| 205547\_s\_at | TAGLN: transgelin | 336,66 | -2,77 | -1,73 | 1,05 | 592,73 | -18,22 | -7,87 | -19,67 | -29,4 |
| 209904\_at | TNNC1: troponin C type 1 (slow) | 54,74 | -2,37 | -3,11 | -1,44 | 281,82 | -7,9 | -13,64 | -6,27 | -14,75 |
| 1565895\_at | Transcribed locus | 6,63 | 1,53 | 1,67 | 1,62 | 135,73 | -14,57 | -20,19 | -12,61 | -12,4 |
| 238944\_at | Hs.190504.0 | 16,55 | -1,24 | -1,13 | 1,12 | 681,41 | -3,65 | -6,59 | -1,35 | -10,7 |
| 226248\_s\_at | KIAA1324: KIAA1324 | 10,59 | 1,27 | 1,89 | 1,88 | 259,84 | -3,32 | -2,4 | -1,73 | -10,53 |
| 219895\_at | FAM70A: family with sequence similarity 70, member A | 3,53 | 1,13 | -1,2 | -1,41 | 150,84 | -6,53 | -9,31 | -5,36 | -10,31 |
| 229566\_at | LOC645638 /// LOC650626: similar to WDNM1-like protein /// similar to WDNM1 homolog | 550,59 | -1,35 | -1,19 | 1,06 | 233,32 | -10,09 | -5,48 | -21,24 | -9,32 |
| 219410\_at | TMEM45A: transmembrane protein 45A | 98,83 | 2,29 | 2,32 | 2,65 | 396,85 | -2,72 | -3,85 | -2,02 | -9,19 |
| 220994\_s\_at | STXBP6: syntaxin binding protein 6 (amisyn) | 5,02 | -1,92 | -1,02 | -3,95 | 131 | -1,65 | 1,1 | -1,5 | -9,12 |
| 219014\_at | PLAC8: placenta-specific 8 | 479,92 | -1,83 | -1,42 | -2,7 | 383,32 | -6,46 | -4,38 | -4,13 | -9,09 |
| 228278\_at | NFIX: nuclear factor I/X (CCAAT-binding transcription factor) | 6,27 | 1,89 | 2,07 | 2,14 | 75,27 | -1,22 | -1,89 | -1,83 | -8,91 |
| 218332\_at | BEX1: brain expressed, X-linked 1 | 9,3 | -1,04 | -1,12 | 1,16 | 1288,82 | -2,46 | -1,76 | -2,48 | -8,76 |
| 224604\_at | LOC401152: HCV F-transactivated protein 1 | 181,63 | -1,28 | 1,01 | 1,31 | 149,95 | -1,95 | -2,31 | -1,51 | -8,64 |
| 243805\_at | CCBE1: collagen and calcium binding EGF domains 1 | 9,76 | -1,03 | -1,3 | -2,59 | 151,37 | -2,23 | -4,01 | -1,97 | -8,26 |
| 232027\_at | SYNE1: Spectrin repeat containing, nuclear envelope 1 | 5,1 | 3,28 | 2,22 | -1,14 | 63,08 | 1,49 | -1,26 | -1,68 | -8,2 |
| 221252\_s\_at | GSG1: germ cell associated 1 | 7,04 | -4,29 | -1,48 | -1,98 | 88,85 | -3,65 | -1,69 | -4,03 | -7,98 |
| 215389\_s\_at | TNNT2: troponin T type 2 (cardiac) | 9,75 | 1,05 | 1,22 | -1,08 | 186,59 | -8,69 | -7,6 | -4,75 | -7,98 |
| 201058\_s\_at | MYL9: myosin, light chain 9, regulatory | 269,49 | -2,43 | -1,74 | -1,02 | 580,33 | -2,84 | -3,71 | -3,87 | -7,56 |
| 212328\_at | DKFZP686A01247: hypothetical protein | 1219,29 | 1,39 | 1,3 | 1,7 | 124,66 | 1,13 | 1,75 | -2,13 | -7,34 |
| 206801\_at | NPPB: natriuretic peptide precursor B | 15,89 | -1,17 | -1,16 | -1,43 | 61,05 | -2,84 | -2,95 | -3,79 | -7,15 |
| 213227\_at | PGRMC2: progesterone receptor membrane component 2 | 298,46 | -1,52 | -2,05 | -1,14 | 61,08 | -2,12 | -2,1 | -1,6 | -6,9 |
| 229778\_at | C12orf39: Chromosome 12 open reading frame 39 | 4,73 | 1,41 | -1,48 | -1,51 | 77,85 | -1,46 | -1,13 | -2,09 | -6,86 |
| 224937\_at | PTGFRN: prostaglandin F2 receptor negative regulator | 211,09 | -2,82 | -1,17 | -1,08 | 146,04 | -1,44 | 1,02 | -1,35 | -6,84 |
| 225352\_at | TLOC1: translocation protein 1 | 285,32 | 1,21 | -1,07 | -1,14 | 87,17 | -1,51 | -1,31 | -1,5 | -6,66 |
| 239043\_at | ZNF404: zinc finger protein 404 | 2,56 | 1,45 | 2,31 | 1,31 | 224 | -3,29 | -3,75 | -1 | -6,62 |
| 216326\_s\_at | HDAC3: histone deacetylase 3 | 378,89 | 1,12 | 1,22 | -5,12 | 135,48 | 1,5 | 1,17 | 1,41 | -3,97 |
| --- | --- | --- | --- | --- | --- | --- | --- | --- | --- | --- |
NB! HDAC3 is #122 on the list; its expression in S ctrl is low (135), which probably explains,
why it is not detected as significantly regulated in the group comparison analysis in dChip!

## Slide 40
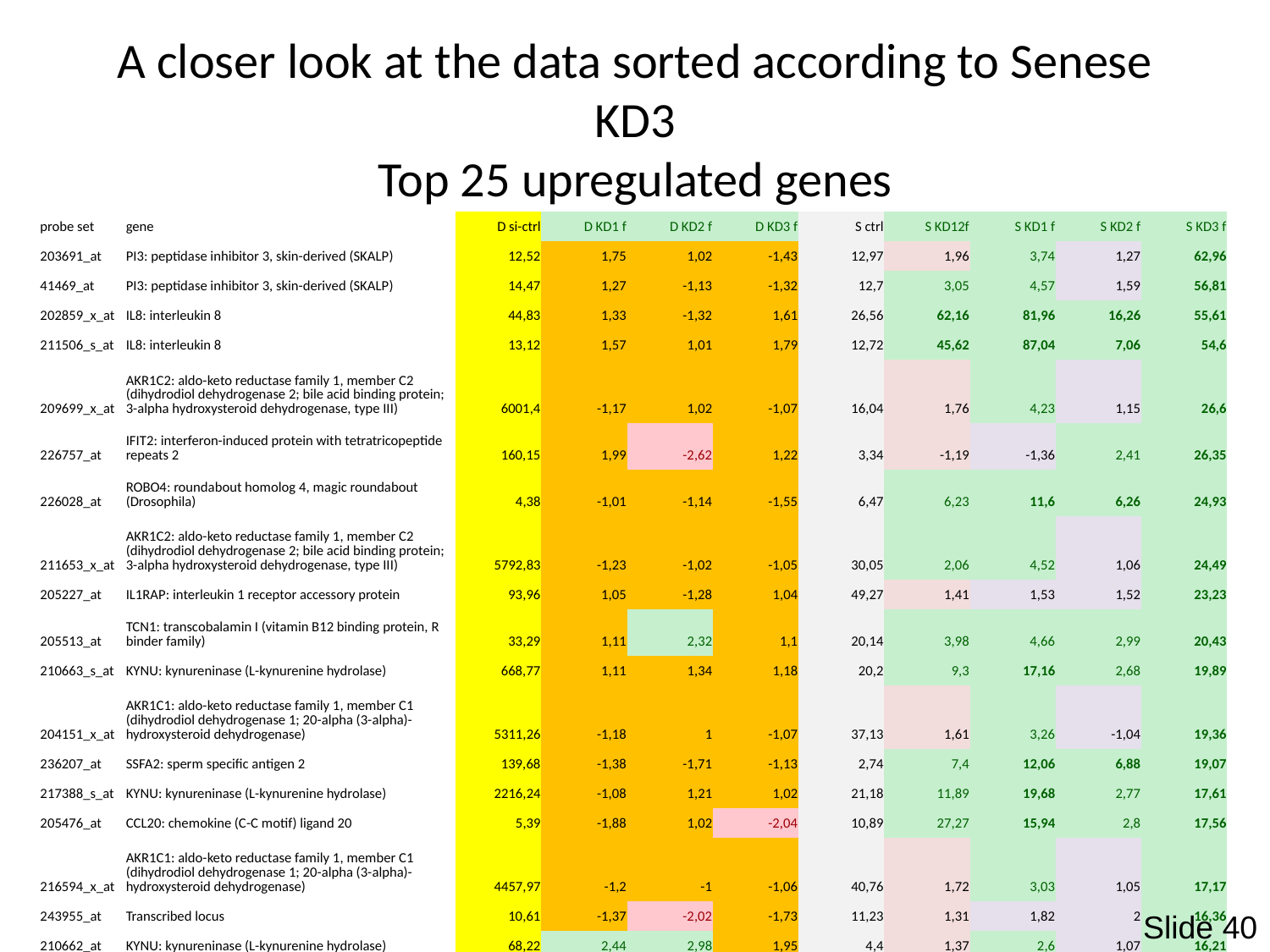

# A closer look at the data sorted according to Senese KD3 Top 25 upregulated genes
| probe set | gene | D si-ctrl | D KD1 f | D KD2 f | D KD3 f | S ctrl | S KD12f | S KD1 f | S KD2 f | S KD3 f |
| --- | --- | --- | --- | --- | --- | --- | --- | --- | --- | --- |
| 203691\_at | PI3: peptidase inhibitor 3, skin-derived (SKALP) | 12,52 | 1,75 | 1,02 | -1,43 | 12,97 | 1,96 | 3,74 | 1,27 | 62,96 |
| 41469\_at | PI3: peptidase inhibitor 3, skin-derived (SKALP) | 14,47 | 1,27 | -1,13 | -1,32 | 12,7 | 3,05 | 4,57 | 1,59 | 56,81 |
| 202859\_x\_at | IL8: interleukin 8 | 44,83 | 1,33 | -1,32 | 1,61 | 26,56 | 62,16 | 81,96 | 16,26 | 55,61 |
| 211506\_s\_at | IL8: interleukin 8 | 13,12 | 1,57 | 1,01 | 1,79 | 12,72 | 45,62 | 87,04 | 7,06 | 54,6 |
| 209699\_x\_at | AKR1C2: aldo-keto reductase family 1, member C2 (dihydrodiol dehydrogenase 2; bile acid binding protein; 3-alpha hydroxysteroid dehydrogenase, type III) | 6001,4 | -1,17 | 1,02 | -1,07 | 16,04 | 1,76 | 4,23 | 1,15 | 26,6 |
| 226757\_at | IFIT2: interferon-induced protein with tetratricopeptide repeats 2 | 160,15 | 1,99 | -2,62 | 1,22 | 3,34 | -1,19 | -1,36 | 2,41 | 26,35 |
| 226028\_at | ROBO4: roundabout homolog 4, magic roundabout (Drosophila) | 4,38 | -1,01 | -1,14 | -1,55 | 6,47 | 6,23 | 11,6 | 6,26 | 24,93 |
| 211653\_x\_at | AKR1C2: aldo-keto reductase family 1, member C2 (dihydrodiol dehydrogenase 2; bile acid binding protein; 3-alpha hydroxysteroid dehydrogenase, type III) | 5792,83 | -1,23 | -1,02 | -1,05 | 30,05 | 2,06 | 4,52 | 1,06 | 24,49 |
| 205227\_at | IL1RAP: interleukin 1 receptor accessory protein | 93,96 | 1,05 | -1,28 | 1,04 | 49,27 | 1,41 | 1,53 | 1,52 | 23,23 |
| 205513\_at | TCN1: transcobalamin I (vitamin B12 binding protein, R binder family) | 33,29 | 1,11 | 2,32 | 1,1 | 20,14 | 3,98 | 4,66 | 2,99 | 20,43 |
| 210663\_s\_at | KYNU: kynureninase (L-kynurenine hydrolase) | 668,77 | 1,11 | 1,34 | 1,18 | 20,2 | 9,3 | 17,16 | 2,68 | 19,89 |
| 204151\_x\_at | AKR1C1: aldo-keto reductase family 1, member C1 (dihydrodiol dehydrogenase 1; 20-alpha (3-alpha)-hydroxysteroid dehydrogenase) | 5311,26 | -1,18 | 1 | -1,07 | 37,13 | 1,61 | 3,26 | -1,04 | 19,36 |
| 236207\_at | SSFA2: sperm specific antigen 2 | 139,68 | -1,38 | -1,71 | -1,13 | 2,74 | 7,4 | 12,06 | 6,88 | 19,07 |
| 217388\_s\_at | KYNU: kynureninase (L-kynurenine hydrolase) | 2216,24 | -1,08 | 1,21 | 1,02 | 21,18 | 11,89 | 19,68 | 2,77 | 17,61 |
| 205476\_at | CCL20: chemokine (C-C motif) ligand 20 | 5,39 | -1,88 | 1,02 | -2,04 | 10,89 | 27,27 | 15,94 | 2,8 | 17,56 |
| 216594\_x\_at | AKR1C1: aldo-keto reductase family 1, member C1 (dihydrodiol dehydrogenase 1; 20-alpha (3-alpha)-hydroxysteroid dehydrogenase) | 4457,97 | -1,2 | -1 | -1,06 | 40,76 | 1,72 | 3,03 | 1,05 | 17,17 |
| 243955\_at | Transcribed locus | 10,61 | -1,37 | -2,02 | -1,73 | 11,23 | 1,31 | 1,82 | 2 | 16,36 |
| 210662\_at | KYNU: kynureninase (L-kynurenine hydrolase) | 68,22 | 2,44 | 2,98 | 1,95 | 4,4 | 1,37 | 2,6 | 1,07 | 16,21 |
| 214974\_x\_at | CXCL5: chemokine (C-X-C motif) ligand 5 | 3,19 | 1,54 | 1,17 | 1,71 | 5,3 | 17,34 | 7,58 | 2,62 | 15,52 |
| 206569\_at | IL24: interleukin 24 | 7,21 | 1,37 | 1,51 | 1,44 | 10,91 | 8,69 | 21,07 | 1,25 | 15,41 |
| 216867\_s\_at | PDGFA: platelet-derived growth factor alpha polypeptide | 9 | -1,3 | -1,64 | 1,1 | 14,39 | 1,6 | 1,51 | 1,39 | 12,47 |
| 232287\_at | PGBD3: piggyBac transposable element derived 3 | 65,96 | -1,07 | -1,26 | -1,21 | 29,06 | -1,56 | -1,03 | -1,42 | 11,52 |
| 237215\_s\_at | TFRC: transferrin receptor (p90, CD71) | 557,4 | 1,18 | -1,13 | -2,23 | 21,93 | 4,7 | 8,79 | 5,22 | 11,18 |
| 222878\_s\_at | OTUB2: OTU domain, ubiquitin aldehyde binding 2 | 6,01 | 1,51 | 1,09 | 1,26 | 5,69 | 3,62 | 6,95 | 2,76 | 10,6 |
| 205100\_at | GFPT2: glutamine-fructose-6-phosphate transaminase 2 | 79,69 | -2,08 | -1,56 | -1,67 | 47,98 | 2,47 | 1,66 | 2,42 | 10,53 |
NB! IL8, PI3, ROBO4
Slide <number>
